# Revision of the Western Indian Ocean Angel Sharks, Genus *Squatina* (Squatiniformes, Squatinidae), with Description of a New Species and Redescription of the African Angel Shark *Squatina africana* Regan, 1908

**DOI:** 10.3390/biology12070975

**Published:** 2023-07-08

**Authors:** Simon Weigmann, Diego F. B. Vaz, K. V. Akhilesh, Ruth H. Leeney, Gavin J. P. Naylor

**Affiliations:** 1 Elasmo-Lab, Elasmobranch Research Laboratory, 22609 Hamburg, Germany; 2 Harvard Museum of Comparative Zoology, 26 Oxford St, Cambridge, MA 02138, USA; bistonvazd@triton.uog.edu; 3 Guam Ecosystems Collaboratorium Biorepository, Guam EPSCoR, Marine Laboratory, University of Guam, Mangilao, GU 96923, USA; 4 ICAR-Central Marine Fisheries Research Institute, PB No. 1603, Kochi, Kerala 682 018, India; akhikv@gmail.com; 5 Natural History Museum, Cromwell Rd, South Kensington, London SW7 5BD, UK; ruth.leeney@gmail.com; 6 Florida Museum of Natural History, Dickinson Hall, 1659 Museum Road, University of Florida, Gainesville, FL 32611, USA; gnaylor@flmnh.ufl.edu

**Keywords:** Chondrichthyes, Elasmobranchii, angel sharks, systematics, taxonomy, diversity, morphology, PCA, mCT scans, genetics, NADH2, CO1

## Abstract

**Simple Summary:**

Angel sharks (genus *Squatina*) are small- to medium-sized sharks with flattened bodies, that live on the seafloor. Until now, 23 valid species of angel sharks have been identified around the world, of which over half are thought to be facing a moderate to severe risk of extinction. Several juvenile angel sharks were collected by researchers working on the Mascarene Plateau, an elevated area of seabed in the Indian Ocean, in 1988 and 1989. They appeared different in coloration and in body shape and structure to a species known from East Africa and Madagascar, the African angel shark. Additional angel sharks were caught off the western coast of India in 2016 and in the central western Indian Ocean in 2017, including adult individuals. Information on body measurements and skeleton structure were collected, and genetic analyses were also conducted on these sharks and on museum specimens previously identified as African angel sharks. The results indicated that the specimens collected from the Mascarene Plateau and off southwestern India were a species that is new to science. It is genetically and morphologically distinct from the African angel shark; is smaller when born and when fully grown; and lives in a distinctly different area. The newly described species has been named Lea’s angel shark.

**Abstract:**

Sampling efforts on the Saya de Malha Bank (part of the Mascarene Plateau, western Indian Ocean) unveiled three unusual small juvenile angel shark specimens, that were a much paler color than the only known western Indian Ocean species, *Squatina africana* Regan, 1908. However, it took many years before further specimens, including adults of both sexes, and tissue samples were collected. The present manuscript contains a redescription of *S. africana* based on the holotype and additional material, as well as the formal description of the new species of *Squatina*. All specimens of the new species, hereafter referred to as *Squatina leae* sp. nov., were collected in the western Indian Ocean off southwestern India and on the Mascarene Plateau at depths of 100–500 m. The new species differs from *S. africana* in a number of characteristics including its coloration when fresh, smaller size at birth, size at maturity, and adult size, genetic composition, and distribution. Taxonomic characteristics include differences in the morphology of the pectoral skeleton and posterior nasal flap, denticle arrangement and morphology, vertebral counts, trunk width, pectoral–pelvic space, and clasper size. A key to the species of *Squatina* in the Indian Ocean is provided.

## 1. Introduction

Squatiniform sharks (angel sharks) possess distinctly broad, dorsoventrally flattened bodies, a short snout with large mouth and nostrils, eyes on top of the head close to the large spiracles, very large pectoral fins, and a lateral caudal keel [1]. Furthermore, the anterior-most basiventral cartilages are laterally expanded and have curved, dorsally reflected margins [2]. Based on genetic analyses, previous studies have shown that the Squatiniformes form a clade with the Pristiophoriformes and Squaliformes but without the Hexanchiformes [3,4,5]. However, Naylor et al. [5] highlighted that the molecular interrelationships between the Echinorhinidae, Pristiophoriformes, and Squatiniformes remained unclear due to weak support. Interestingly, the current proposed interrelationships appear to differ significantly between published studies [6,7,8], highlighting the need to clarify the understanding of the interrelationships among these groups. In spite of the lack of compelling molecular data, there is morphological support for the interrelationships reported by Naylor et al. [3,4,5] for the Squatiniformes and Pristiophoriformes, e.g., by Shirai [9] based mainly on skeletal and myological features. Nevertheless, Weigmann et al. [10] and Moreira and Carvalho [11] pointed out that, despite sharing many characteristics, the claspers of Squatiniformes and Pristiophoriformes are strikingly different. Weigmann et al. [10] proposed that the differences in clasper morphology between angel sharks and saw sharks could be interpreted as autapomorphic while the similarity in body shape and morphology of the first basiventral cartilages constitutes evidence of an interrelationship.

The monotypic order Squatiniformes, with its monotypic family Squatinidae Bonaparte, 1838 containing the monotypic genus *Squatina* Duméril, 1806, presently contains 23 valid species worldwide. Four of the five Indian Ocean species occur in the eastern Indian Ocean while only one, *S. africana* Regan, 1908, occurs in the western Indian Ocean (updated from [12,13]). Angel sharks are generally small- to medium-sized, demersal sharks. They are known from all three major oceans with the centers of diversity in the western Atlantic and western Pacific and reaching maximum sizes of 787 to 2440 mm in total length (TL) [1,12,14]. Although angel sharks are typically found at shelf and upper slope depths, *S. dumeril* Lesueur, 1818 has been recorded at 1289 m depth [12,15,16].

The taxonomy of angel sharks has mostly been based on external morphological characteristics, such as morphological proportions of the head, pectoral fins, and tail; shape of nasal barbels, pectoral, dorsal, and caudal fins; dorsal coloration; presence or absence of enlarged dorsal midline denticles; and dental formula [15,17,18,19,20,21,22,23,24]. Although several regional taxonomic reviews have been carried out [20,21,22,24], the taxonomy of squatinids in the western Indian Ocean has remained poorly understood.

One of the largest collections of deep-water chondrichthyans occurring from the western Indian Ocean was made by the Russian R.V. ‘Vityaz’ in 1988 and 1989. This mostly deep-water survey sampled chondrichthyans from the Gulf of Aden to the southern end of the Madagascar Ridge at Walters Shoal and included several remote and poorly sampled localities such as around the Socotra Islands, the slope off Somalia and Kenya, the deep southern Mozambique Channel, the Madagascar Ridge, and the Saya de Malha Bank. A juvenile specimen of *Squatina africana* and three juveniles of a *Squatina* sp. were collected together with other deep-water sharks, skates and rays, and chimaeras, many of which proved new to science. Matthias F.W. Stehmann, who collected the material, noted the unusual catch location of the latter three *Squatina* specimens and that they appeared different from typical specimens of *S. africana* in their morphology. Nevertheless, investigations of angel sharks from the southwestern Indian Ocean by S.W. showed that *S. africana* apparently exhibits a strong variation in general coloration and patterning. Furthermore, due to the known intraspecific variability and ontogenetic changes in angel sharks, the availability of adult specimens appeared crucial for a definite decision and for providing a comprehensive description of the new species. Therefore, the confirmation of the undescribed species was preceded by an intensive global search for adult specimens, but despite the recognition of further juvenile specimens in other museum collections, no adults were found in any collection. Finally, adult specimens were caught and retained off the coast of India and by Thai Trawlers operating in the central western Indian Ocean. This new material—in combination with the examination of the type and new material of its closest congener, *S. africana*—finally enabled us to describe the new species rigorously based on morphological, morphometric, and meristic, as well as molecular data.

The present paper represents contribution no. 23 to the series “Deep-water chondrichthyan fishes of R.V. ‘Vityaz’ cruise 17 and other Soviet cruises in the Indian Ocean”, initiated with the description of *Rhinochimaera africana* [25]. A key to the species of *Squatina* in the Indian Ocean is provided.

## 2. Materials and Methods

Institutional acronyms follow Sabaj [26] except for SW = Simon Weigmann personal collection. External morphometric measurements were taken by a vernier caliper to one-tenth of a millimeter (mm) from the specimens preserved in 70% ethanol (except for those deposited in the Reference Collection of Phuket Marine Biological Center (PMBC), Thailand, which were measured after thawing). Measurements follow Compagno [17], Vooren and da Silva [18], Last and White [22], and Vaz and Carvalho [24]. In addition, the mouth width was measured as opening only and as mouth width (outer jaws) including the jaw corners, and the skeletal interorbital width (INOS) was taken following Weigmann et al. [27,28]. As the caudal fin exhibits a low anterior ventral ridge in juveniles of both *S. africana* and the new species, additional measurements, in which the origin was set anteriorly including the low anterior fin ridge, were taken for those morphometrics involving the ventral origin of the caudal fin following Kaschner et al. [29], Weigmann et al. [27,30,31], and Weigmann and Kaschner [32]. The respective morphometrics are the ventral pre-caudal length, the distance from pelvic-fin origin to ventral caudal-fin origin, the distance from pelvic-fin insertion to ventral caudal-fin origin, and the length of the preventral caudal-fin margin. The data set of morphological measurements obtained was used to run a principal component analysis (PCA) in RStudio^©^ 4.2.0 (Posit, Boston, MA, USA) using the package “factoextra”. Measurements were standardized as % of TL [33]. Measurements not available for all specimens were removed, such as the measurements of the clasper [34].

The terminology for skeletal characteristics largely follows Vaz and Carvalho [24], that of the pectoral skeleton partially follows da Silva and Carvalho [35], and that of the pelvic skeleton partially follows da Silva and Vaz [36]; abbreviation cr = cranial roof follows Compagno [37]; abbreviations bb = basibranchials, ldf = lateral diazonal foramen, pcc = precerebral cavity, and vpp = ventral postorbital process are newly introduced. The terminology for clasper cartilages follows Vaz and Carvalho [14] and Moreira and Carvalho [11]. Illustrations of the pectoral fin and clasper were rendered using Adobe Illustrator^©^ 2023. Skeletal images from the CT-scan were rendered using Amira 5.3^©^ (Thermo Fisher, Waltham, MA, USA).

The map with catch locations of the examined specimens of both *Squatina* species was generated based on the Global Relief Model ETOPO1 by NOAA, the National Oceanic and Atmospheric Administration [38]. Country borders, lakes, and rivers were visualized by means of the shapefiles supplied by ESRI for the ArcExplorer-Java Edition for Education 2.3.2 (AEJEE) (ESRI, Redlands, CA, USA). For a map with all stations of cruise 17 of R.V. ‘Vityaz’, see Weigmann et al. [39,40].

Molecular data: two sources of sequence data were assembled and used in this study, a set of 14 previously published CO1 fragments for the 14 paratypes of *Squatina leae* sp. nov. deposited at PMBC and sequences generated in GN’s molecular lab as part of the ongoing “Chondrichthyan Tree of Life” project. CO1 sequences were assembled for 19 *Squatina* species including *S. leae* sp. nov. These were combined with the 14 pre-existing CO1 sequences for the 14 paratypes of *S. leae* sp. nov. at PMBC, previously published by S.W. and colleagues and deposited in GenBank (https://www.ncbi.nlm.nih.gov/genbank/ (accessed on 1 June 2023)) under accession numbers MW680886–MW680899 [41]. Fifty-one NADH2 sequences representing 19 different *Squatina* species were also assembled, aligned, and analyzed for the current study, including sequences for the holotype and one paratype of the new species. The CO1 and NADH2 data sets were analyzed separately and subjected to independent Maximum Likelihood analyses using a General Time Reversible model.

Nomenclatural Acts: the electronic edition of this article conforms to the requirements of the amended International Code of Zoological Nomenclature, and hence the new names contained herein are available under that Code from the electronic edition of this article. This published work and the nomenclatural acts it contains have been registered in ZooBank, the online registration system for the ICZN. The ZooBank LSIDs (Life Science Identifiers) can be resolved and the associated information viewed through any standard web browser by appending the LSID to the prefix “https://zoobank.org/” (accessed on 1 June 2023). The LSID for this publication is urn:lsid:zoobank.org:pub:CA98F7E5-AC12-4A0B-BCD5-41BEC325229A. The electronic edition of this work was published in a journal with an ISSN, and has been archived and is available from the following digital repositories: PubMed Central, LOCKSS.

## 3. Results

**Systematic account**

Family Squatinidae Bonaparte, 1838

Genus *Squatina* Duméril, 1806

### 3.1. Squatina leae sp. nov.

The species is registered in ZooBank under urn:lsid:zoobank.org:act:7E434980-B6B3-45B1-AAE9-3DE322B43CC6.

English name: Lea’s angel shark

Spanish name: Angelote de Lea

German name: Leas Engelhai

Figures 1–10, 18, 19, and 21–34; Table 1 and Table 2

*Squatina africana*―Gubanov et al. 1986: 218 (in part) [42]; ?Baissac 1990: 2 [43]; Gubanov 1993: 215–217 (in part) [44]; ?Fricke 1999: 28 (based on checklists by Baissac [43,45]) [46]; ?Manilo and Bogorodsky 2003: S77, S93 (based on Gubanov [44]) [47]; ?Reeve et al. 2011: 7 [48]; Akhilesh et al. 2014: 115 [49]; ?Jawad 2018: 66–67 [50]; Ambily et al. 2018: 312–317 (in part) [51].

*Squatina* cf. *africana*—Krajangdara et al. 2021: 17–30 [41].

*Squatina* sp.—?Baissac 1976: 195 [45].

*Squatina squatina*—?Joshi et al. 2008: 104 [52].

The holotype is deposited in the Marine Biodiversity Museum, ICAR-Central Marine Fisheries Research Institute, Kochi [Cochin], India (ICAR-CMFRI); three paratypes are deposited in the Zoological Museum Hamburg (ZMH); one paratype each in the National Museum of Nature and Science, Zoology Department, Division of Fishes, Tsukuba, Japan (NSMT) and the South African Institute for Aquatic Biodiversity (SAIAB); and 14 paratypes in the Reference Collection of Phuket Marine Biological Center (PMBC), Thailand.

Holotype **CMFRI GA. 15.2.5.4**, 671 mm TL, adult male, off Lakshadweep, southwestern India, 11°5′47″ N, 72°2′21″ E, 100–500 m depth, 28 September 2016.

Paratypes (20) **ZMH 26097**, juvenile male, 298 mm TL fresh, 282.6 mm TL 70% ethanol preserved, Saya de Malha Bank, 11°45′ S, 60°56′ E–11°45′ S, 60°58′ E, 250–260 m depth, R.V. ‘Vityaz’, cruise 17, station 2795, 29 m shrimp trawl, trawl # 82, on the bottom from 5:30 to 6:10 p.m., 6 January 1989, collected by Matthias F.W. Stehmann; **ZMH 26098**, juvenile male, 259 mm TL fresh, 249.6 mm TL 70% ethanol preserved, same data as paratype ZMH 26097; **ZMH 26099**, juvenile female, 263 mm TL fresh, 250.2 mm TL 70% ethanol preserved, same data as paratype ZMH 26097; **PMBC 21219.01**, female, 562 mm TL, weight 1610 g, latitude 10°27′ N to 10°42′ S, longitude 61°35′ E to 60°45′ E, Thai fishing vessel using single boat bottom otter trawls, landed at Ranong fishing port (Thailand) on 23 February 2017; **PMBC 21219.02**, female, 537 mm TL, 1510 g, same data as paratype PMBC 21219.01; **PMBC 21219.03**, adult female, 800 mm TL, 4820 g, same data as paratype PMBC 21219.01; **PMBC 21219.04**, adult female, 780 mm TL, 5050 g, same data as paratype PMBC 21219.01; **PMBC 21219.05**, adult female, 756 mm TL, 4740 g, same data as paratype PMBC 21219.01; **PMBC 21219.06**, adult female, 734 mm TL, 3770 g, same data as paratype PMBC 21219.01; **PMBC 21219.07**, adult female, 718 mm TL, 3790 g, same data as paratype PMBC 21219.01; **PMBC 21219.08**, adult female, 713 mm TL, 3800 g, same data as paratype PMBC 21219.01; **PMBC 21219.09**, adult male, 691 mm TL, 2940 g, same data as paratype PMBC 21219.01; **PMBC 21219.10**, adult male, 685 mm TL, 3020 g, same data as paratype PMBC 21219.01; **PMBC 21219.11**, adult male, 679 mm TL, 2760 g, same data as paratype PMBC 21219.01; **PMBC 21219.12** (only jaws preserved), adult male, 674 mm TL, 2660 g, same data as paratype PMBC 21219.01; **PMBC 21219.13**, adult male, 650 mm TL, 2330 g, same data as paratype PMBC 21219.01; **PMBC 21219.14**, adult male, 645 mm TL, 2490 g, same data as paratype PMBC 21219.01; **NSMT-P 117595**, juvenile male, 300 mm TL, Mascarene Plateau, 11°34′ S, 61°19′ E, 206 m depth, R.V. ‘Shinkai-maru’, 30 August 1977 (photographs and radiograph); **SAIAB 84178**, juvenile female, 370 mm TL, with tissue sample and juvenile male, 373 mm TL, Mascarene Plateau, 10°54.75′ S, 60°58.17′ E, 127 m depth, 30 October 2008 (photographs, radiographs and tissue sample).

**Diagnosis.** A small angel shark species (maximum size 870 mm TL) with the following characteristics: dorsal coloration conspicuously bright, beige to light grayish-brown, with many light yellowish flecks on trunk, and pectoral and pelvic fins, as well as countless densely set, minute dark spots, partially forming pseudocelli, all over the dorsal surface; no median row of scute-like denticles on trunk; anterior nasal flap with two lateral, elongate barbels and a medial rectangular barbel, all with ventral margins slightly fringed to almost smooth; concave between eyes; posterior nasal flap with an additional barblet; pectoral-pelvic space 10.0–14.9% TL; pectoral-fin apex angular; pelvic-fin free rear tips not reaching level of first dorsal-fin origin; tail moderately long, its length from cloaca 50.2–58.5% TL; pectoral fins moderately long, length 31.1–35.2% TL; dorsal fins not lobe-like; first dorsal-fin base somewhat longer than second dorsal-fin base; caudal fin of adults with angular apices; monospondylous centra 43–46; diplospondylous precaudal centra 55–58; total precaudal centra 100–104; total vertebral centra 130–136; and pectoral-fin skeleton with propterygium articulating with four radials.

**Description of the holotype.** Values of the three ZMH and 14 PMBC paratypes in parentheses, more complex differences are described separately. Where relevant, ratios are based on horizontal measurements unless otherwise stated. Detailed morphometric measurements of the holotype, as well as the three ZMH and 14 PMBC paratypes, are given in Table 1; meristic counts can be found in Table 2.

**Figure 1:**
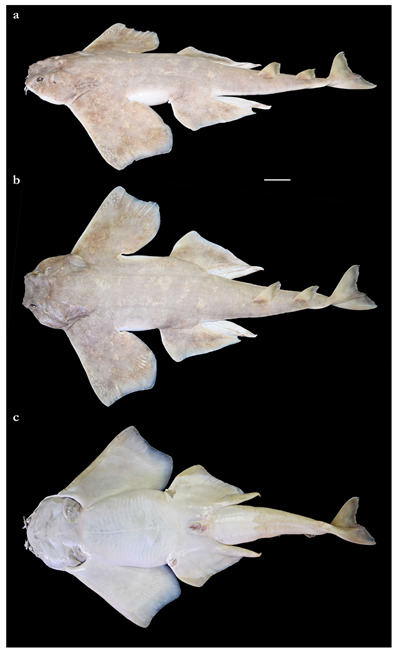
*Squatina leae* sp. nov., holotype, CMFRI GA. 15.2.5.4, adult male, 671 mm TL, in (**a**) dorsolateral, (**b**) dorsal, and (**c**) ventral views in fresh condition. Photographs kindly provided by P. U. Zacharia (ICAR-CMFRI). Scale bar: 5 cm.

**Figure 2:**
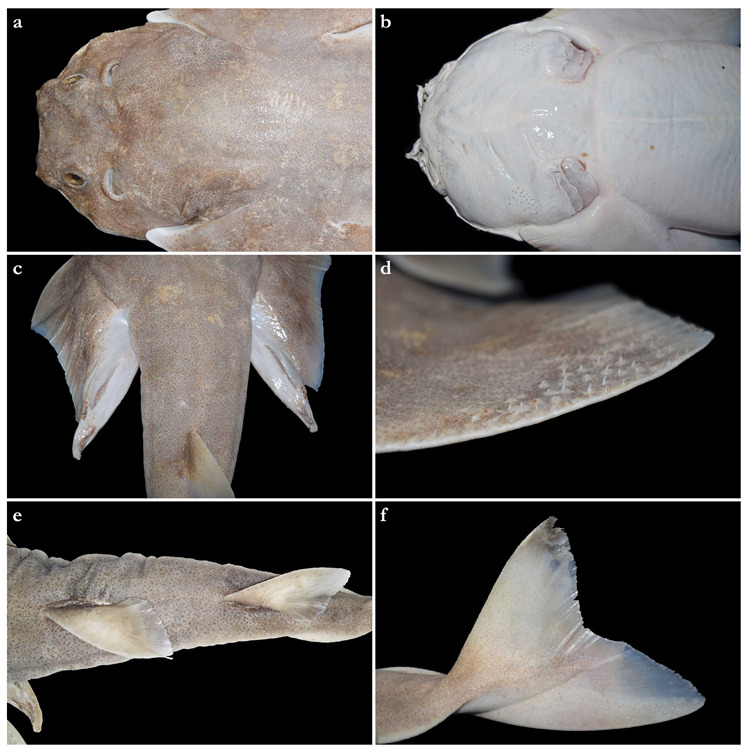
*Squatina leae* sp. nov., holotype, CMFRI GA. 15.2.5.4, adult male, 671 mm TL, head in (**a**) dorsal and (**b**) ventral views, (**c**) clasper region in dorsal view, (**d**) anterior pectoral-fin margin in dorsofrontal view, (**e**) dorsal fins in dorsal view, and (**f**) caudal fin in dorsolateral view. Photographs (**a**–**d**,**f**) kindly provided by P. U. Zacharia (ICAR-CMFRI) show the holotype in fresh condition, photograph (**e**) shows the holotype in preserved condition.

**Figure 3:**
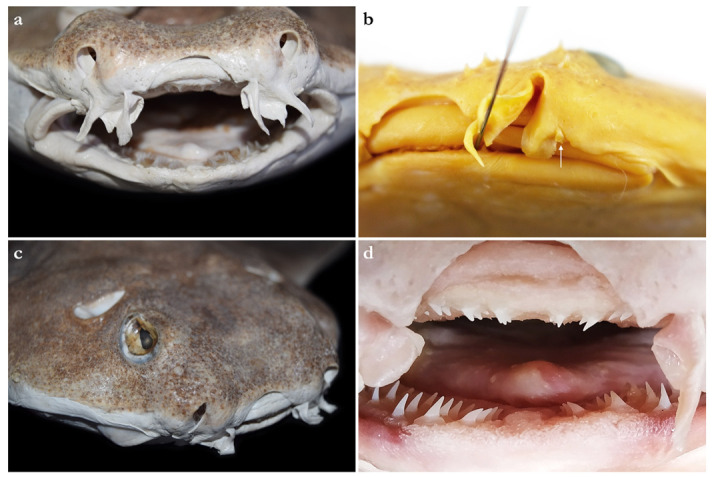
*Squatina leae* sp. nov., (**a**,**c**,**d**) holotype, CMFRI GA. 15.2.5.4, adult male, 671 mm TL, (**a**) anterior nasal flaps in frontal view, (**c**) dermal fold in dorsolateral view, and (**d**) tooth rows in frontal view; (**b**) paratype, ZMH 26097, juvenile male, 282.6 mm TL, posterior nasal flap with additional barblet (indicated by white arrow) in frontal view. Photographs (**a**,**c**) kindly provided by P. U. Zacharia (ICAR-CMFRI) show the holotype in fresh condition, photographs (**b**,**d**) show the respective specimens in preserved condition.

**Figure 4:**
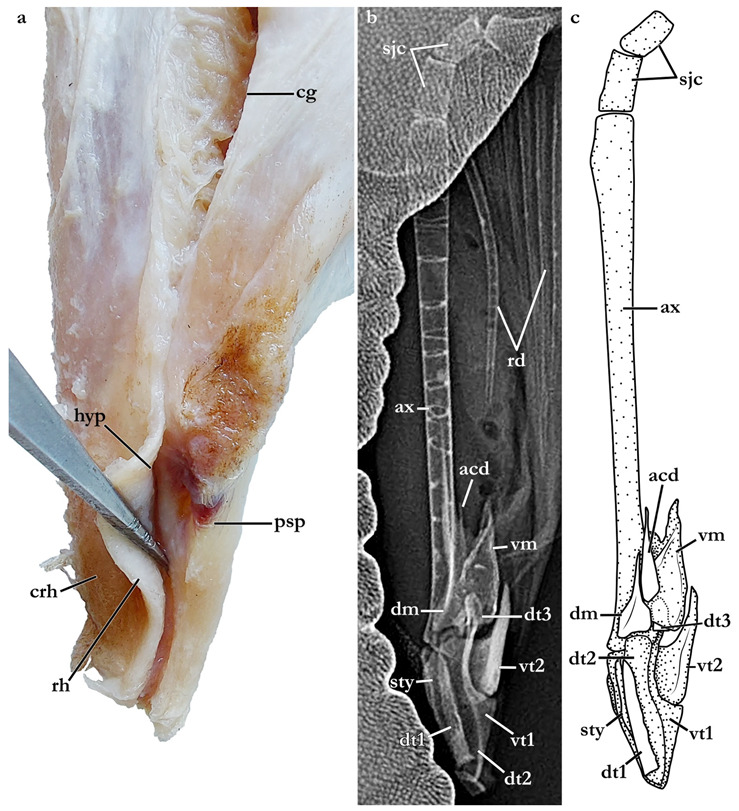
*Squatina leae* sp. nov., holotype, CMFRI GA. 15.2.5.4, adult male, 671 mm TL, clasper external morphology (**a**) and skeleton (**b**,**c**); (**a**) detail of left clasper glans with rhipidion spread to show morphological details of the spermatic duct (image reversed), (**b**) radiograph and (**c**) drawing of the right clasper skeleton. Dashed lines in (**c**) represent margins of dorsal terminal three cartilage engulfed by ventral marginal cartilage. The photograph (**a**) was taken and kindly provided by K. R. Aju and Sreekumar (CMFRI Museum). Abbreviations: ax, axial cartilage; acd, accessory dorsal marginal cartilage; cg, clasper groove; crh, cover rhipidion; dm, dorsal marginal; dt1, dorsal terminal 1 cartilage; dt2, dorsal terminal 2 cartilage; dt3, dorsal terminal 3 cartilage; sty, end-style cartilage; psp, pseudopera; rh, rhipidion; sjc, stem-joint cartilage; rd, pelvic-fin radials; vm, ventral marginal; vt1, ventral terminal 1 cartilage; vt2, ventral terminal 2 cartilage.

**Figure 5:**
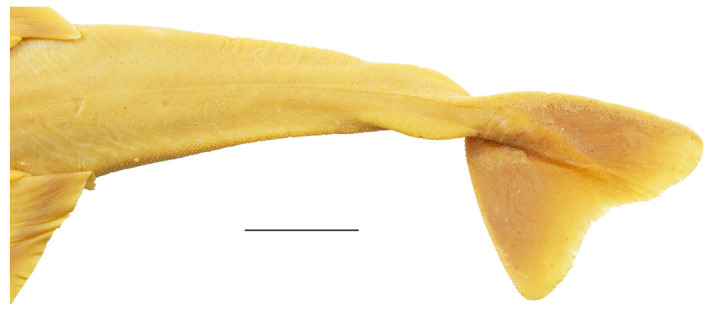
*Squatina leae* sp. nov., paratype, ZMH 26097, juvenile male, 282.6 mm TL, tail in ventral view showing ventral precaudal ridge. Scale bar: 2 cm.

**Figure 6:**
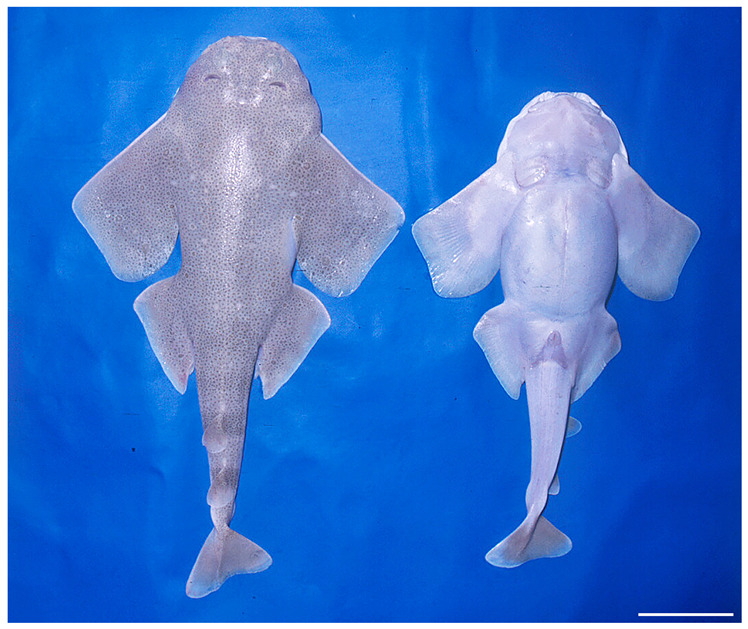
*Squatina leae* sp. nov., paratypes ZMH 26097, juvenile male, 298 mm TL fresh (in dorsal view) and ZMH 26098, juvenile male, 259 mm TL fresh (in ventral view) taken directly after catching. The photograph was taken and kindly provided by Matthias F. W. Stehmann. Scale bar: 5 cm.

**Figure 7:**
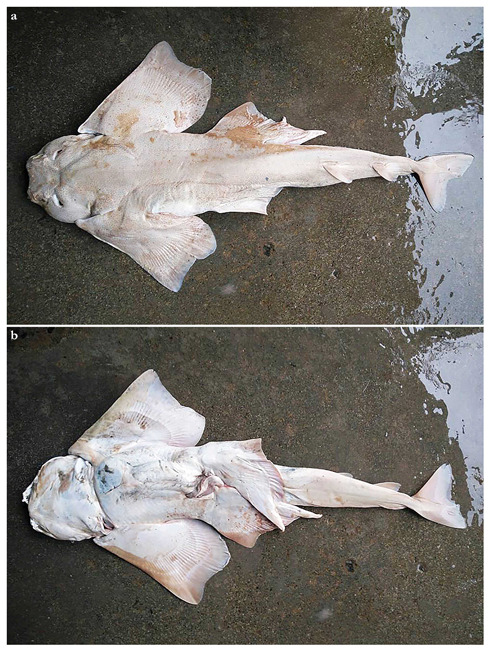
*Squatina leae* sp. nov., unretained male specimen caught off Laccadive Islands, Southwest India, June 2017, in (**a**) dorsal and (**b**) ventral views taken directly after landing in Kochi, India. The photographs were kindly provided by Mr. Basheer.

**Figure 8:**
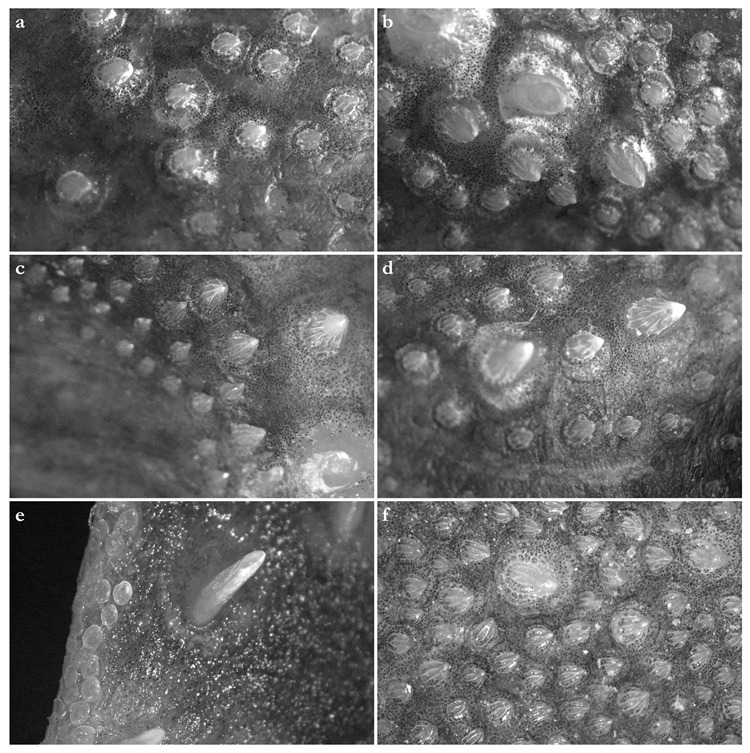
*Squatina leae* sp. nov., holotype, CMFRI GA. 15.2.5.4, adult male, 671 mm TL, microscopic images of dermal denticles in (**a**) interorbital, (**b**,**c**) postorbital, (**d**) post-spiracle, (**e**) anterior pectoral-fin, and (**f**) predorsal regions.

**Figure 9:**
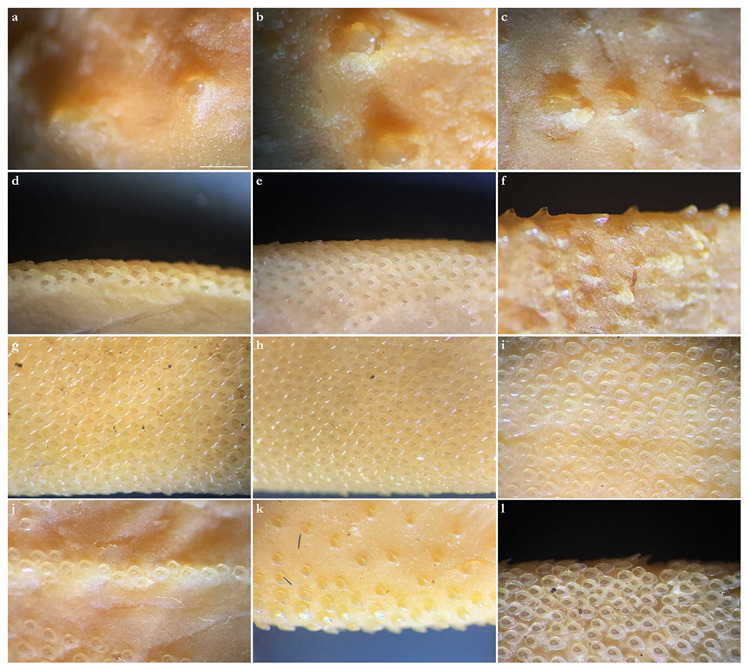
*Squatina leae* sp. nov., paratype, ZMH 26097, juvenile male, 282.6 mm TL, microscopic images of dermal denticles in (**a**) preorbital, (**b**) postorbital, (**c**) predorsal, (**d**) anterior pectoral-fin, (**e**) anterior pelvic-fin, (**f**) anterior dorsal caudal-fin, (**g**) ventral anterior pectoral-fin, (**h**) ventral anterior pelvic-fin, (**i**) ventral midtail, (**j**) ventral tail before origin of caudal-fin, (**k**) ventral lateral caudal keel, and (**l**) ventral caudal-fin regions. Scale bar (**a**–**l**): 1 mm.

*External morphology* (Figure 1, Figure 2, Figure 3, Figure 4, Figure 5, Figure 6, Figure 7, Figure 8 and Figure 9). Body stout, strongly depressed dorsoventrally, head height 41 (27–38)% head width at first gill slits. Trunk width 18.3 (18.9–25.1)% TL, tail width 0.6 (0.5–0.7) times width of trunk. Pectoral fins positioned on anterior quarter of body; pectoral insertion apart from pelvic origin by 10.9 (10.0–14.9)% TL (Figure 1). Head broad, its length about one-fifth of total length; largest width of head (similar at level of first gill slits and over spiracular region) also approximately one-fifth of total length. Anterior margin of head rounded to trapezoidal, projecting posteromedially on posterior quarter of head margins (Figure 2a,b). Dermal folds on lateral margin of head projecting from posterior edge of nostril to posterior end of head margin. Morphology of dermal folds variable, but all specimens with a wide and rounded lobe on anterior third of the fold, as well as partially a second, smaller lobe in posterior third (most obvious in largest ZMH paratype) (Figure 3c). ZMH female with the lobe on anterior third slightly subdivided. Snout short, its dorsal surface broadly flattened; preorbital distance 4.9 (2.1–6.0)% TL, without preorbital pit. Anterior margins between nostrils slightly convex to slightly concave. Eyes moderately small, elliptical, not protruding from head, eye diameter 2.8 (1.9–2.9)% TL, eye-width about two-thirds eye diameter. Eyes dorsally positioned on anterior quarter of head. Interorbital surface flat, integumental interorbital length 0.4 (0.4–0.5) times head (prepectoral) length. Spiracles separated from eyes by 2.3 (1.5–2.2)% TL, comma-shaped, and 1.0 (0.9–1.3) times eye diameter. Interspiracular surface straight; interspiracular length 1.0 (0.9–1.0) times integumental interorbital length, with a pair of pits positioned slightly posterior to a line connecting the inner ends of spiracles. These pits connect to the openings of the endo- and perilymphatic foramina through connective tissue. Pseudobranchial folds on anterior margin of spiracle ranging from 10 to 13 (number variable due to preservation).

Nostrils small, approximately one-half of eye diameter, terminal on head, internarial distance 6.7 (6.1–7.6)% TL. Anterior nasal flaps prominent, projecting ventrally to the lower lip of mouth. Anterior flap with two lateral, elongate barbels and a medial rectangular barbel (Figure 3a). Height of medial barbel about two thirds of lateral barbels. Ventral margins of medial and lateral barbels slightly fringed to almost smooth (the latter particularly in some PMBC paratypes). Posterior nasal flap an elongate and slightly fringed barbel almost reaching the lower lip on the posterior edge of nostrils, confluent with the origin of lateral dermal folds (Figure 3b). At the connection between the main barbel of the posterior nasal flap and the lateral dermal fold, there is a small barblet with a narrow, cylindrical base and a wide (1.5 wider than its base), flat, fringed ventral margin (Figure 3b). Mouth large, terminal on head, projecting to posterior level of eyes; mouth width about two-thirds of head width in spiracular region. Upper labial furrows conjoined, forming a deep preoral groove (as described in Vaz and Carvalho [24]). Lower labial furrows relatively small, about one-third of mouth width. Branchial apertures laterally covered by anterior projection of pectoral fin, but with exposed ventral edges. Branchial region short, distance between first and fifth gill slits 2.3 (1.9–2.9)% TL, but relatively wide, interbranchial width 7.5 (8.0–9.6)% TL.

Pectoral fins trapezoidal, large; pectoral-fin length approximately one-third of TL and 1.6 (1.5–2.1) times pectoral-fin width (Figure 1). Proximal portion of pectoral fins projected anteriorly, concealing the gill apertures laterally. Pectoral-fin anterior margin straight to slightly convex, 0.86 (0.8–0.91) times pectoral-fin length. Posterior margin concave, about one-half to two-thirds anterior margin length. Inner margin strongly convex, almost semi-circular, its length about equal to pectoral-fin width, inner margin length 0.9 (0.8–1.1) times pectoral-fin width. Base of pectoral fin large and robust, pectoral-fin base length 9.9 (10.3–12.6)% TL. Distal tip of pectoral fin not or only slightly overlapping pelvic fin and clearly not reaching pelvic-fin outer edge. Pelvic fins moderately large, triangular, pelvic-fin length 24.4 (21.7–25.7)% TL and 1.4 (1.6–2.3) times pelvic-fin width. Pelvic-fin anterior margin convex, posterior margin slightly concave, with an acute (acute or bluntly rounded) free rear tip. Pelvic-fin inner margin straight, 0.5 (0.41–0.57) times pelvic-fin length.

Clasper elongate and flattened. Clasper glans extending beyond the tip of pelvic fins when fully developed (Figure 2c). In adults, clasper inner length 19.5 (18.4–20.4)% TL, clasper outer length (measured in holotype only) 6.1% TL, clasper base width 3.4 (2.7–3.0)% TL, clasper outer length 0.31 times clasper inner length, and clasper base width 0.17 (0.13–0.16) times clasper inner length. Clasper groove long, approximately two-thirds of clasper length, extending from the apopyle to the distal third of the clasper. Cover rhipidion formed by thin layer of skin extending dorsomedially throughout clasper glans. Rhipidion elongate, slender, its greatest width being less than one-fourth of the width of clasper glans, projecting laterally from base of cover rhipidion. Rhipidion covering the hypopyle, pseudopera, and the floor of the glans. External wall of clasper glans weakly developed, as deep as the lateral margin of the rhipidion (Figure 4).

Tail elongate, longer than body (longer than body also in adult paratypes, subequal in length in juvenile paratypes), length from cloaca to tail tip 53.5 (50.2–51.0 in juvenile paratypes, 53.6–58.5 in adult paratypes)% TL and 1.2 (1.0 in juvenile paratypes, 1.2–1.3 in adult paratypes) times body length from snout tip to cloaca, ventrally depressed; tail width at pelvic-fin insertions 11.1 (10.2–14.8)% TL, tail height 0.5 (0.3–0.6) times tail width. Caudal peduncle strongly depressed, short, caudal peduncle length (dorsal caudal space) subequal to interdorsal distance, without precaudal pit and with discernible lateral caudal keels (Figure 1). Dorsal fins trapezoidal, similar in size and shape, positioned distinctly posterior to level of pelvic-fin free rear tips (Figure 2e). First dorsal-fin origin positioned on the posterior third of body, second dorsal on the posterior quarter. Anterior margins of dorsal fins slightly convex, with broadly angular outer apices (broadly angular also in other adults, more rounded in juveniles). Posterior margins straight, vertically oriented (convex in juvenile paratypes); inner edges rounded. Inner margins slightly convex. Caudal fin hypocercal, upper lobe margin slightly convex, 13.5 (12.0–14.0)% TL; lower lobe also with slightly convex margin, longer than upper lobe by 1.4 (1.2–1.4) times (Figure 2f). Posterior margin of caudal fin with a large notch at its medial depth, delimiting upper and lower posterior margins. The caudal fin of the holotype and all large paratypes does not have a clear preceding, low anterior ventral ridge; however, such a ridge is detectable in the small juvenile paratypes at ZMH (Figure 5).

***Coloration in fresh specimens***—Dorsal coloration conspicuously bright, beige to light grayish-brown, with many light yellowish flecks on trunk, and pectoral and pelvic fins, as well as countless densely set, minute dark spots, partially forming pseudocelli, all over the dorsal surface (Figure 6 and Figure 7). All fins with somewhat lighter narrow anterior and strikingly white posterior margins, particularly in juveniles (Figure 6). Clasper dorsally predominantly creamy-white, only distal part posterior to pelvic-fin free rear tips light grayish-brown and with densely set, minute dark spots (Figure 7). Juveniles with about three indistinct darker bands on tail (Figure 6). Ventral surface whitish (Figure 6 and Figure 7).

***Dentition***—Small gradient monognathic heterodonty following Compagno [37]. Upper jaw teeth smaller than lower jaw teeth. Upper teeth from fourth to seventh rows approximately 1.5 times larger than those on the first three medial upper vertical rows. On lower jaw, teeth of medial and central vertical rows similar in size. In both upper and lower jaws, teeth of distalmost rows approximately one-half size of teeth on central rows. Upper jaw with 18 (18–20) and lower jaw with 18 (18–19) tooth rows; tooth formula: 9 + 9 (9 + 9 to 10 + 10)/9 + 9 (9 + 9 to 9 + 10). Tooth rows arranged without lateral overlapping (independent dentition of Strasburg [53]). Tooth morphology similar to other species of *Squatina*, with bulky triangular main cusp slanting lingually, without serrations and accessory cusplets. Crown base laterally expanded with prominent lateral heels (Figure 3d).

***Dermal denticles***—*Dermal denticles in adult male holotype (Figure 8)*: dorsally, skin almost entirely covered by dermal denticles, except on posterior thirds of dorsal, pelvic and caudal fins, anterior apices of pectoral fins, and dorsal surfaces of claspers. Ventrally, skin mostly devoid of denticles except for anterior margin of pectoral fins, outer edges of pelvic fins, and posterior two thirds of tail, starting in a W-shaped manner somewhat before clasper tips. Dermal denticles compactly arranged; dorsally, dermal denticles on midline of trunk only slightly enlarged compared to other body denticles. Ventrally, dermal denticles similar in size throughout. Dorsal dermal denticles with a pyramidal-shaped crown projecting posteriorly. Most denticles with four ridges, extending from base to crown apex, but small denticles on outer pectoral-fin margins leaf-shaped without ridges; strongly enlarged denticles on pectoral-fin outer edges conical with needle-shaped crowns. Crown morphology of slightly enlarged denticles on dorsal midline similar to other trunk dermal denticles; no enlarged denticles along dorsal midline of tail. Basal plate of dermal denticles mostly rounded, but elliptical in the strongly enlarged needle-shaped pectoral-fin denticles. Clusters of conical and enlarged dermal denticles symmetrically arranged on dorsal region of head. A pair of denticle clusters positioned between nostrils (on margins of anterior fontanelle); two pairs of clusters on each orbital region: one on preorbital surface (over supraorbital crest) and another positioned posterior to eyes, atop postorbital processes; a pair of clusters of enlarged denticles present between spiracles. Basal plate of conical and enlarged dermal denticles on dorsal head rounded, about three times the diameter of smaller dermal denticles on trunk; conical crown of enlarged denticles on dorsal head with a smooth base, mostly with numerous (>10) vertical ridges extending to mid-height of crown, and with an acute and smooth apex. Sexual dimorphism present in squamation: outer edge of pectoral fins of adult males with a cluster of strongly enlarged conical dermal denticles with needle-shaped crowns, spaced apart from each other, with thick skin covering most of basal plate. Conical crown of these pectoral-fin denticles distinctly larger than that of any other denticles and entirely smooth, without any vertical ridges.

*Dermal denticles in juvenile ZMH paratypes (Figure 9)*: dorsal squamation: body almost entirely covered with loosely-set dermal denticles; pectoral and pelvic fins covered only but densely along anterior margins and in the area connecting the fins with the body; caudal fin covered only along anterior margins of both lobes and on central area around vertebral column; dorsal fins only covered in the area connecting the fins with the body and loosely along anterior margin (mainly first dorsal fin, second one only weakly and loosely set with denticles along anterior margins). Ventral squamation: trunk devoid of denticles; with respect to the fins, only anterior margins of pectoral and pelvic fins with a broad band of densely set denticles; tail densely set centrally and along outer margins, somewhat more loosely set in between, particularly in the two smaller specimens with stripes almost devoid of denticles; denticles start about 23 mm (female) or 27 mm (both males) behind posterior end of cloaca as two narrow stripes that broaden posteriorly to become the central (and almost complete) coverage of the tail; lateral keels on posterior tail also set with denticles, like also a stripe on lower anterior ventral caudal-fin ridge but again with smooth patches in between. In the smaller male, a narrow smooth stripe along the midline of the tail; in the female, a single row of denticles directly on the midline of tail, flanked by narrow smooth stripes; in the larger male, central area completely prickly except for the smooth anterior tail as described before and the smooth stripes that expand next to the prickly midline (equal to anterior lower caudal-fin ridge) about level with origin of lateral keels. Size of dermal denticles more or less similar along body except for somewhat enlarged ones in a midline from about level anterior end of pectoral base (equal to shoulder girdle level) to first dorsal-fin origin; median row of only very slightly enlarged denticles in interdorsal space: (1) In the largest male (ZMH 26097), about 70 enlarged denticles in a median row anterior to first dorsal-fin origin, with the denticles more enlarged in anterior (about the first 15 anteriormost denticles are more enlarged) and—particularly—posterior (about the posteriormost 20 denticles are more enlarged) areas but only very slightly enlarged in mid-area; about 10 very slightly enlarged denticles in interdorsal midline. (2) In the smaller male (ZMH 26098), about 75 denticles in a median row, again about the first 15 and last 20 ones more enlarged, whereas very small and hardly detectable in middle area. (3) In the female (ZMH 26099), denticles clearly more enlarged than in the two males but only about 50 denticles in the median row anterior to D1, and this row ends about 15 mm before D1 origin, whereas in the males, the median row ends right before D1 origin. As in the males, the denticles in mid-area are less enlarged but, contrary to males, the largest denticles are found in the anterior part of the median row and not in the posteriormost part, where the denticles are clearly smaller than the anteriormost ones of the median row; about 10 only slightly enlarged denticles in median interdorsal row. Dermal denticles in areas densely set with denticles with leaf-shaped crowns without ridges (Figure 9d,e,g–l), denticles in areas with loose denticle coverage, including dorsal midline, with pyramidal-shaped crowns with four ridges (Figure 9a–c,f). Clusters of pyramidal and enlarged dermal denticles symmetrically arranged on dorsal region of head. A pair of denticle clusters positioned between nostrils (on margins of anterior fontanelle); two pairs of clusters on each orbital region: one on preorbital surface (over supraorbital crest) and another positioned posteriorly to eyes, atop postorbital processes; a pair of single enlarged denticles present between spiracles.

***Size and maturity***—Largest adult male type specimen is 691 mm TL (PMBC 21219.09); smallest adult male is 645 mm TL (PMBC 21219.14). Among unretained specimens, a 717 mm TL adult male was present (pers. comm. Tassapon Krajangdara), representing the largest known male specimen of the new species. Largest juvenile male (with clearly immature claspers) is 373 mm TL (SAIAB 84178). Based on the examined specimens, the maturity size of males is less than 645 mm TL. Considering the small maximum size of males (717 mm TL), a maturity size well below 645 mm TL can be assumed.

Largest female paratype is 800 mm TL (PMBC 21219.03). Female paratypes of 713 (PMBC 21219.08), 718 (PMBC 21219.07), 734 (PMBC 21219.06), 756 (PMBC 21219.05), and 780 mm (PMBC 21219.04) mm TL are presumably adult due to their large size and heavy weight. Female paratypes of 537 (PMBC 21219.02) and 562 (PMBC 21219.01) mm TL are supposedly juvenile considering the size and weight differences. Small female paratypes of 263 (ZMH 26099) and 370 (SAIAB 84178) mm TL are clearly juvenile. Among unretained specimens, an 870 mm TL adult female was present (pers. comm. Tassapon Krajangdara), representing the largest known specimen of the new species.

The size at birth is about 180 to 190 mm TL based on two late-term embryos having been aborted when their mothers were caught (pers. comm. Tassapon Krajangdara).

***Meristics (n = 7)***—Total vertebral centra 130 (132–136), monospondylous centra 45 (43–46), diplospondylous precaudal centra 56 (55–58), total precaudal centra 101 (100–104), diplospondylous caudal centra 29 (31–33). Pectoral-fin radials: propterygium 4, mesopterygium 12, and metapterygium 21 (22–24). Pelvic-fin radials: 28 (24–26).

**Figure 10:**
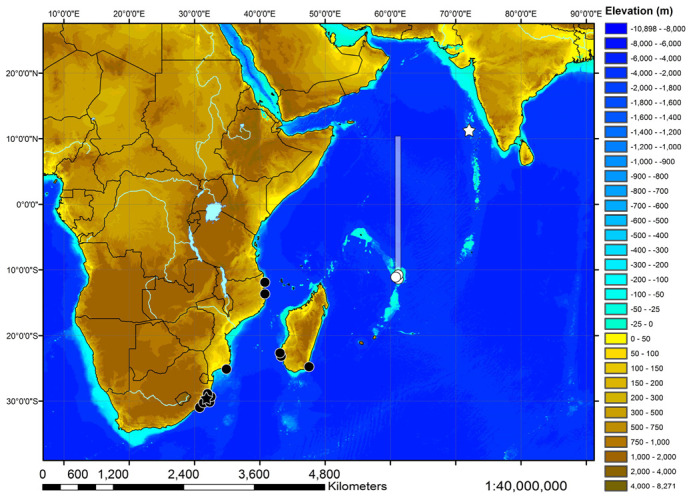
Map of the western Indian Ocean depicting the catch locations of the holotype (white star) and paratypes (white circles) of *Squatina leae* sp. nov., as well as the holotype (black star) and examined non-type specimens (black circles) of *S. africana*. The catch area of the 14 paratypes of *S. leae* sp. nov. at PMBC is indicated as a semitranslucent rectangle.

***Geographic distribution***—The new species is currently known from the western Indian Ocean on the Mascarene Plateau and off southwestern India in 100–500 m depths (Figure 10).

***Etymology***—The name is dedicated to the memory of Lea-Marie Cordt, the late sister of the first author’s fiancée.

***Remarks***—Angel sharks from off southern Africa have been well known and subject to several studies for quite some time. However, the presence of angel sharks in oceanic areas of the western Indian Ocean and, particularly, the northwestern Indian Ocean has been hardly known. In 2011, one of us (S.W.) briefly examined three pale-colored juvenile angel sharks, brought to his attention by Matthias F.W. Stehmann, who had collected them on the Saya de Malha Bank in 1989. Despite of the apparent differences to juvenile *Squatina africana* specimens from off South Africa and Mozambique, the lack of larger specimens hindered the description of the new species. It took more than five years until adult specimens could be found, caught by fishing vessels operating off southwestern India (holotype CMFRI GA. 15.2.5.4) and subsequently in the oceanic central western Indian Ocean (14 paratypes PMBC 21219.01–PMBC 21219.14). The former specimen was published as the first record of *S. africana* in Indian waters by Ambily et al. [51] and the latter 14 specimens—involving one of us (S.W.)—as *S.* cf. *africana* [41]. Although several adult males of the new species have been found and preserved in the type series, dissections of the clasper skeleton were not permitted, which is why the description of the claspers is restricted to external morphology and skeletal descriptions based on radiographs. In addition to the 14 paratypes at PMBC, 18 further specimens of the new species were landed at Ranong fishing port (Thailand) on 23 February 2017, of which only photographs are available. The overall 32 specimens included 10 adult males ranging from 650 to 717 mm TL and 2330 to 3020 g and 22 females ranging from 553 to 870 mm TL and 1510 to 6420 g. Further unretained specimens subsequently caught by Thai trawlers in the same area as the aforementioned specimens included one adult male (caught in 2021) and two late-term embryos of about 180 to 190 mm TL, which were aborted when the mother was caught (one from 2017 and one from 2021) (pers. comm. Tassapon Krajangdara). Another adult male was caught off Laccadive Islands, Southwest India, in June 2017 and landed in Kochi, India, but not retained (Figure 7). Joshi et al. [52] reported two specimens of *S. squatina* landed in Kochi between 2000 and 2002 but the length (1.0–1.2 m) and weight (22–28 kg) ranges are much too large for the new species.

The identification of the new species also has implications for the diagnosis of *S. africana*. Accordingly, the morphometric and meristic ranges for *S. africana* are updated in the following section, which provides a redescription of *S. africana*. This case demonstrates the importance of understanding intraspecific variation before proposing a new species. We reinforce that this may only be achieved with the investigation of large series of specimens in regional taxonomic reviews.

### 3.2. Squatina africana Regan, 1908

English name: African angel shark

Spanish name: Angelote africano

German name: Afrikanischer Engelhai

Figures 10–18, 20–25, 28, 30, 31, 33 and 34; Table 2 and Table 3

Holotype **BMNH 1906.11.19.21**, late subadult male, 837 mm TL, off Durban, South Africa, presented by E. Warren.

Non-types (13) **ZMH 25561**, juvenile male, 394 mm TL, Meteor 1964–1965 (International Indian Ocean Expedition [IIOE-1]), exact locality data unknown, collected by A. Kotthaus; **ZMH 26100**, juvenile male, 453 mm TL fresh, 446 mm TL 70% ethanol preserved, off southern Mozambique, 25°05′2″ S, 34°50′3″ E–25°05′ S, 34°44′2″ E, 90–92 m depth, R.V. ‘Vityaz’, cruise 17, station 2634, 19 m shrimp trawl, trawl # 31, on the bottom from 3:00 to 4:00 a.m., 25 November 1988, collected by Matthias F.W. Stehmann; **ZMH 123064** (ex ISH 32-1991), juvenile female, 309 mm TL, off Durban, South Africa, 29°47′ S, 31°23′ E, 350 m depth, R.V. ‘Africana’, station A4709-047-T03, bottom trawl, 26 August 1986, collected by Leonard J.V. Compagno; **ERB 0470** [field number 42], presumably adult female, 890 mm TL, anno 2002, off Natal, Natal Sharks Board (photographs); **ERB 0471** (SOB02015) [field number 121], gravid female, 880 mm TL, 6.5 kg, 26 March 2002, off Southbroom, Natal, Natal Sharks Board (photographs); **ERB 0968** (DUR 12073; RBINS P.25162), adult male, 810 mm TL, anno 2012, off Durban, Natal, Natal Sharks Board (photographs); **ERB 0971** (DUR 12074; RBINS P.25159), adult male, 820 mm TL, anno 2012, off Durban, Natal, Natal Sharks Board (photographs); all ERB specimens collected by Frederik H. Mollen (protocol by Mollen, 2019); **MNHN-IC-1987-1265**, juvenile male, 503 mm TL, off Toliara, Madagascar, 23°19′58.8″ S, 43°31′1.2″ E, 320 m depth, December 1985 (photographs and radiographs); **MNHN-IC-1988-0359**, juvenile female, 385 mm TL, off Fort-Dauphin, Madagascar, 25°2′6″ S, 47°5′6″ E, 65–70 m depth, 2 March 1973 (photographs and radiographs); **MNHN-IC-2003-0378**, juvenile male, 315 mm TL, off Toliara, Madagascar, 23°12′7.2″ S, 43°32′6″ E, 150 m depth, 31 March 1969 (photographs and radiographs); **SAIAB 187381**, adult male, 840 mm TL and possibly adult female, 940 mm TL, off northern Mozambique, 11.890694° S, 40.682769°E, R.V. ‘Fridtjof Nansen’, August 2009 (photographs and radiographs), **ZMMU P-14844**, juvenile female, 293 mm TL, off northern Mozambique, 13°52′5 S, 40°44′3 E, 186 m depth, R.V. ‘Professor Mesyatsev’, cr. 5, trawl # 31, 14 January 1976 (photographs).

**Diagnosis.** A medium-sized angel shark species (maximum size 1220 or possibly 1300 mm TL) with the following characteristics: dorsal coloration medium to dark brown, reddish-brown or grayish, with a variable pattern of numerous light and dark reddish spots and blotches, marbled with brownish reticulations and partially forming symmetrical dark bands or saddles; no median row of scute-like denticles on trunk; anterior nasal flap with two lateral, elongate barbels and a medial rectangular barbel, ventral margin of medial barbel slightly fringed but that of lateral barbels almost smooth; posterior nasal flap without additional barblet; concave between eyes; pectoral-pelvic space 6.0–10.2% TL; pectoral-fin apex angular; pelvic-fin free rear tips not reaching level of first dorsal-fin origin; tail moderately long, its length from cloaca 51.2–53.2% TL; pectoral fins moderately long, length 31.8–35.6% TL; dorsal fins not lobe-like; first dorsal-fin base somewhat longer than second dorsal-fin base; caudal fin of adults with angular apices; monospondylous centra 46–49; diplosondylous precaudal centra 58–62; total precaudal centra 104–111; total vertebral centra 134–143; pectoral-fin skeleton with propterygium articulating with three radials.

**Description.** The description is based on the holotype BMNH 1906.11.19.21, as well as the three non-type specimens ZMH 25561, ZMH 26100, and ZMH 123064. Where relevant, ratios are based on horizontal measurements unless otherwise stated. Detailed morphometric measurements and meristics are given in Table 3, and meristic counts can be found in Table 2.

**Figure 11:**
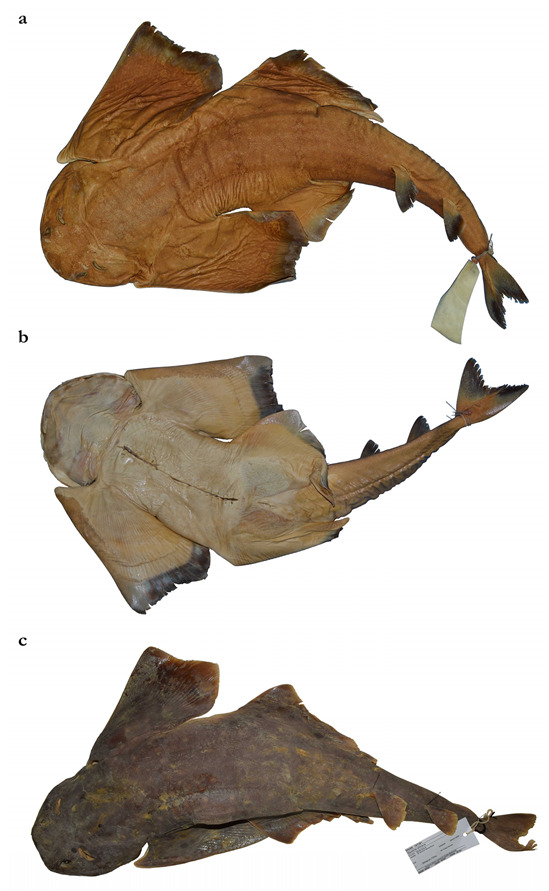
*Squatina africana*, (**a**,**b**) holotype, BMNH 1906.11.19.21, late subadult male, 837 mm TL, in (**a**) dorsal and (**b**) ventral views, as well as (**c**) non-type specimen, SAIAB 187381, adult male, 840 mm TL, in dorsal view. The photograph (**c**) was taken and kindly provided by Nkosinathi Mazungula (NRF-SAIAB).

**Figure 12:**
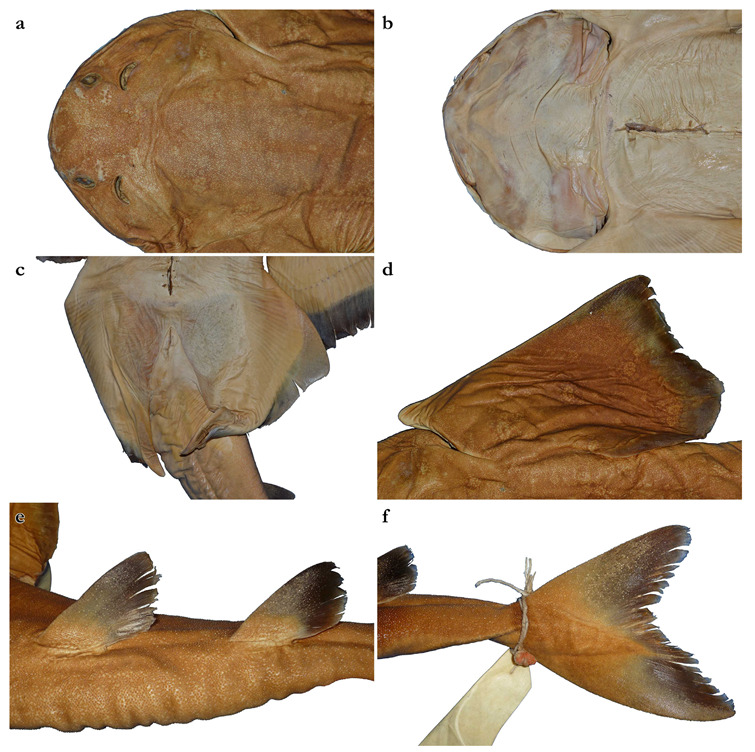
*Squatina africana*, holotype, BMNH 1906.11.19.21, late subadult male, 837 mm TL, head in (**a**) dorsal and (**b**) ventral views, (**c**) clasper region in ventral view, (**d**) right pectoral fin in dorsal view, (**e**) dorsal fins in dorsolateral view (image reversed), and (**f**) caudal fin in dorsolateral view.

**Figure 13:**
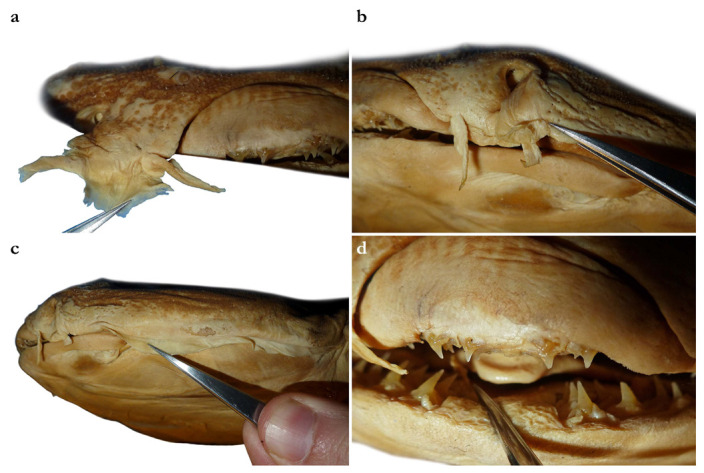
*Squatina africana*, holotype, BMNH 1906.11.19.21, late subadult male, 837 mm TL, (**a**) anterior nasal flaps in frontal view, (**b**) posterior nasal flap without additional barblet in frontal view, (**c**) dermal fold in lateral view, and (**d**) tooth rows in frontal view.

**Figure 14:**
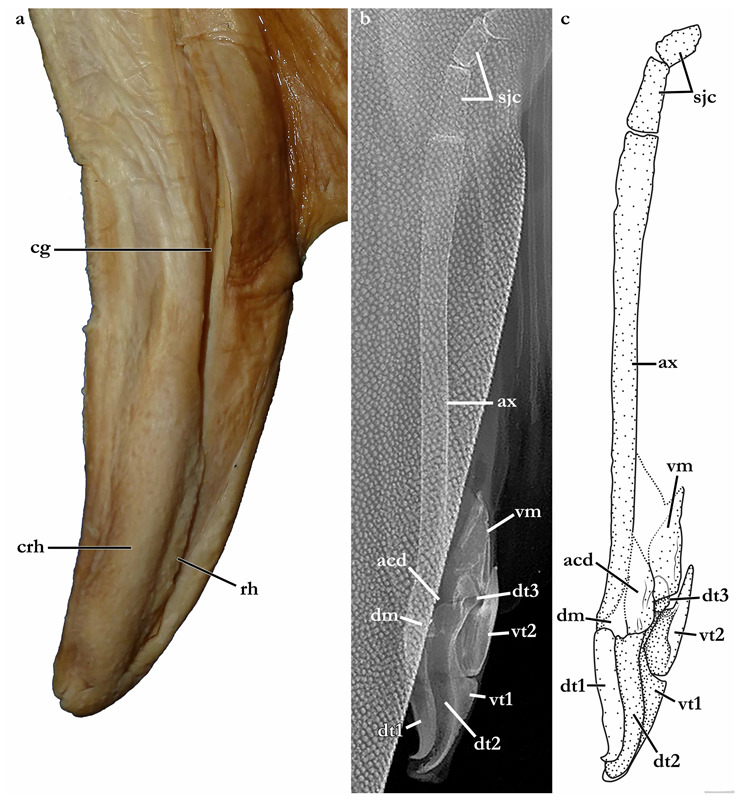
*Squatina africana*, (**a**) holotype, BMNH 1906.11.19.21, late subadult male, 837 mm TL, detail of right clasper glans to show external clasper morphology; (**b**,**c**) non-type specimen, SAIAB 187381, adult male, 840 mm TL, (**b**) radiograph and (**c**) drawing of the right clasper skeleton. Dashed lines in (**c**) represent margins of clasper cartilages that were not entirely visible in the radiograph. The radiograph (**b**) was taken and kindly provided by Nkosinathi Mazungula (NRF-SAIAB). Abbreviations: ax, axial cartilage; acd, accessory dorsal marginal cartilage; cg, clasper groove; crh, cover rhipidion; dm, dorsal marginal; dt1, dorsal terminal 1 cartilage; dt2, dorsal terminal 2 cartilage; dt3, dorsal terminal 3 cartilage; rh, rhipidion; sjc, stem-joint cartilage; rd, pelvic-fin radials; vm, ventral marginal; vt1, ventral terminal 1 cartilage; vt2, ventral terminal 2 cartilage.

**Figure 15:**
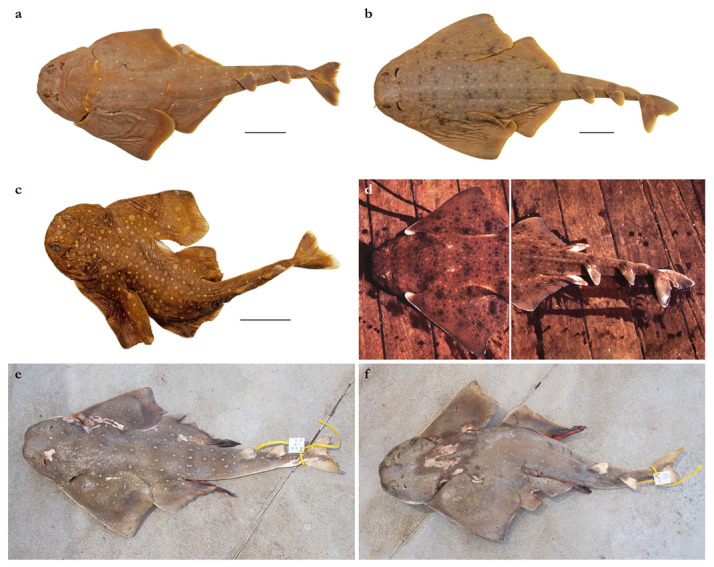
*Squatina africana*, variation in color pattern: (**a**) ZMH 25561, juvenile male, 394 mm TL, (**b**) ZMH 26100, juvenile male, 446 mm TL, (**c**) ZMH 123064, juvenile female, 309 mm TL, (**d**) uncatalogued adult male, (**e**) ERB 0968, adult male, 810 mm TL, (**f**) ERB 0971, adult male, 820 mm TL (image reversed). The fresh photograph (**d**) was taken by Phil Heemstra and kindly provided by Elaine Heemstra (NRF-SAIAB), the fresh photographs (**e**,**f**) were taken and kindly provided by Frederik Mollen (ERB). Scale bars: 5 cm.

**Figure 16:**
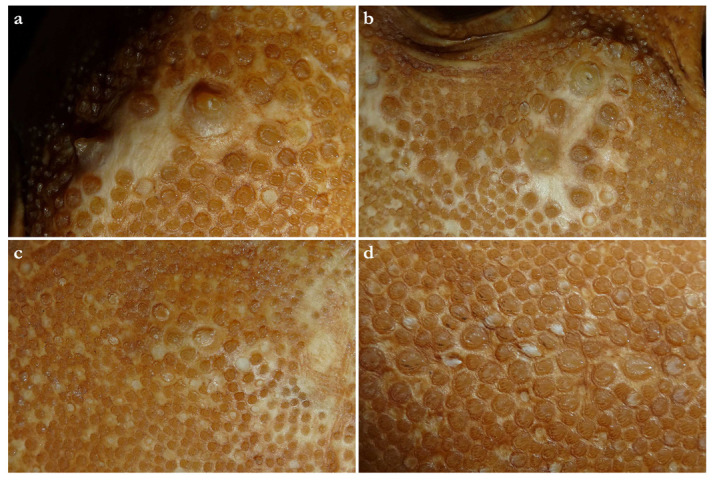
*Squatina africana*, holotype, BMNH 1906.11.19.21, late subadult male, 837 mm TL, dermal denticles in (**a**) right snout tip, (**b**) right postorbital, (**c**) right interspiracular, and (**d**) predorsal regions.

**Figure 17:**
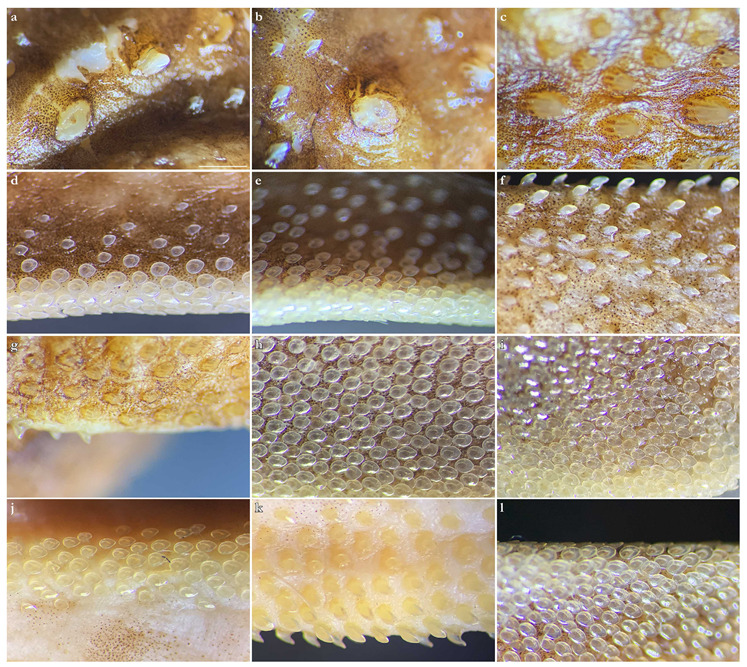
*Squatina africana*, non-type specimen, ZMH 123064, juvenile female, 309 mm TL, microscopic images of dermal denticles in (**a**) preorbital, (**b**) postorbital, (**c**) predorsal, (**d**) anterior pectoral-fin, (**e**) anterior pelvic-fin, (**f**) anterior dorsal caudal-fin, (**g**) anterior first-dorsal fin, (**h**) ventral anterior pectoral-fin, (**i**) ventral anterior pelvic-fin, (**j**) ventral tail before origin of caudal-fin, (**k**) ventral lateral caudal keel, and (**l**) ventral caudal-fin regions. Scale bar (**a**–**l**): 1 mm.

*External morphology* (Figure 11, Figure 12, Figure 13, Figure 14, Figure 15, Figure 16 and Figure 17). Body stout, strongly depressed dorsoventrally, head height 29–41% head width at first gill slits. Trunk width 16.1–17.9% TL, tail width 0.6–0.7 times width of trunk. Pectoral fins positioned on anterior quarter of body; pectoral insertion apart from pelvic origin by 6.0–10.2% TL (Figure 11). Head broad, its length about one-fifth of total length; largest width of head (similar at level of first gill slits and over spiracular region) also approximately one-fifth of total length. Anterior margin of head rounded, projecting posteromedially on posterior quarter of head margins (Figure 12a,b). Dermal folds on lateral margin of head projecting from posterior edge of nostril to posterior end of head margin but somewhat narrower than in the new species, particularly posterior to the rounded lobe in anterior third of the fold, which is present in all specimens (Figure 13c). Morphology of dermal folds appears to be constant with wide and rounded lobe on anterior third of the fold and narrowly pronounced rest of fold posteriorly. Snout short, its dorsal surface broadly flattened; preorbital distance 3.9–5.9% TL, without preorbital pit. Anterior margins between nostrils slightly convex. Eyes moderately small, elliptical, not protruding from head, eye diameter 1.9–3.1% TL, eye-width about two-thirds eye diameter. Eyes dorsally positioned on anterior quarter of head. Interorbital surface flat, integumental interorbital length 0.4–0.5 times head (prepectoral) length. Spiracles separated from eyes by 1.6–1.9% TL, comma-shaped, and 1.0–1.4 times eye diameter. Interspiracular surface straight; interspiracular length 0.9 times integumental interorbital length, with a pair of pits positioned slightly posterior to a line connecting the inner ends of spiracles. These pits connect to the openings of the endo- and perilymphatic foramina through connective tissue. Pseudobranchial folds on anterior margin of spiracle ranging from 11 to 14 (number variable due to preservation).

Nostrils small, approximately one-half of eye diameter, terminal on head, internarial distance 6.1–7.4% TL. Anterior nasal flaps prominent, projecting ventrally to the lower lip of mouth. Anterior flap with two lateral, elongate barbels and a medial rectangular barbel (Figure 13a). Height of medial barbel almost equal to that of lateral barbels and nearly reaching lower lip. Ventral margin of medial barbel slightly fringed but that of lateral barbels almost smooth. Posterior nasal flap broad, triangular, and slightly fringed about as long as medial rectangular anterior flap barbel and almost reaching the lower lip on the posterior edge of nostrils, confluent with the origin of lateral dermal folds; without additional barblet (Figure 13b). Mouth large, terminal on head, projecting to posterior level of eyes; mouth width about two-thirds of head width in spiracular region. Upper labial furrows conjoined, forming a deep preoral groove (as described in Vaz and Carvalho [24]). Lower labial furrows relatively small, about one-third of mouth width. Branchial apertures laterally covered by anterior projection of pectoral fin, but with exposed ventral edges. Branchial region short, distance between first and fifth gill slits 1.9–2.7% TL, but moderately wide, interbranchial width 7.0–8.7% TL.

Pectoral fins trapezoidal, large; pectoral-fin length approximately one-third of TL and 1.8–2.0 times pectoral-fin width (Figure 11). Proximal portion of pectoral fins projected anteriorly, concealing the gill apertures laterally. Pectoral-fin anterior margin straight to slightly convex, 0.82–0.87 times pectoral-fin length. Posterior margin concave, about two-thirds anterior margin length. Inner margin strongly convex, almost semi-circular, its length about equal to pectoral-fin width, inner margin length 0.9–1.0 times pectoral-fin width. Base of pectoral fin large and robust, pectoral-fin base length 10.3–13.1% TL. Distal tip of pectoral fin clearly overlapping pelvic fin but not reaching pelvic-fin outer edge. Pelvic fins moderately large, triangular, pelvic-fin length 22.2–23.7% TL and 1.8–2.1 times pelvic-fin width. Pelvic-fin anterior margin convex, posterior margin slightly concave, with an acute or bluntly rounded free rear tip. Pelvic-fin inner margin straight or slightly concave, 0.32–0.45 times pelvic-fin length.

Clasper elongate and flattened. Clasper glans extending beyond the tip of pelvic fins when fully developed (Figure 1). In the late subadult male holotype, clasper inner length 14.6% TL, clasper outer length 4.1% TL, clasper base width 1.6% TL, clasper outer length 0.28 times clasper inner length, and clasper base width 0.11 times clasper inner length. Clasper of the holotype with all sexual external features developed, but still soft, without full calcification. Clasper groove long, approximately two-thirds of clasper length, extending from the apopyle (located close to pelvic insertion) to the origin of the clasper glans, where it is positioned near the hypopyle (located in the distal third of the clasper). Cover rhipidion well developed (Figure 14), a bulbous layer of skin extending medially throughout clasper glans. Rhipidion elongate, thin, width less than one-sixth of the width of clasper glans, projecting laterally from base of cover rhipidion. Rhipidion covering the hypopyle, pseudopera, and the floor of the glans. External wall of clasper glans weakly developed, shorter than the depth of cover rhipidion (this feature, however, could be related to the incomplete maturation of the clasper, as this wall is supported by the dorsal terminal cartilage 2).

Tail elongate, longer than body at all maturity stages, length from cloaca to tail tip 51.2–53.2% TL and 1.1 times body length from snout tip to cloaca, ventrally depressed; tail width at pelvic-fin insertions 10.0–11.8% TL, tail height 0.5–0.6 times tail width. Caudal peduncle strongly depressed, short, caudal peduncle length (dorsal caudal space) subequal to interdorsal distance, without precaudal pit and with discernible lateral caudal keels (Figure 11). Dorsal fins trapezoidal, similar in size and shape, positioned distinctly posterior to level of pelvic-fin free rear tips (Figure 12e). First dorsal-fin origin positioned on the posterior third of body, second dorsal on the posterior quarter. Anterior margins of dorsal fins slightly convex, with broadly angular (holotype) to more rounded (juvenile non-types) outer apices. Posterior margins slightly convex; inner edges rounded. Inner margins slightly convex. Caudal fin hypocercal, upper lobe margin straight to slightly convex, 12.0–12.7% TL; lower lobe with slightly convex margin, longer than upper lobe by 1.4–1.6 times (Figure 12f). Posterior margin of caudal fin with a large notch at its medial depth, delimiting upper and lower posterior margins. The caudal fin of the holotype does not have a clear preceding, low anterior ventral ridge; however, such a ridge is detectable in all juvenile specimens.

***Coloration in fresh specimens***—Dorsal coloration medium to dark brown, reddish-brown or grayish, with a highly variable pattern of numerous light and, particularly, dark reddish spots and blotches, many of which are granular-centered ocelli or pseudocelli. The spots and blotches are marbled with brownish reticulations and the dark blotches partially form symmetrical dark bands or saddles, particularly in juveniles. All fins with narrow white margins, more pronounced in juveniles. Clasper dorsally predominantly dark or creamy-white; in the latter case, distal part posterior to pelvic-fin free rear tips brownish and marbled with dark flecks (Figure 15). Ventral surface whitish.

***Dentition***—Small gradient monognathic heterodonty following Compagno [37]. Upper jaw teeth smaller than lower jaw teeth. Upper teeth from fourth to seventh rows approximately 1.5 times larger than those on the first three medial upper vertical rows. On lower jaw, teeth of medial and central vertical rows similar in size. In both upper and lower jaws, teeth of distalmost rows approximately one-half size of teeth on central rows. Upper and lower jaws each with 18–20 tooth rows; tooth formula: 9 + 9 to 10 + 10/9 + 9 to 10 + 10. Tooth rows arranged without lateral overlapping (independent dentition of Strasburg [53]). Tooth morphology similar to other species of *Squatina*, with bulky triangular main cusp slanting lingually, without serrations and accessory cusplets. Crown base laterally expanded with prominent lateral heels (Figure 13d).

***Dermal denticles***—*Dermal denticles in late subadult male holotype (Figure 16)*: dorsally, skin almost entirely covered by dermal denticles, except on posterior thirds of dorsal, pelvic, and caudal fins, anterior apices of pectoral fins, and dorsal surfaces of claspers. Ventrally, skin mostly devoid of denticles except for anterior margin of pectoral fins, outer edges of pelvic fins and posterior two thirds of tail, starting in a W-shaped manner distinctly anterior to clasper tips. Dermal denticles compactly arranged; dorsally, dermal denticles on midline of trunk only very slightly enlarged compared to other body denticles. Ventrally, dermal denticles similar in size throughout. Dorsal dermal denticles with a pyramidal-shaped crown projecting posteriorly. Most denticles with four ridges, extending from base to crown apex, but small denticles on outer pectoral-fin margins leaf-shaped without ridges; strongly enlarged denticles on pectoral-fin outer edges possibly not yet developed or with crowns abraded but conical in the holotype, but with needle-shaped crowns in examined adult males. Crown morphology of very slightly enlarged denticles on dorsal midline similar to other trunk dermal denticles; no enlarged denticles along dorsal midline of tail. Basal plate of dermal denticles mostly rounded, but elliptical in the strongly enlarged needle-shaped pectoral-fin denticles. Clusters of conical and enlarged dermal denticles symmetrically arranged on dorsal region of head. A pair of denticle clusters positioned between nostrils (on margins of anterior fontanelle); two pairs of clusters on each orbital region: one on preorbital surface (over supraorbital crest) and another positioned posterior to eyes, atop postorbital processes; a pair of clusters of enlarged denticles present between spiracles. Many of the enlarged denticles have been abraded in the late subadult male holotype. Basal plate of conical and enlarged dermal denticles on dorsal head rounded, about three times the diameter of smaller dermal denticles on trunk; conical crown of enlarged denticles on dorsal head with a smooth base, mostly with more than four vertical ridges extending to mid-height of crown, and with an acute and smooth apex. Sexual dimorphism present in squamation: outer edge of pectoral fins of adult males with a cluster of strongly enlarged conical dermal denticles with needle-shaped crowns, spaced apart from each other, with thick skin covering most of basal plate. Conical crown of these pectoral-fin denticles distinctly larger than that of any other denticles and entirely smooth, without any vertical ridges. These strongly enlarged denticles are hardly detectable in the holotype, possibly due to the not full maturity of the specimen or due to having been abraded.

*Dermal denticles in juvenile ZMH specimens (Figure 17)*: dorsal squamation: body almost entirely covered with loosely-set dermal denticles; fins almost totally covered but densely only along anterior margins and in the area connecting the fins with the body, whereas the remaining parts are only very loosely set with minute denticles, clearly smaller than those on body and anterior fin margins; caudal fin very broadly covered along anterior margins. Ventral squamation: trunk devoid of denticles; with respect to the fins, only anterior margins of pectoral and pelvic fins with a broad band of densely set denticles (in pelvic fins, densely set denticles also on outer part of posterior margins); tail densely set with denticles throughout in specimens ZMH 25561 and ZMH 26100, but specimen ZMH 123064 with smooth areas next to the median row on anterior ventral caudal-fin ridge in the area of the lateral keels; denticles start about 18 (ZMH 123064), 35 (ZMH 25561), or 40 (ZMH 26100) mm behind posterior end of cloaca. Size of dermal denticles similar along body except for somewhat enlarged ones in a midline anterior to first dorsal-fin origin and, partially, interdorsally: (1) In juvenile male ZMH 26100, about 15 slightly enlarged denticles in a median row from about 60 mm anterior to directly before first dorsal-fin origin; no enlarged denticles interdorsally. (2) In juvenile male ZMH 25561, a more clearly pronounced row of about 23 enlarged denticles in posterior midline area from about 60 mm anterior to directly before first dorsal-fin origin; a row of roughly 15 only slightly enlarged denticles interdorsally. (3) In juvenile female ZMH 123064, denticles on dorsal trunk and tail generally appear proportionally larger than in the two larger specimens; a median row of about 20 slightly enlarged denticles from about 45 mm anterior to directly before D1 origin but, due to the generally larger size of the trunk denticles, the median row could also be interpreted as starting more anteriorly, roughly at pectoral-fin midbases, resulting in a median row of approximately 70 hardly enlarged denticles with mostly nearly identical size as other trunk denticles, except for about the 20 posteriormost ones, which are somewhat larger; interdorsally about 10 only slightly enlarged denticles. Dermal denticles in areas densely set with denticles with leaf-shaped crowns without ridges (Figure 9d,e,h–j,l), denticles in areas with rather loose denticle coverage, including dorsal midline, with pyramidal-shaped crowns with four ridges (Figure 9a–c,f,g,k). Clusters of pyramidal and enlarged dermal denticles symmetrically arranged on dorsal region of head. A pair of denticle clusters positioned between nostrils (on margins of anterior fontanelle); two pairs of clusters on each orbital region: one on preorbital surface (over supraorbital crest) and another positioned posteriorly to eyes, atop postorbital processes; an indistinct cluster of slightly enlarged interspiracular denticles only present in female ZMH 123064, whereas no enlarged ones are detectable in the juvenile males ZMH 26100 and ZMH 25561 (a slightly enlarged single interspiracular denticle might be present but only hardly pronounced in male ZMH 26100). In female ZMH 123064, additional enlarged denticles are present in orbital and anterior snout areas, forming a pattern together with the usual enlarged denticles. Such additional enlarged denticles are not present in any of the other specimens.

***Size and maturity***—The male holotype of 837 mm TL (BMNH 1906.11.19.21) is a late subadult specimen, while the 840 mm TL male from SAIAB 187381 is adult, like apparently also the 810 mm TL male ERB 0968 and the 820 mm TL male ERB 0971. Largest juvenile males (with clearly immature claspers) are 453 mm TL (ZMH 26100) and 503 mm TL (MNHN-IC-1987-1265).

Largest female examined is 940 mm TL (SAIAB 187381) and possibly adult. Small female specimens of 293 (ZMMU P-14844), 309 (ZMH 123064), and 385 (MNHN-IC-1988-0359) mm TL are clearly juvenile.

Based on the literature, *S. africana* is a medium-sized angel shark species reaching a maximum total length of 1220 mm [1,12] or possibly 1300 mm [42,44]. Sizes at birth and first maturity vary depending on the study: the size at birth is at least 240 mm TL according to Shelmerdine and Cliff [54], referring to Fennessy [55], between 280 and 300 mm TL following Ebert et al. [1], and between 280 and 340 mm TL after Bass et al. [56]. Maturity sizes range from 640–700 mm TL [54] and 750–770 mm TL [56] to 770–950 mm TL [1] in males and from 700 mm TL [54] and 900–930 mm TL [56] to 820–1070 mm TL [1] in females. Based on the material examined in the present study, the maturity size of males is about 810–840 mm TL.

***Meristics (n = 9)***—Total vertebral centra 134–143, monospondylous centra 46–49, diplospondylous precaudal centra 58–62, total precaudal centra 104–111, diplospondylous caudal centra 27–32. Pectoral-fin radials: propterygium 3, mesopterygium 12–13, and metapterygium 22–26. Pelvic-fin radials: 25–28.

***Geographic distribution***—*Squatina africana* is known only from the western Indian Ocean off southern and eastern Africa, as well as Madagascar. Examined material is from off South Africa, Mozambique, and Madagascar (Figure 10). Material from further north was not available for study and has not been found in any museum collection, but there have been reports of *S. africana* from off Kenya [57,58,59] and Tanzania [60], as well as less verifiable in the Gulf of Aden and off the Socotra Islands [44], off the Seychelles Islands [42,44], and off Mauritius [43,45]. If angel sharks are indeed found in these regions, it remains unclear if they belong to *S. africana* or the new species. The Natal Coast seems to be the center of distribution for *S. africana*, whereas it is apparently rare in the eastern Cape area [56]. The western limit of its distribution appears to be off Mossel Bay [61], and its known depth distribution is 0–600 m [12].

***Remarks***—The *Squatina africana* holotype BMNH 1906.11.19.21 was used as a reference for characterizing the true *S. africana*. It was caught off South Africa (off Durban), where the new species apparently does not occur. The identity of *Squatina* specimens occurring more northerly (off Mozambique) and more easterly (off Madagascar) and, thereby, the possible presence of the new species in this area were investigated by examination of specimens from the MNHN, SAIAB, and ZMMU collections. As all specimens examined were clearly identified as *S. africana* based on their morphology and meristics, it is assumed that *S. leae* sp. nov. does not occur off Mozambique or Madagascar and has a distribution allopatric to that of *S. africana*. However, eastern African *Squatina* material caught north of Mozambique was not available for examination. In the northwestern Indian Ocean, an overlap of both species appears possible.

Based on the material examined and available literature, *Squatina africana* exhibits a high degree of morphological variability. This is evidenced by the strong differences in the sizes at birth and at first maturity as indicated in the Size and maturity section. Interestingly, the studies cited apparently all refer to specimens caught in a small geographic region, i.e., off eastern South Africa. Morphological variability is particularly pronounced in the color patterning found in different specimens caught off South Africa and Mozambique. This includes variability in the dorsal ground color, as well as strong differences in the patterning of spots and blotches. The patterning ranges from few, isolated marks to dense coverage and also varies in the proportion of bright and dark spots and blotches, with some specimens exhibiting only few dark spots and/or blotches, others having a pronounced and dense coverage of dark spots and/or blotches, and some exhibiting dark blotches arranged in saddle-like markings (Figure 15).

### 3.3. Principal Component Analysis (PCA; Figure 18)

**Figure 18:**
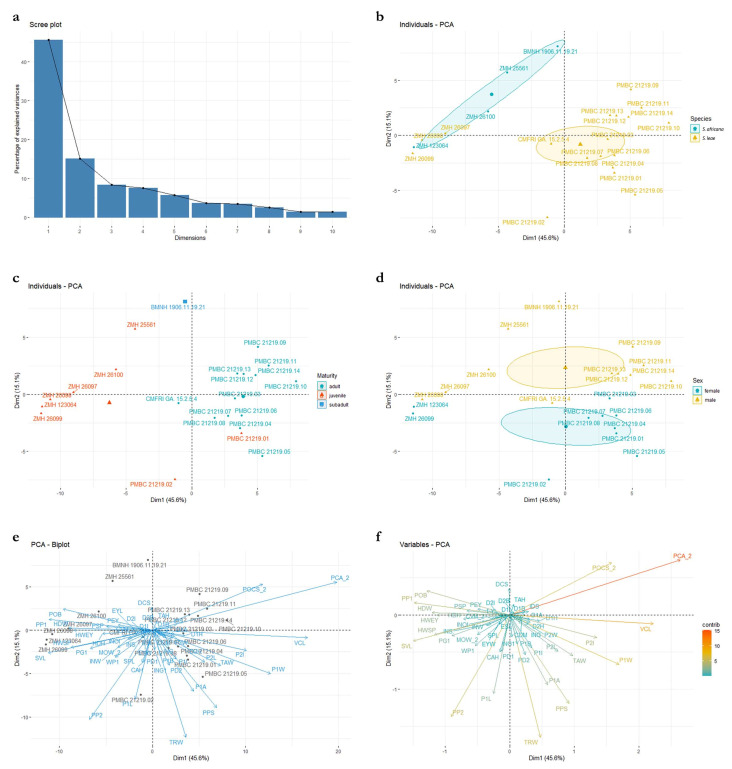
Principal component analyses using standardized measurements of all specimens of *Squatina leae* sp. nov. and *S. africana*. (**a**) Scree plot demonstrating the percentage of the variance explained by each PC axis. (**b**) Morphospace plotted on PC1 in the x axis, PC2 in the y axis, and specimens of each species assigned to a separate group. (**c**) Morphospace plotted on PC1 and PC2, assigning groups to different stages of maturity across both species. (**d**). Morphospace plotted on PC1 and PC2, assigning groups to different sexes in specimens of both species. (**e**) Morphospace plotted on PC1 and PC2 with labels of each individual specimen plotted over the values of the variance of each measurement. (**f**) Values of variance of each morphological measurement plotted on PC1 and PC2. Color gradient represents the percentage of the variance explained by each morphological measurement.

Principal components one and two capture most of the variance, explaining 45.6% and 15.1%, respectively (Figure 18a). The distribution of PC values in the morphospace finds a spatial separation between large juveniles (>500 mm TL) and adult specimens of *Squatina africana* (i.e., holotype) and *S. leae* sp. nov., but small juveniles of both species (<450 mm TL) are grouped together (Figure 18b). The visual separation between the confidence circles of *S. africana* and *S. leae* sp. nov., however, could potentially be artificial given the small number of specimens of *S. africana* that we had access to in this study. Inclusion of additional specimens, particularly females, could change the observed distribution of values of *S. africana* across the PC plot. The distribution of PC values combining both species shows a tenuous sexual dimorphism with male specimens mostly located on the positive area of the Dim2 (i.e., PC2), whereas PC values of female specimens have PC2 coordinates smaller than zero (Figure 18d), similar to the analysis by Krajangdara et al. [41].

### 3.4. Skeleton of Squatina leae sp. nov. and S. africana

**Figure 19:**
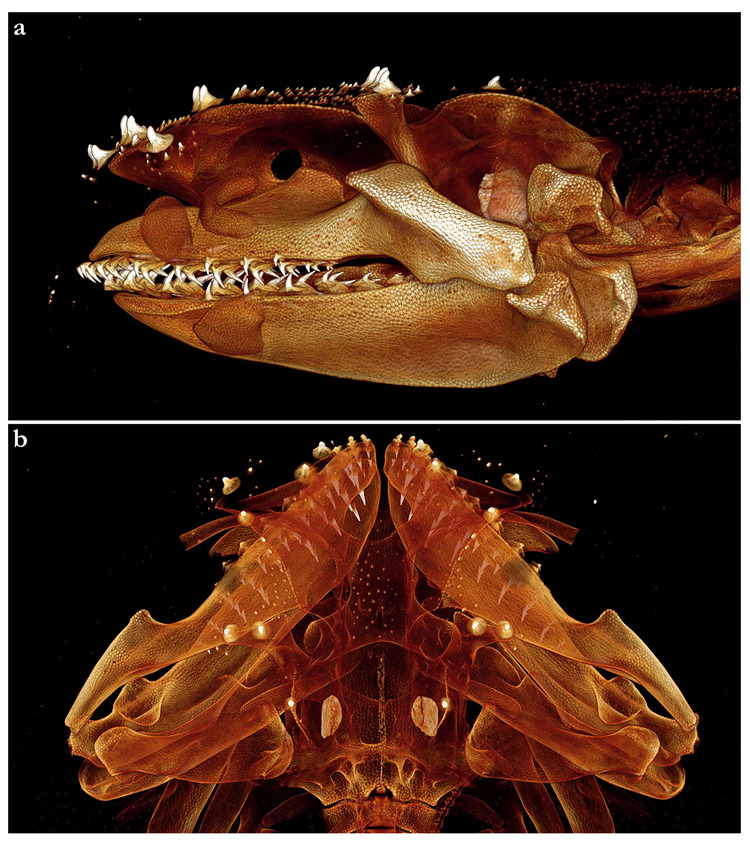
*Squatina leae* sp. nov., paratype, ZMH 26098, juvenile male, 249.6 mm TL; micro-computed tomography scans of the skull in (**a**) lateral and (**b**) dorsal views. Anterior to left in (**a**) and top in (**b**).

**Figure 20:**
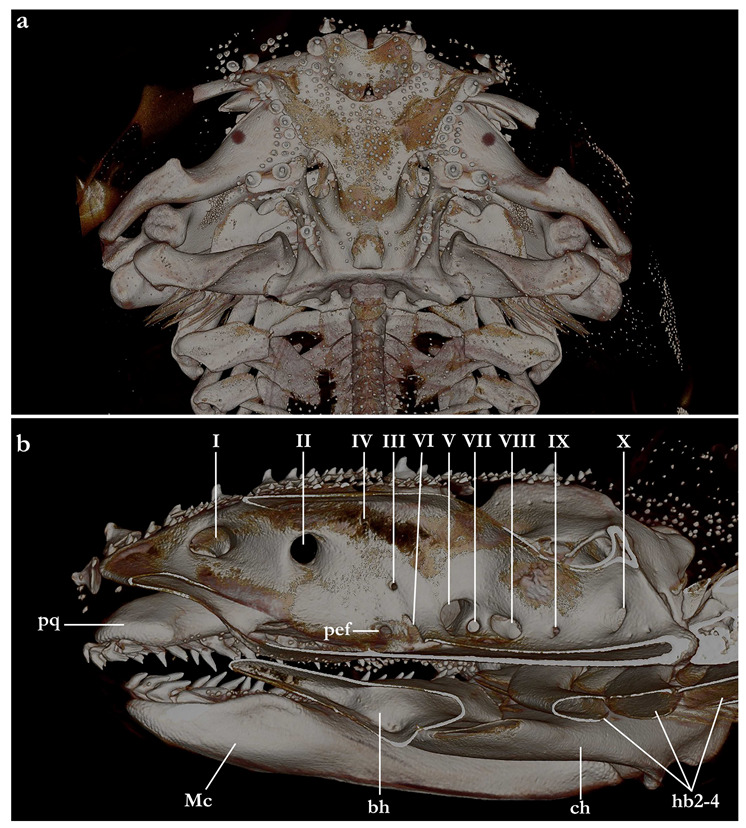
*Squatina africana*, ZMH 123064, juvenile female, 309 mm TL; micro-computed tomography scans of the skull: (**a**) dorsal view, (**b**) sagittal cross-section. Abbreviations: bh, basihyal; ch, ceratohyal; hb2-4, hypobranchials 2–4; Mc, Meckel’s cartilage; pef, pseudobranchial artery foramen; pq, palatoquadrate; I, olfactory bulb foramen; II, optic nerve foramen; III, oculomotor nerve foramen; IV, trochlear nerve foramen; V, trigeminal nerve foramen; VI, abducens nerve foramen; VII, facialis nerve foramen; VIII, auditory nerve foramen; IX, glossopharyngeal nerve foramen; X, vagus nerve foramen. Anterior to top in (**a**) and left in (**b**).

**Figure 21:**
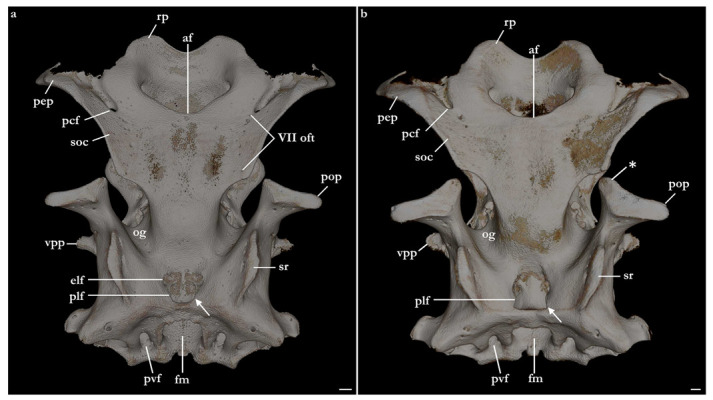
Micro-computed tomography scans of the neurocranium of (**a**) *Squatina leae* sp. nov., paratype, ZMH 26098, juvenile male, 249.6 mm TL and (**b**) *S. africana*, ZMH 123064, juvenile female, 309 mm TL in dorsal view. Abbreviations: af, anterior fontanelle; elf, endolymphatic foramen; fm, foramen magnum; og, orbital groove; pcf, preorbital canal foramen; pef, pseudobranchial artery foramen; plf, perilymphatic foramen; pop, postorbital process; pvf, posterior vein foramen; rp, rostral projections; soc, supraorbital crest; sr, sphenopterotic ridge; VII oft, foramina for rami of superficial ophthalmic nerve. The arrows indicate the margins of the perilymphatic foramen, distinctive to each species of *Squatina*. *Squatina leae* sp. nov. has concave margins, whereas in **S. africana*,* the margins are convex. The asterisk marks the anteromedial projection of the postorbital process, exclusive to *S. africana*. Anterior to top; scale bars: 2 mm.

**Figure 22:**
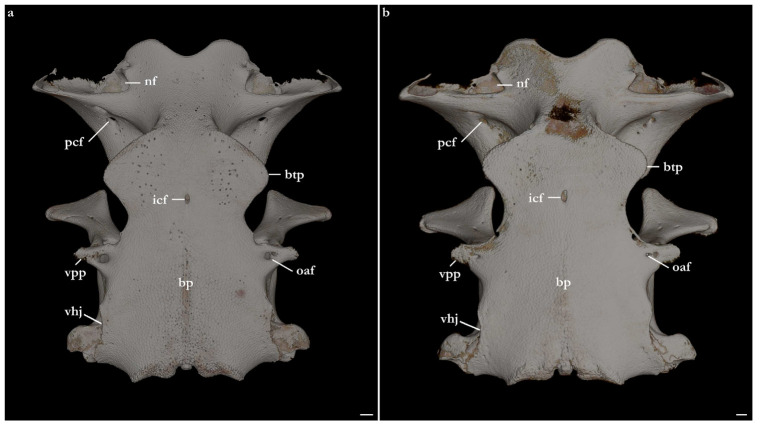
Micro-computed tomography scans of the neurocranium of (**a**) *Squatina leae* sp. nov., paratype, ZMH 26098, juvenile male, 249.6 mm TL and (**b**) *S. africana*, ZMH 123064, juvenile female, 309 mm TL in ventral view. Abbreviations: bp, basal plate; btp, basitrabecular process; icf, internal carotid foramen; nf, nasal foramen; oaf, orbital artery foramen; pcf, preorbital canal foramen; vhj, ventral hyoid junction; vpp, ventral postorbital process. Anterior to top; scale bars: 2 mm.

**Figure 23:**
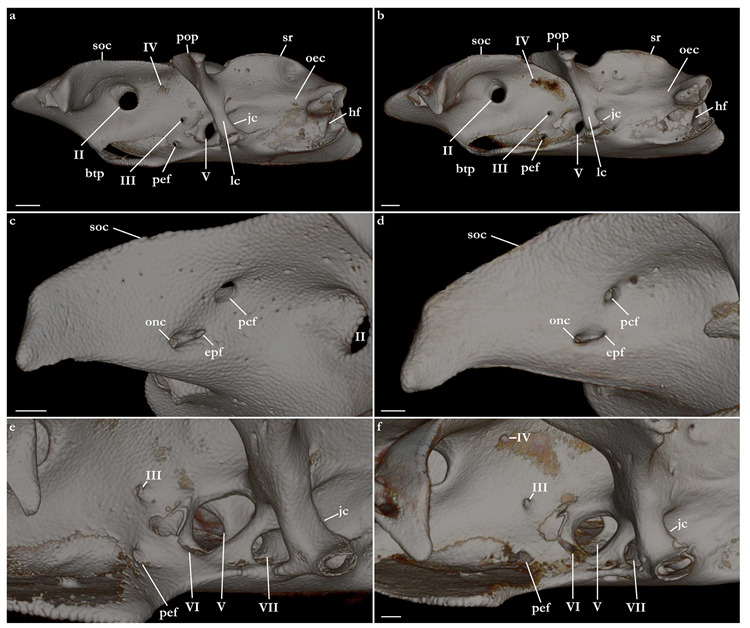
Micro-computed tomography scans of the neurocranium of (**a**,**c**,**e**) *Squatina leae* sp. nov., paratype, ZMH 26098, juvenile male, 249.6 mm TL and (**b**,**d**,**f**) *S. africana*, ZMH 123064, juvenile female, 309 mm TL in lateral view. Abbreviations: btp, basitrabecular process; epf, external profundus foramen; hf, hyomandibular facet; jc, jugular canal; lc, lateral commissure; oec, otic external canal; onc, orbitonasal canal; pcf, preorbital canal foramen; pef, pseudobranchial artery foramen; pop, postorbital process; soc, supraorbital crest; sr, sphenopterotic ridge; II, optic nerve foramen; III, oculomotor nerve foramen; IV, trochlear nerve foramen; V, trigeminal nerve foramen; VI, abducens nerve foramen; VII, facialis nerve foramen. Facing left; scale bars (**a**,**b**): 2 mm, (**c**,**d**,**f**): 1 mm.

**Figure 24:**
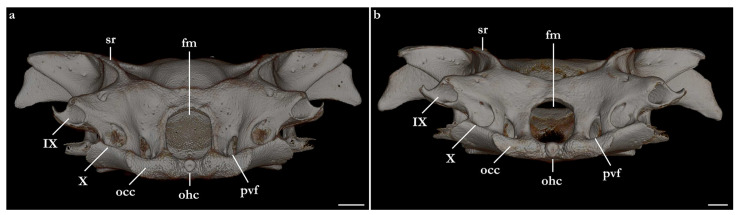
Micro-computed tomography scans of the neurocranium of (**a**) *Squatina leae* sp. nov., paratype, ZMH 26098, juvenile male, 249.6 mm TL and (**b**) *S. africana*, ZMH 123064, juvenile female, 309 mm TL in occipital view. Abbreviations: fm, foramen magnum; occ, occipital condyle; ohc, occipital hemicentrum; pvf, posterior vein foramen; sr, sphenopterotic ridge; IX, glossopharyngeal nerve foramen; X, vagus nerve foramen. Scale bars: 2 mm.

**Figure 25:**
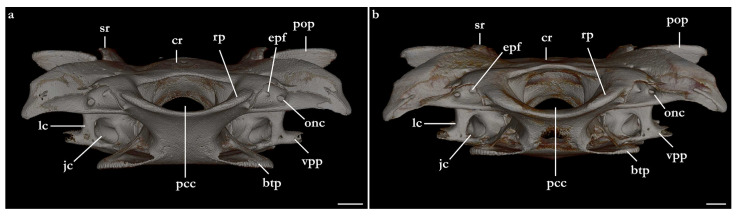
Micro-computed tomography scans of the neurocranium of (**a**) *Squatina leae* sp. nov., paratype, ZMH 26098, juvenile male, 249.6 mm TL and (**b**) *S. africana*, ZMH 123064, juvenile female, 309 mm TL in frontal view. Abbreviations: btp, basitrabecular process; cr, cranial roof; epf, external profundus foramen; jc, jugular canal; lc, lateral commissure; onc, orbitonasal canal; pcc, precerebral cavity; pop, postorbital process; rp, rostral projections; sr, sphenopterotic ridge; vpp, ventral postorbital process. Scale bars: 2 mm.

**Figure 26:**
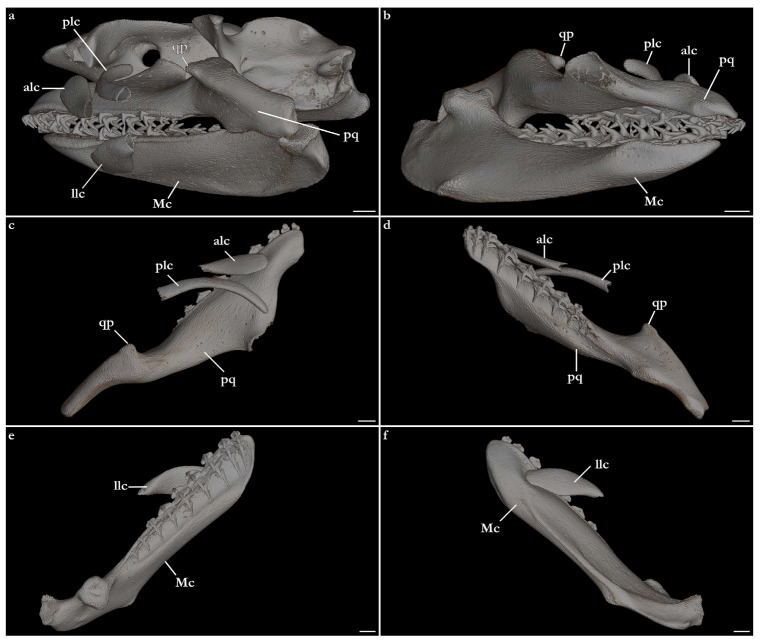
*Squatina leae* sp. nov., paratype, ZMH 26098, juvenile male, 249.6 mm TL; micro-computed tomography scans of (**a**) cranium and jaws in lateral view, (**b**) jaws in medial view, upper jaw in (**c**) dorsal and (**d**) ventral views, and lower jaw in (**e**) dorsal and (**f**) ventral views. Abbreviations: alc, anterior upper labial cartilage; llc, lower labial cartilage; Mc, Meckel’s cartilage; plc, posterior upper labial cartilage; pq, palatoquadrate; qp, quadrate process. Scale bars: 2 mm.

**Figure 27:**
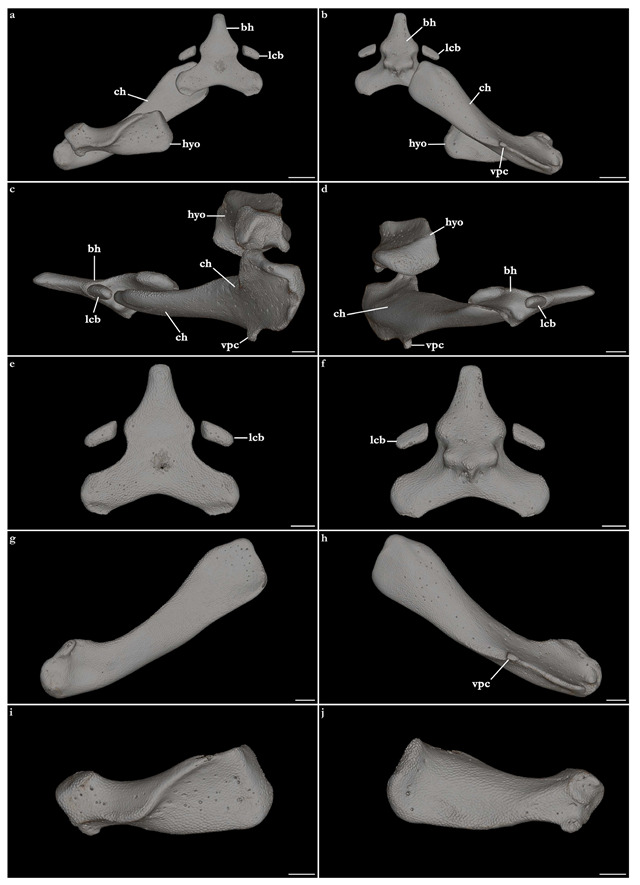
*Squatina leae* sp. nov., paratype, ZMH 26098, juvenile male, 249.6 mm TL; micro-computed tomography scans of hyoid arch in (**a**) dorsal, (**b**) ventral, (**c**) lateral, and (**d**) ventral views, basihyal in (**e**) dorsal and (**f**) ventral views, ceratohyal in (**g**) dorsal and (**h**) ventral views, and hyomandibula in (**i**) dorsal and (**j**) ventral views. Abbreviations: bh, basihyal; ch, ceratohyal; hyo, hyomandibula; lcb, lateral cartilage of basihyal; vpc, ventral process of ceratohyal. Scale bars (**a**,**b**): 4 mm, (**c**–**j**): 2 mm.

**Figure 28:**
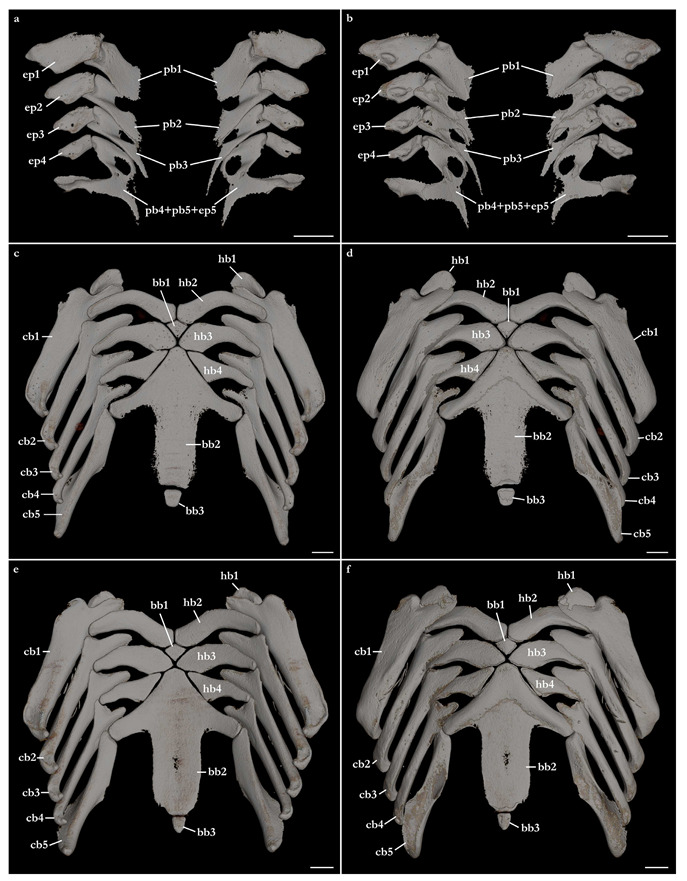
Micro-computed tomography scans of the branchial arches of (**a**–**d**) *Squatina leae* sp. nov., paratype, ZMH 26098, juvenile male, 249.6 mm TL and (**e**,**f**) *S. africana*, ZMH 123064, juvenile female, 309 mm TL. (**a**,**b**) Epi- and pharyngobranchials in (**a**) dorsal and (**b**) ventral views, (**c**–**f**) basi-, cerato-, and hypobranchials in (**c**,**e**) dorsal and (**d**,**f**) ventral views. Abbreviations: bb1–bb3, basibranchials 1–3; cb1–5, ceratobranchials 1–5; ep1–5, epibranchials 1–5; hb1–4, hypobranchials 1–4; pb1–5, pharyngobranchials 1–5. Anterior to top; scale bars: 5 mm.

**Figure 29:**
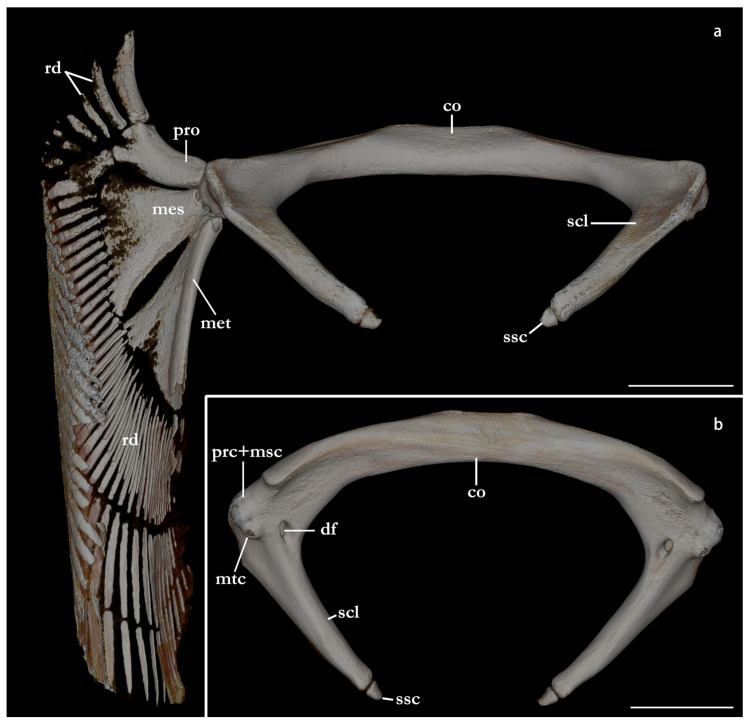
*Squatina leae* sp. nov., paratype, ZMH 26098, juvenile male, 249.6 mm TL; micro-computed tomography scans of (**a**) pectoral fin and girdle in dorsal view and (**b**) pectoral girdle in ventral view. Abbreviations: co, coracoid bar; df, diazonal foramen; mes, mesopterygium; met, metapterygium; mtc, metacondyle; prc + msc, condyle for propterygium and mesopterygium; pro, propterygium; rd, radials; scl, scapula; ssc, suprascapula. Scale bars: 10 mm.

**Figure 30:**
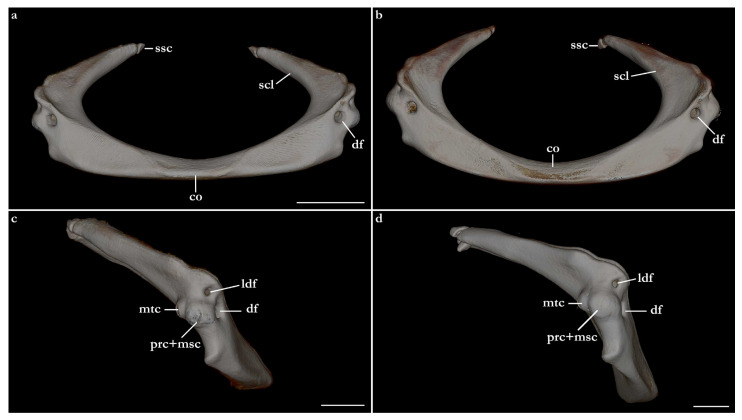
Micro-computed tomography scans of the pectoral girdle of (**a**,**c**) *Squatina leae* sp. nov., paratype, ZMH 26098, juvenile male, 249.6 mm TL and (**b**,**d**) *S. africana*, ZMH 123064, juvenile female, 309 mm TL in (**a**,**b**) frontal and (**c**,**d**) lateral views. Abbreviations: co, coracoid bar; df, diazonal foramen; ldf, lateral diazonal foramen; mtc, metacondyle; prc + msc, condyle for propterygium and mesopterygium; scl, scapula; ssc, suprascapula. Scale bars (**a**): 10 mm, (**c**,**d**): 5 mm.

**Figure 31:**
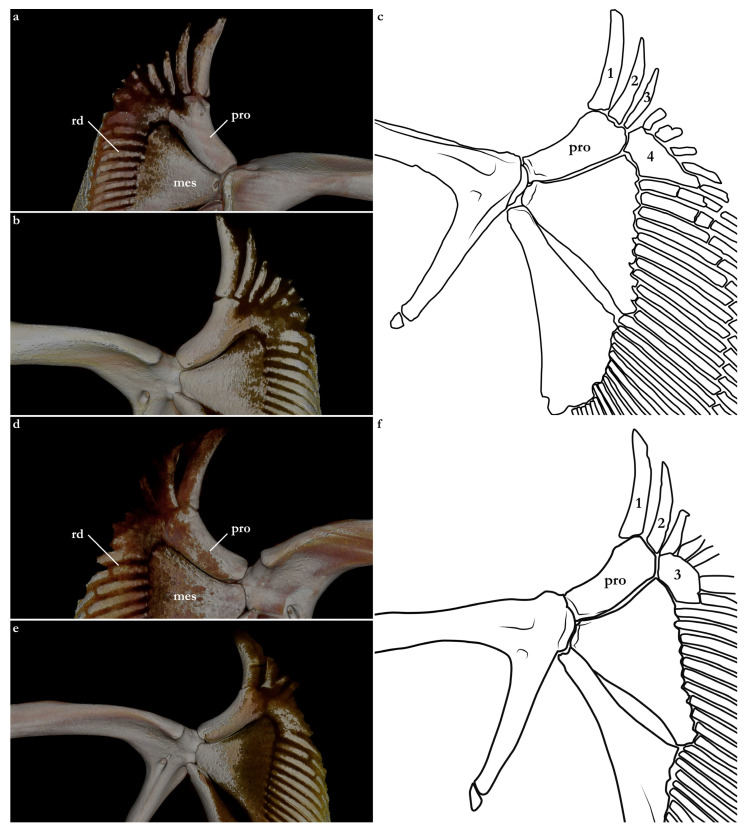
Micro-computed tomography scans (**a**,**b**,**d**,**e**) and drawings (**c**,**f**) of the anterior pectoral skeleton of (**a**,**b**) *Squatina leae* sp. nov., paratype, ZMH 26098, juvenile male, 249.6 mm TL, (**c**) *S. leae* sp. nov., holotype, CMFRI GA. 15.2.5.4, adult male, 671 mm TL, (**d**,**e**) *S. africana*, ZMH 123064, juvenile female, 309 mm TL, (**f**) *S. africana*, holotype, BMNH 1906.11.19.21, late subadult male, 837 mm TL. The numbers in (**c**,**f**) indicate the number of radials articulating with the propterygium. Abbreviations: mes, mesopterygium; pro, propterygium; rd, radials.

**Figure 32:**
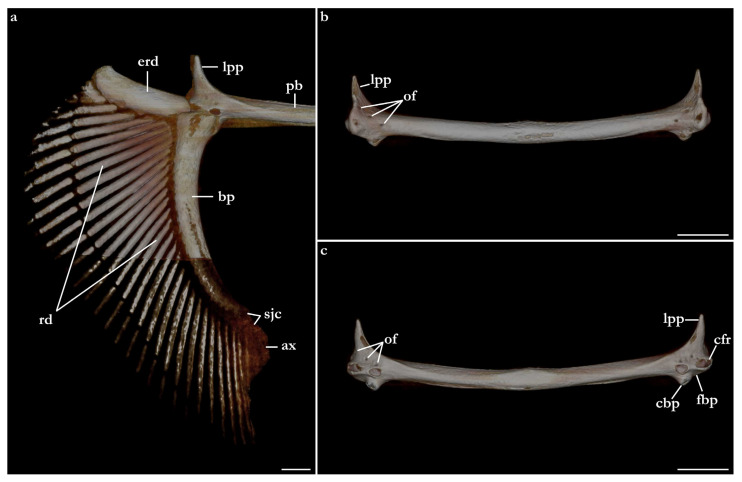
*Squatina leae* sp. nov., paratype, ZMH 26098, juvenile male, 249.6 mm TL; micro-computed tomography scans of (**a**) pelvic fin in ventral view, as well as pelvic girdle in (**b**) dorsal and (**c**) ventral views. Abbreviations: ax, axial cartilage; bp, basipterygium; cbp, condyle for basipterygium; cfr, condyle for first enlarged pelvic radial; erd, enlarged radial; fbp, facet for basipterygium; lpp, lateral prepelvic process; of, obturator foramina; pb, pubioischiadic bar; rd, radials; sjc, stem-joint cartilages. Scale bars: 5 mm.

The skeleton of both species is very similar to those described from other species of angel sharks [24,35,36,62,63,64,65,66,67,68,69]. Most of the variation observed occurs in the shape and extension of margins of cavities and processes. For *Squatina africana*, the first descriptions of the chondrocranium were published by Mollen et al. [70]. Descriptions presented in the present paper are based on the communalities shared by the specimens from both *S. leae* sp. nov. and *S. africana* and include notes on observed differences between these taxa. The skeleton descriptions are based on micro-computed tomography (mCT) scans (Figure 19, Figure 20, Figure 21, Figure 22, Figure 23, Figure 24, Figure 25, Figure 26, Figure 27, Figure 28, Figure 29, Figure 30, Figure 31 and Figure 32) of juvenile male paratype ZMH 26098 of *S. leae* sp. nov. and juvenile female non-type ZMH 123064 of *S. africana* except for descriptions of the clasper skeleton, which are based on radiographs of the adult male holotype of *S. leae* sp. nov. (CMFRI GA. 15.2.5.4) and adult male non-type SAIAB 187381 of *S. africana*. Overview mCT scans of the head regions of juvenile male paratype ZMH 26098 of *S. leae* sp. nov. and juvenile female non-type ZMH 123064 of *S. africana* can be found in Figure 19 and Figure 20.

***Neurocranium (Figure 21, Figure 22, Figure 23, Figure 24 and Figure 25)***—The neurocrania of *Squatina leae* sp. nov. and *S. africana* are slightly longer than their greatest width, which is situated at the posterior region of the nasal capsules and the postorbital processes.

*Rostral region*. Rostral region short, less than one-fifth of neurocranium length, with a pair of broad, semi-circular rostral projections, antimeres connected by a concave margin. Dorsal surface of the rostral region centrally concave, projecting dorsolaterally to form the margins of the anterior fontanelle. Ventral margin of the rostral region with a convex surface and projecting posteroventrally, being confluent with the anterior margins of the basitrabecular processes. The margin of the anterior fontanelle of *Squatina leae* sp. nov. forms a V-shaped outline (similar to that described for *S. argentina*; [24,66]), whereas in *S. africana*, the anterior fontanelle is broad, has a more rounded outline, and its margins are irregular (vs. continuous in *S. leae* sp. nov.).

*Nasal capsules*. Most of the anterior region of the nasal capsules of *Squatina* is not calcified (see [24]). The calcified region of the nasal capsules is restricted to their posterior region, delimiting the nasal foramina ventrally and the posterior wall of the nasal capsules.

*Orbital region*. The orbital region of the neurocranium of *Squatina* is delimited anterodorsally by the dorsal opening of the external profundus foramen and the anterior tip of the supraorbital crest, anteroventrally by the basitrabecular process [6,24], posterodorsally by the postorbital process, and posteroventrally by the lateral commissure. The dorsal surface of the orbital region comprises the supraorbital crest, which is arranged obliquely in dorsolateral orientation, having its lateral margin projecting posteromedially, confluent with the medial walls that form the orbital groove. The lateral margin of the supraorbital crest has a triangular process, the supraorbital flange [24,66]. The supraorbital flange of *S. leae* sp. nov. is further posteriorly positioned compared to *S. africana*: the distance from the anterior edge of the supraorbital crest to the flange is 40.2% of the neurocranial length in *S. leae* sp. nov. vs. 37.7% in *S. africana*. The anteromedial portion of the supraorbital crest has a deep groove where, in its posterior edge, the dorsal opening of the preorbital canal sits. In the anteromedial edge of the groove, a small, elliptical opening forms the external profundus foramen. Posterior to the dorsal foramen of the preorbital canal, seven small foramina for the passage of the accessory rami of the superficial ophthalmic trunk are aligned longitudinally, with the posteriormost foramen positioned adjacent to the supraorbital flange.

Preorbital wall posterodorsally oriented, with a smooth, straight surface. The lateral tip of the preorbital wall forms a triangular process projected ventrolaterally. The median region of the preorbital wall has four foramina: positioned centrally is the ventral opening of the preorbital canal, the largest foramen of the preorbital wall. Posterodorsally positioned is the anteriormost foramen of the accessory rami of the superficial ophthalmic trunk. Ventrolaterally from the preorbital canal, an elliptical groove bears a pair of foramina: the medial opening is the ventral aperture of the external profundus foramen. The lateral foramen is the posterior opening of the orbitonasal canal, which opens anteriorly inside the nasal capsule. The median wall of the orbital region is concave, ventromedially oriented, delimited ventrally by the basitrabecular process. The large, circular aperture positioned anterodorsally is the foramen for the optic nerve (II). Posteriorly from the optic nerve, dorsal and centrally in the median orbital wall, sits a small circular foramen for the passage of the trochlear nerve (IV). From the trochlear foramen, a circular foramen posteroventrally opens for the passage of the oculomotor nerve (III) and the foramen for the pseudobranchial artery sits ventrally, located adjacent to the posteroventral boundary of the basitrabecular process. The foramen for the trigeminal nerve (V) is oval, as large as the foramen for the optic nerve, positioned inside a groove in the posteroventral region of the median orbital wall. Inside the groove, the abducens foramen (VI) is positioned anterolaterally from the trigeminal nerve foramen (V), whereas the foramen for the hyomandibular branch of the facial nerve (VII) is located posterior from the trigeminal nerve foramen, opening into the jugular canal.

The dorsal postorbital process is robust, formed by a subcylindrical dorsolateral projection extending from the orbital groove for the orbital process of the palatoquadrate to the level of the supraorbital crest. The dorsal tip of the dorsal postorbital process is flat, expanded, and triangular-shaped, supporting a pair of conical enlarged dermal denticles. In *Squatina leae* sp. nov., the anteromedial edge of the dorsal surface of the dorsal postorbital process is slightly projected anteriorly, having a shallow, concave anterior margin. Conversely, in *S. africana*, the anteromedial edge of the dorsal surface of the dorsal postorbital process has a rounded, anteriorly directed projection, resulting in a deeply concave anterior margin. The distance between the anterodorsal margin of the dorsal postorbital process and the supraorbital flange is 3.4% of the neurocranial length in *S. leae* sp. nov. and 3% of the neurocranial length in *S. africana*. The anterior surface of the dorsal postorbital process is concave, with a pair of foramina positioned centrally for passage of rami of the orbital artery. The ventral region of the dorsal postorbital process is delimited by the anterior opening of the jugular canal and the anterior surface of the lateral commissure. The posterior surface of the dorsal postorbital process is convex, with its medial portion forming the lateral wall of the orbital groove and lateral portion being delimited by the otic capsule, posterior opening of the jugular canal, and the lateral surface of the lateral commissure. Three to five foramina are present in the posterior surface of the dorsal postorbital process for passage of branches of the orbital artery [71].

*Cranial roof*. Tectum orbitale slightly wider but deeper than the otic tectum. The surface of tectum orbitale is slightly convex, with a shallow groove for the pineal fontanelle. In the specimen of *Squatina africana*, the foramen of the pineal fontanelle is still present. In the specimen of *S. leae* sp. nov., the foramen is not present, although the homologous groove for its opening is present.

*Otic capsule*. The otic capsule is large, more than one-third of the length of the neurocranium, with a deep concave dorsal surface. The sphenopterotic ridge forms the dorsolateral edges of the otic capsule, extending from the base of the postorbital process to the boundary with the occipital region. The sphenopterotic ridge has a flat, triangular dorsal surface that supports one to three conical, enlarged dermal denticles. The base of the medial surface of the sphenopterotic ridge has two to four foramina allowing the passage of branches of the orbital artery to connect with the dorsal superficial branchial artery [71]. The lateral surface of the sphenopterotic ridge is mostly convex, with a semi-cylindrical and vertically arranged bulge in the middle of the ridge. Two to three foramina for the passage of branches of the orbital artery are present in the anterior region of the lateral surface of the sphenopterotic ridge. The parietal fossa is positioned in the posteromedial region of the otic capsule, entirely elliptical in *Squatina leae* sp. nov., whereas in *S. africana*, the parietal fossa is elliptical only anteriorly, but has a straight posterior margin. Endolymphatic foramen positioned anteriorly in the parietal fossa, oval-shaped, small, approximately half the diameter of the perilymphatic foramen, and separated from the perilymphatic foramen by a small commissure. Perilymphatic foramen circular, extending through the posterolateral surface of the parietal fossa. In *S. leae* sp. nov., the dorsal margin of the perilymphatic foramen is concave vs. convex in *S. africana*.

Lateral surface of the otic capsule mostly convex, with a longitudinal ridge corresponding to the lateral portion of the external semicircular canal. The longitudinal ridge is continuous with the posterior tubular process for the foramen of the glossopharyngeal nerve (IX). The lateral margin of the glossopharyngeal process has a dorsally projected, concave ridge. The posteroventral region of the otic capsule is concave, forming the hyomandibular facet. The anterior margin of the otic capsule is formed by the lateral commissure, which extends from the base of the postorbital process to the lower postorbital process. The ventral region of the lateral commissure is perforated by a large, elliptical, jugular canal, which opens anteriorly in the ventrolateral portion of the orbital region, and has a posterior opening on the anteroventral region of the otic capsule.

*Basal plate*. Basal plate flat and large, its length two-thirds of the neurocranial length, forming the neurocranial floor. Anterior region of the basal plate (anterior to the ventral postorbital process) hexagonal-shaped, posterior region rectangular. The ventral margins of the basitrabecular process are parabola-shaped. In *Squatina leae* sp. nov., the ventral margins of the basitrabecular process are wider (27.4% of neurocranial length), with a convex anterior margin and concave posterior margin; in *S. africana*, the length of the base of the basitrabecular process is 23.5% of the neurocranial length and both anterior and posterior margins are concave. Width of the neurocranium from the edges of the basitrabecular process 48.5% of neurocranial length in *S. leae* sp. nov. and 47.4% of neurocranial length in *S. africana*.

Elliptical, single internal carotid foramen positioned on the ventral longitudinal axis of the neurocranium, horizontally aligned with the posterior portion of the ventral margin of the basitrabecular process. The lateral margin of the basal plate is deeply concave posterior to the ventral margin of the basitrabecular process and continuous to the anterior margin of the ventral postorbital process. The ventral postorbital processes are triangular, projected laterally, with a rounded lateral edge. A circular foramen for a branch of the orbital artery is present in the middle of the base of the ventral postorbital process. The anterior edge of the ventral postorbital process has a semi-circular indentation on both left and right processes in *Squatina leae* sp. nov.; in *S. africana*, the indentation on the left side is entirely enclosed in a foramen; on the right side, however, the enclosing of the foramen is only partial. The indentation or foramina are likely to allow the passage of branches of the orbital artery [71]. The variation between indentation or foramina for branches of the orbital artery is interpreted as an intraspecific variation, similar to that described by Vaz and Carvalho [24].

The posterior region of the basal plate has notochord remnants still visible across its longitudinal axis. The surface of the posterolateral region of the basal plate is slightly concave, having both posterolateral and posterior margins projecting ventrally. The lateral margins of the posterior region of the basal plate are sinusoidal-shaped, forming two short processes that delimit ventrally the hyomandibular facet. The anterior process of the hyomandibular facet is triangular in *Squatina leae* sp. nov. vs. rounded in *S. africana*. In both species, the posterior process of the hyomandibular facet is rounded.

*Occipital region*. The occipital region is slightly slanted posteroventrally, twice wider than deep, with its surface slightly concave medially. The dorsal margin of the occipital region has a foramen on each side at the level of the base of the tubular projection for the opening of the glossopharyngeal nerve (IX). Only on the left side of the neurocranium of *Squatina africana* three small foramina were found in that region. Foramen magnum circular to hexagonal, slightly deeper than wide. A pair of foramina is present on each side of the foramen magnum. Medially, a vertically elliptical foramen allows the passage of the posterior cerebral vein. The circular, laterally positioned, and posterolaterally oriented foramen is for the passage of the vagus nerve (X). A small, circular foramen is located on the dorsal region of the foramen for the vagus nerve. Occipital condyle comma-shaped, large, its width one-quarter of the width of the occipital surface. Dorsal margin of occipital condyle concave, ventral margin sinusoidal-shaped. A circular occipital hemicentrum is positioned between antimeres of occipital condyles, ventral to the foramen magnum.

***Jaws and labial cartilages (Figure 26)***. Upper and lower jaws robust and long, 1.2 times the length of the neurocranium, arranged posterolaterally at a 45° angle from the longitudinal midline. Palatoquadrate wider and thinner anteriorly, narrower and stockier posteriorly; dorsal surface convex; ventral surface concave anteriorly, with a deep groove for attachment of tooth rows, convex posteriorly. The anterior portion of the medial margin of the palatoquadrate has a broad posteromedial projection, forming the calcified base of the orbital process of the palatoquadrate (not visible on CT scans; for further details, see [24]). The posterolateral region of the palatoquadrate has a robust, pyramidal anterodorsal projection, the quadrate process of the palatoquadrate. The anterior edge of the palatoquadrate extends anteriorly from the anterior edge of Meckel’s cartilage. The posterior edge of the palatoquadrate is sub-cylindrical and articulates with the articular condyle of Meckel’s cartilage.

Meckel’s cartilage anterodorsal surface concave, with a demarked groove for attachment of tooth rows. Posterodorsal surface deeply convex, with a cubical dorsal projection for support of the spiracular cartilage [24] and articulating with the distal part of the hyomandibula, forming the double jaw-joint. Ventral surface convex, with an angular ventral margin that extends posteriorly to the distal end of Meckel’s cartilage. A small, semi-elliptical process is present on the posteroventral region of the ventral margin of Meckel’s cartilage. The posterior edge projects dorsally and has a flat, triangular surface, forming the articular condyle of Meckel’s cartilage that articulates with the palatoquadrate.

Two pairs of upper labial cartilages and one pair of lower labial cartilages are present. The lateralmost portion of the labial cartilages is not visible, for lacking tessellated calcification. Anterior upper labial cartilage flattened, medially wide (twice wider than posterior upper labial cartilage) and tapering laterally, with its medial edge sitting on a groove of the dorsal surface of the palatoquadrate. Posterior upper labial cartilage elongated and flattened, with its extremities curved posteriorly, having its calcified portion being almost twice longer than the anterior upper labial cartilage. The medial edge of the posterior upper labial cartilage is positioned on the base of the orbital process of the palatoquadrate. Lower labial cartilage flattened, elliptical, medially wider than the lateral portion. The medial edge of the lower labial cartilage sits on a groove of the ventral surface of Meckel’s cartilage.

***Hyoid arch (Figure 27)***. Hyomandibula robust, club-shaped, medially flatter relative to the lateral portion, with a rounded and ventrally projected medial margin that articulates with the neurocranium in the hyomandibular facet. Lateral portion of the hyomandibula cubical, with a posteroventral process projecting from the posteroventral edge of the lateral surface of the hyomandibula. Lateral surface of the hyomandibula concave. Dorsal surface of the hyomandibula mostly concave, with a sinusoid ridge extending from the anteromedial margin to the posterolateral margin of the dorsal surface for attachment of the *constrictor hyoideus dorsalis* muscle. Ventral surface concave, with a small convex projection on the lateral portion continuous to the ventrolateral edge that articulates with the ceratohyal.

Ceratohyal long, two times longer than the hyomandibula, anteriorly flat and wider than the posterior region. Anteromedial edge rectangular, posterolateral edge rounded. Anterodorsal and anteroventral surfaces mostly flat. Posterodorsal surface of the ceratohyal convex and gradually increasing in depth, with the posterior margin projecting dorsally forming a ridge. Posteroventral surface concave anteriorly, with an elongate ridge projecting from its posterior margin. The ventral ridge of the ceratohyal has a rounded projection on its anterior edge.

Basihyal triangular, with a rhomboidal anterior portion and two rectangular projections with rounded edges extending posterolaterally. The dorsal surface is smooth and flat. The ventral surface is mostly smooth, with a large median ridge oriented transversely in the posterior region of the basihyal for articulation with the anterior edges of the ceratohyal. A pair of rectangular lateral cartilages of the basihyal is present, with each antimere positioned lateral to the anterolateral margin of the basihyal (by the origin of the median ridge).

***Branchial arches (Figure 28)***. Pharyngobranchials trapezoidal and posteromedially directed. Pharyngobranchial one not fenestrated and with an angular posterior edge. Pharyngobranchials two to four with a rounded fenestra on the anteromedial region and with an elongate posterior edge. Pharyngobranchials four and five fused to epibranchial four, forming a Y-shaped element. Epibranchials are trapezoidal, with epibranchial one being 1.5 times longer than epibranchial two. Epibranchials one to four posterolaterally oriented; epibranchial five laterally oriented and fused to pharyngobranchials four and five. Ceratobranchials are the longest elements of the branchial arches and are oriented posterolaterally. Ceratobranchial one is two times wider than ceratobranchial two and has a deeply concave anterior margin that articulates with hypobranchial one. Ceratobranchials two to four have a shallow to straight anterior margin, with a triangular medial edge for articulating with hypobranchials two to four. Ceratobranchial five with a rounded anterior margin, lacking a triangular medial extension as observed in the anterior ceratobranchials, and articulating with the lateral edges of the cardiobranchial (basibranchial two) cartilage. Posterior portion of ceratobranchial five with a semi-circular ventrolateral projection. Hypobranchials are comma-shaped, with a convex anterior margin and concave posterior margin. Hypobranchial one is small, its length approximately one-third of the length of ceratobranchial two, located on the anterolateral region of the branchial arches, closely articulating with the anteromedial portion of ceratobranchial one. Hypobranchial two with angular median margins, medial edge slightly wider than the rounded, posterior edge. Hypobranchial three with a wide, angular medial margin, tapering laterally. The lateral edge of hypobranchial three of *Squatina leae* sp. nov. is narrower than in *S. africana*; the depth of the posterior edge is 0.34 times the depth of the medial edge vs. 0.4 times in *S. africana*. Hypobranchial four with straight medial margins and acute anteromedial edges. The depth of the medial region of hypobranchial four is four times the depth of the lateral region. Basibranchial series comprises three cartilages. Basibranchial one is a small, rhomboidal cartilage positioned between the medial edges of hypobranchials two and three. Basibranchial two (cardiobranchial) is arrow-shaped and large, its length as long as ceratobranchial one, and also wide, with its width similar to the width of both hypobranchial cartilages four. The anterior portion of the basibranchial two cartilage is triangular with two posterolateral projections formed by the fusion of the pair of hypobranchial five to basibranchial two [6,72]. Basibranchial three is a small, rectangular median cartilage positioned posterior to the posterior margin of the cardiobranchial cartilage. Basibranchial three of *S. leae* sp. nov. is larger than in *S. africana*. In *S. leae* sp. nov., the width of basibranchial three is 0.4 times the width of basibranchial two vs. 0.28 in *S. africana*.

***Pectoral girdle and fins (Figure 29, Figure 30 and Figure 31)***. Scapulocoracoid cartilage U-shaped, robust, as large as the lower jaws. The coracoid bar is slightly arched posteriorly, dorsoventrally flattened in its median region, and subcylindrical in its lateral region. The ventroanterior margin of the coracoid bar has a short, but robust, triangular posterior process positioned in its lateral region for attachment of muscle bundles of *depressor pectoralis II* muscle (for further details, see da Silva and Datovo [73]). The scapula is posterodorsally oriented, pyramidal ventrally, and gradually transitioning to a cylindrical shape posteriorly. The base of the scapula has three openings, an anterior, a lateral, and a posterior diazonal foramen. The anterior diazonal foramen is located in the anterior surface of the base of the scapula. The lateral diazonal foramen is positioned on the base of the lateral surface of the scapula, dorsal to the anterior articular condyle. The posterior diazonal foramen opens in the ventromedial surface of the base of the scapula. Two rounded, articular condyles are present in the lateral surface of the base of the scapula. The anterior condyle articulates with the propterygium and the mesopterygium. A smaller, posterior condyle articulates with the metapterygium. A small, pyramidal suprascapula cartilage is positioned dorsal to the dorsoposterior tip of the scapula. The orientation between the scapula and the coracoid bar varies among *Squatina leae* sp. nov. and *S. africana*. The scapula of *S. africana* is oriented in an angle of almost 90° in relation to the anterior surface of the coracoid bar. The anterior surface of the coracoid bar is projected almost dorsally, with the scapula projecting mostly posteriorly. The angle between the scapula and the anterior surface of the coracoid bar in *S. leae* sp. nov. is lower, without a marked orientation change between the anterior surface of the coracoid bar and the scapula.

Three basal radials are present supporting pectoral radials. Propterygium oriented anterolaterally, rectangular-shaped, with a concave lateral margin. In *Squatina africana*, as in the other examined species of angel sharks, the propterygium supports three pectoral radials (see also [14,24]). The propterygium of *S. leae* sp. nov., conversely, supports four pectoral radials (Figure 31). This unique arrangement of the propterygium and radials might be a potential autapomorphy of *S. leae* sp. nov. and is consistently found in all seven type specimens examined via CT scans and/or radiographs. Mesopterygium trapezoidal, as long as the propterygium, but its lateral margin is two times wider than the largest breadth of the propterygium. A total of 12–13 pectoral radials articulate with the mesopterygium. Metapterygium pad-shaped, narrow anteriorly and expanding posteriorly, 1.5 times longer than the propterygium. The posteromedial edge of the metapterygium has two small, rectangular distal segments. The metapterygium of *S. leae* sp. nov. supports 21–24 radials; in *S. africana*, 22–26 radials articulate with the metapterygium.

***Pelvic girdle and fins (Figure 32)***. Pubioischiadic bar elongate, with anterior margin slightly concave and posterior margin convex. Lateral edges expanded dorsoventrally, with the anterolateral edge bearing a triangular lateral prepelvic process projecting anteriorly (see [36]). The posterolateral region of the pubioischiadic bar is wider than the anterior edge and bears two condyles and a facet in its ventrolateral surface. The elliptical condyle for articulation with the first enlarged pelvic radial is located at the lateral surface of the pubioischiadic bar. The condyle and facet located at the posteroventral edge of the pubioischiadic bar articulates with the basipterygium. Three obturator foramina are present on the lateral region of each side of the pubioischiadic bar on both dorsal and ventral surfaces. Basipterygium elongate, rod-shaped, widest at its anterior region, with concave medial margin and convex lateral margin. The posterior edge of the basipterygium has two rectangular stem-joint cartilages. A total of 24–28 radials articulate with the basipterygium. The first, enlarged radial is rectangular, as wide as the basipterygium, and its length is one-third of the length of the basipterygium. Five secondary radials articulate with the first enlarged radial.

***Clasper (Figure 4 and Figure 14).*** Axial cartilage elongate and dorsoventrally flattened, with a shallow and elongate triangular process on the anterior portion of the medial margin of the axial cartilage. Dorsal marginal cartilage triangular, small, less than one-sixth of axial cartilage length. Accessory dorsal marginal cartilage elongated, with acute anterior and posterior edges, positioned laterally to the dorsal marginal cartilage. Ventral marginal cartilage trapezoidal in ventral view, projecting dorsally and surrounding the base of the globular rod, with the anterior margin of the ventral marginal cartilage extending posterolaterally. Ventral marginal cartilage dorsally thick and with a triangular outline. A long globular cartilaginous rod extends through the anteromedial portion of the clasper, forming the external wall of the clasper groove.

Clasper glans supported by six cartilages. End-style slender, extending posteriorly from distal edge of axial cartilage to posterior tip of clasper. Dorsal terminal 1 cartilage rectangular, extending through the dorsomedial surface of the glans, positioned dorsal to end-style and supporting the cover rhipidion. Dorsal terminal 2 cartilage soft and uncalcified, forming the rhipidion. Dorsal terminal 3 cartilage small, trapezoidal in cross-section, and entirely encapsulated by the ventral marginal cartilage. Ventral terminal 1 cartilage trapezoidal, with a concave anterior portion of the lateral margin that delimits the pseudopera. Ventral terminal 1 extends throughout the glans, with its dorsal surface forming the clasper glans floor. Ventral terminal 2 cartilage rectangular, with concave anterior margin and convex posterior margin. The anterolateral edge of ventral terminal 2 bears a conical process that extends to middle of the lateral margin of the ventral marginal cartilage. Dorsal surface of angular process of the ventral terminal 2 cartilage delimits the anterior and ventral walls of the pseudopera.

### 3.5. Molecular Data

**Figure 33:**
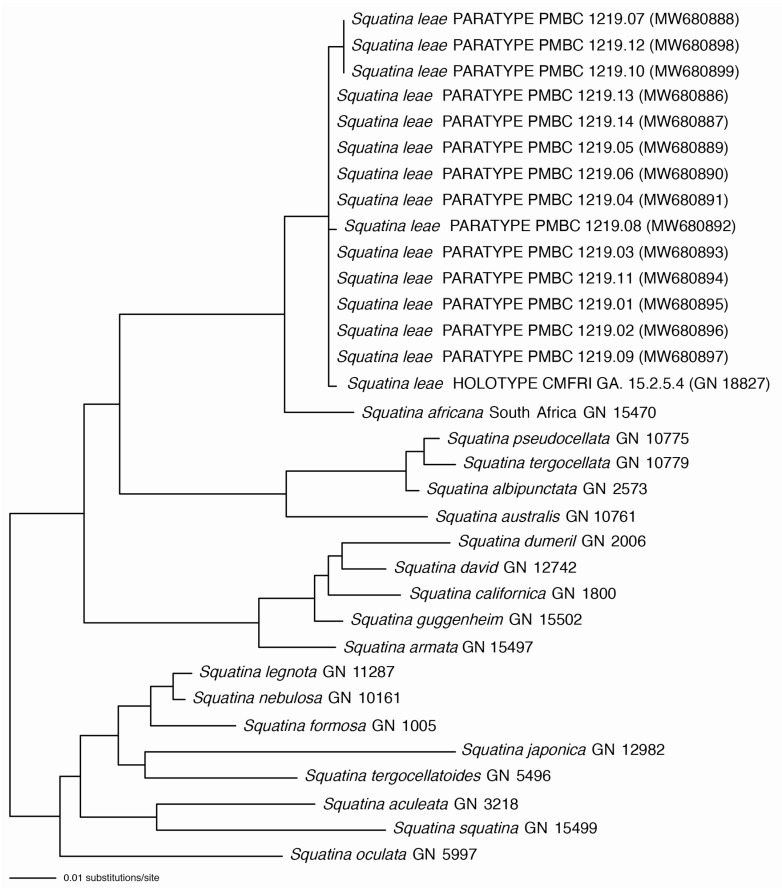
Maximum Likelihood Tree inferred from aligned CO1 sequences based on a General Time-Reversible Model with accommodation for both rate variation among sites and invariant sites. Parameter values estimated from the data. Type specimens noted where appropriate.

**Figure 34:**
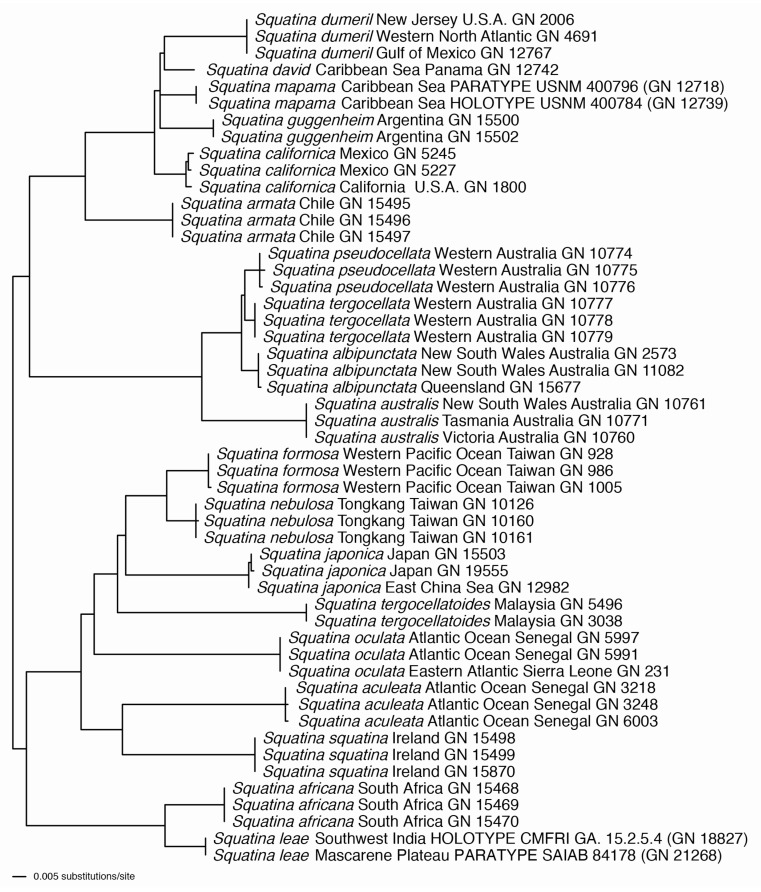
Maximum Likelihood Tree of phylogenetic relationships inferred from aligned NADH2 sequences based on a General Time-Reversible Model, with accommodation for both rate variation among sites and invariant sites. Parameter values were estimated from the data. Inferred relationships show strong geographical association. Type specimens noted where appropriate.

The Maximum Likelihood phylogenetic estimates for both the CO1 and NADH2 data sets are shown in Figure 33 and Figure 34, respectively. In both trees, *Squatina leae* sp. nov is clearly distinct from *S. africana*, the species to which it is most closely related, supporting the recognition of *S. leae* sp. nov as a distinct species. It should be noted that the phylogenetic estimates among the *Squatina* species examined are similar in both data sets but are not identical. Both tree topologies are broadly consistent in supporting biogeographically coherent groupings as has been shown previously [74]. We caution that, while the broad topological congruence observed is likely the consequence of a shared phylogenetic history, the similarity between the two trees could also be because both gene sets are derived from the mitochondrial genome and are, therefore, not independent.

## 4. Discussion

*Squatina* contains 24 valid species, including the new species described herein: *Squatina aculeata* Cuvier, 1829; *Squatina africana* Regan, 1908; *Squatina albipunctata* Last & White, 2008; *Squatina argentina* (Marini, 1930); *Squatina armata* (Philippi, 1887); *Squatina australis* Regan, 1906; *Squatina caillieti* Walsh, Ebert & Compagno, 2011; *Squatina californica* Ayres, 1859; *Squatina david* Acero P., Tavera, Anguila & Hernández, 2016; *Squatina dumeril* Lesueur, 1818; *Squatina formosa* Shen & Ting, 1972; *Squatina guggenheim* Marini, 1936; *Squatina japonica* Bleeker, 1858; *Squatina leae* sp. nov.; *Squatina legnota* Last & White, 2008; *Squatina mapama* Long, Ebert, Tavera, Pizarro & Robertson, 2021; *Squatina nebulosa* Regan, 1906; *Squatina occulta* Vooren & da Silva, 1991; *Squatina oculata* Bonaparte, 1840; *Squatina pseudocellata* Last & White, 2008; *Squatina squatina* (Linnaeus, 1758); *Squatina tergocellata* McCulloch, 1914; *Squatina tergocellatoides* Chen, 1963; *Squatina varii* Vaz and Carvalho, 2018.

***Interspecific comparisons***—The two western Indian Ocean species of *Squatina* are apparently allopatric with the distribution of *S. africana* being restricted to the eastern African coast and Madagascar, while the new species occurs on the oceanic Mascarene Plateau, as well as off southwestern India. They can further be differentiated based on molecular data, as well as morphological, morphometric, and meristic characteristics.

*Squatina leae* sp. nov. differs from *S. africana* in its dorsal coloration, which is conspicuously bright, beige to light grayish-brown, with many light yellowish flecks on trunk, and pectoral and pelvic fins. It also has countless densely set, minute dark spots, partially forming pseudocelli, over the dorsal surface. By contrast, *S. africana* is medium to dark brown, reddish-brown, or grayish, with a highly variable pattern of numerous light and, particularly, dark reddish spots and blotches, many of which are granular-centered ocelli or pseudocelli; the spots and blotches are marbled with brownish reticulations and the dark blotches partially form symmetrical dark bands or saddles, particularly in juveniles. The new species is also smaller than *S. africana*, with a size at birth of 180–190 mm TL (vs. 240–340 mm TL), and maturity size of males between 373 and 645 mm TL, with detailed size range unknown (vs. 640–950 mm TL, with size varying greatly between sources), maturity size of females between 562 and 713 mm TL, with detailed size range unknown (vs. 700–1070 mm TL, with size varying greatly between sources), and maximum size 870 mm TL (vs. 1220 or possibly 1300 mm TL). Taxonomical differences include the morphology of the pectoral skeleton (four pectoral radials articulating with the propterygium in the new species vs. three in *S. africana*); posterior nasal flap with (vs. without) an additional barblet; denticle arrangement and morphology, particularly adult males with proportionally larger enlarged midline denticles, more posterior origin of ventral tail denticles, and more vertical ridges (>10 vs. four) on enlarged dorsal head denticles, as well as differences in denticle coverages in juveniles; fewer monospondylous (43–46 vs. 46–49), diplospondylous precaudal (55–58 vs. 58–62), and total precaudal (100–104 vs. 104–111) vertebral centra; trunk width (18.3–25.1 vs. 16.1–17.9% TL); pectoral-pelvic space (10.0–14.9 vs. 6.0–10.2% TL); distal tip of pectoral fin not or only slightly vs. clearly overlapping pelvic fin; clasper size (inner length 18.4–20.4 vs. 14.6% TL, outer length 6.1 vs. 4.1% TL, base width 2.7–3.4 vs. 1.6% TL); and several morphometrics only applying to juveniles. The presence of a propterygium articulating with four elements in *S. leae* sp. nov. is a characteristic that has not yet been found in any other species of *Squatina*, making it a unique diagnostic feature and a possible autapomorphy of the new species (Figure 31).

***Conservation implications***—Angel sharks are among the most threatened groups of cartilaginous fishes globally [16,75,76,77]. This is a consequence of their life history characteristics of slow growth, late age at maturity, low fecundity, their demersal habitat, and the fact that they live in coastal areas (<300 m depth) that are especially vulnerable to habitat degradation and overfishing from trawling and gill-netting [12,16,77]. The collapse of the populations of all three eastern Atlantic species of angel sharks is perhaps the best-known example for the susceptibility of angel sharks to overfishing and habitat loss, with all three species having been extirpated from much of their ranges [77,78,79,80]. Accordingly, 13 of 22 species assessed were categorized as being “threatened” (eight Critically Endangered, four Endangered, one Vulnerable), while only five were assessed as Least Concern, three as Near Threatened, and one as Data Deficient based on the International Union for Conservation of Nature (IUCN) Red List of Threatened Species categories [81]. A lack of taxonomic clarity and species-specific identification has further compounded conservation and fisheries management efforts.

The implications for conservation and management concerns surrounding the newly described angel shark species are considerable. First, intense unregulated small-scale fisheries likely catch angel sharks as bycatch operating across most of the range of the African angel shark *sensu lato* [82]. This has led to a strong decline in *Squatina africana* off Zanzibar, where this species was reportedly the most common elasmobranch species present in small-scale landings according to fisher interviews in the mid-1990s, reported by 96% of fishers [60]. Later work examining small-scale landings in Zanzibar has not found any individuals [83,84]. Similarly, a recent 12-month survey monitoring 21 landing sites across Kenya, Zanzibar, and northern Madagascar did not record any individuals of *S. africana* [85]. Presently, there are no management or conservation measures in place for sharks and rays in most areas of the southwestern Indian Ocean [86,87] and many species are now threatened due to targeted shark and ray fisheries and insufficient fisheries management and enforcement [88]. While fisheries management and enforcement have improved in South Africa over recent decades, drastic improvements are urgently needed elsewhere [88]. Strict protection of Endangered and Critically Endangered species and sustainable management of Vulnerable, Near Threatened, and Least Concern species are needed to ensure robust shark and ray populations and maintain ecosystem functionality, supported by species-level data collection and reductions in incidental catch [88]. In the northwestern Indian Ocean, significant population declines in many pelagic shark species are thought to have been caused by intensive pelagic fishing [89]. In the Indian Exclusive Economic Zone (EEZ), e.g., the catch per unit effort of pelagic sharks has declined by 96% between 1991 and 2006 [90], highlighting the need for urgent conservation and management measures [89]. Due to declines off Zanzibar and the lack of management and conservation measures in small-scale fisheries across most of its range, it has been suspected that *S. africana* has undergone a decline of at least 20 to 30% over three generation lengths [82]. Second, the distribution of *S. africana* is more restrictive than previously thought; it now seems to only occur off Madagascar and along the east coast of Africa, with the northern range limit unknown and apparently neither including oceanic banks and plateaus nor the northern Indian Ocean (present study). *Squatina africana* has been assessed as Near Threatened by the IUCN Red List of Threatened Species [82], but with its distribution now being more restrictive than previously thought, management and conservation policies need to be reviewed. Third, the biology of the new species is almost completely unknown. Although *S. leae* sp. nov. has been found at different locations, its known distribution is highly disrupted and patchy. Furthermore, nothing is known about its life history, population size, or the number of suitable habitats within its geographic distribution. Correspondingly, the conservation concerns surrounding *S. africana* and *S. leae* sp. nov. are serious and significant improvements in fisheries management and enforcement are urgently needed to ensure sustainable populations of angel sharks in the Indian Ocean.

**Key to the six valid species of *Squatina* in the Indian Ocean (modified after Last and White** [22]**):**

1. Anterior nasal flaps with simple, unfringed barbels and slightly fringed to smooth posterior margins ………………………………………………………………………………...2
  -Anterior nasal flaps with strongly fringed, multibranched barbels ………………………………………………………………………………..4
2. Dorsal surface almost uniformly dark greyish brown or with dark brown blotches and ocelli, as well as characteristic dark brown subdorsal saddles; maximum size 1341 mm TL ………………………………………………………...*Squatina legnota* (Indonesia)
  -Dorsal surface bright beige to light grayish-brown or medium to dark brown with numerous light and dark reddish spots and blotches marbled with brownish reticulations; maximum size 870–1220 mm TL ………………………………………………………………………………..3
3. Dorsal surface medium to dark brown with numerous light and dark reddish spots and blotches marbled with brownish reticulations; distal tip of pectoral fin clearly overlapping pelvic fin; posterior nasal flap without additional barblet; maturity size of males ~810–840 mm TL, maximum size 1220 (possibly 1300) mm TL …………………………………………*Squatina africana* (South Africa, Mozambique, Madagascar)
  -Dorsal surface bright beige to light grayish-brown; distal tip of pectoral fin not or only slightly overlapping pelvic fin; posterior nasal flap with an additional barblet; maturity size of males less than 645 mm TL, maximum size 870 mm TL ………………………………………………*Squatina leae* sp. nov. (Mascarene Plateau, southwestern India)
4. Interorbital space flat or convex; dorsal surface grayish with white flecks; no raised protuberances (enlarged thorns or scutes) around eyes; lower lobe of caudal fin with numerous dark spots…………………………………*Squatina australis* (southern Australia)
  -Interorbital space moderately to strongly concave; dorsal surface with white or bluish spots, or ocelli; raised protuberances (enlarged thorns or scutes) present around eyes; caudal fin with mainly pale spots …………………………………………………………………………5
5. Body with 3 pairs of large granular ocelli near anterior bases of pectoral (2) and pelvic (1) fins; no row of enlarged, scute-like denticles along midline of trunk and tail …………………………………………………………..*Squatina tergocellata* (southwestern Australia)
  -Dorsal surfaces without large ocelli near bases of pectoral and pelvic fins but with numerous grayish blue spots; a single row of enlarged, scute-like denticles along midline of trunk and tail …………………………………*Squatina pseudocellata* (northwestern Australia)

## 5. Conclusions

The recognition of a new species, *Squatina leae* sp. nov., with the redescription of *S. africana*, clarifies the taxonomic status and distribution of these two western Indian Ocean angel shark species. This is essential for improved data collection and research and for more effective conservation and management policy decisions. Accordingly, this information must be incorporated into future conservation and management plans of sharks in the western Indian Ocean. The current lack of conservation plans at all scales in this ocean area, as well as the need for more research, will likely jeopardize the populations of western Indian Ocean angel sharks in the future.

## 6. Comparative Material Examined

*Squatina aculeata* (1 specimen): MNHN 1979.133, adult (head only), Eastern Atlantic Ocean, continental shelf of Tunisia.

*Squatina argentina* (8 specimens): MCP 3754, juvenile female (392 mm TL), continental slope between Uruguay and Argentina; MOFURG 434, juvenile female (334 mm TL), continental shelf of Rio Grande county, Rio Grande do Sul state, Brazil; MOFURG 457, juvenile male (359 mm TL), same data as MOFURG 434; MNHN 1984.33, juvenile female (392 mm TL), continental shelf of Uruguay, 35°6′ S, 52°34′1″ W; MZUSP 73170, neonate female (280 mm TL), continental shelf of Santa Catarina state, Brazil, 28°53′ S, 48°35′ W; UERJ 794, adult male (1225 mm TL), Rio Grande do Sul state, Brazil, skeleton; UERJ 795, head only, no locality data, skeleton; UERJ 975, juvenile male (646 mm TL), continental shelf of Rio Grande do Sul state, Brazil, 31°24′ S, 50°41′ W.

*Squatina david* (4 specimens): MCZ 167664, juvenile male, 480 mm TL, 10°45′ N, 66°37′ W, 228 m; NSMT-P 40633, juvenile male, 438 mm TL, 6°43′ N, 52°39′ W, 172–174 m; UF 101312, juvenile female, 312 mm TL, northeast of Paramaribo, Surinam, Atlantic Ocean, 7° N, 54° W, 51.84 m; USNM 400796, juvenile male, 325 mm TL, Panama, 9°15′54″ N, 81°40′54″ W, 260–272 m.

*Squatina dumeril* (11 specimens): MNHN A-9692, lectotype, adult male, 1172 mm TL, off coast of New York state, USA, 40°N, 67°W; MCZ 38708, juvenile male, 347 mm TL, 27°3′ N, 84°16′ W, 137 m; MCZ 51029, juvenile female, 302 mm TL, 11°8′30″ N, 74°29′ W, 180–220 m; MCZ 51035, juvenile male, 311 mm TL, 11°6′42″ N, 74°30′ W, 180 m; MCZ 170737, juvenile female, 377 mm TL, 36°18′ N, 75°34′ W; MCZ 170748, juvenile male, 292 mm TL, Atlantic Ocean, 35°40′ N, 74°48′ W, 189 m; USNM 157746, juvenile female, 305 mm TL, off Mississippi Delta, Louisiana, USA, 28°45′ N, 89°43′ W, 91 m depth; USNM 193583, juvenile female, 302 mm TL, 28°15′ N, 90°7′ W, 110 m; USNM 358610, juvenile male, 278 mm SL, Alabama, USA, Gulf of Mexico, 29°21′11″–29°20′59″ N, 87°43′44″–87°44′08″ W, 107 m depth; USNM 410797, juvenile female, 330 mm TL, North Carolina, USA, 36°16′12″ N, 75°32′12″ W; USNM 410798, juvenile male, 320 mm TL, North Carolina, USA, 36°15′53″ N, 75°33′45″ W.

*Squatina guggenheim* (196 specimens): MCP 2222, juvenile female (437 mm TL), Rio Grande county, Rio Grande do Sul state, Brazil, 32°11′ S, 52°07′ W; MCP 3497, neonate male (252 mm TL), Garopaba county, Santa Catarina state; MCP 3755, juvenile male (385 mm TL), continental shelf of Rio Grande do sul state between Rio Grande county and North of Cabo Polônio, Uruguay; MCP 4648, juvenile female (428 mm TL), praia do Bojuru, Rio Grande do Sul state, Brazil, 31°40′ S, 51°20′ W; MCP 4712, adult male (730 mm TL), praia do Cassino, Rio Grande do Sul state, Brazil, 32°14′ S, 52°07′ W; MCP 4713, subadult female (753 mm TL), same data as MCP 4712; MCP 4720, juvenile male (415 mm TL), same data as MCP 4712; MCP 4721, juvenile female (427 mm TL), same data as MCP 4712; MCP 4726, juvenile female (372 mm TL), praia do Quintão, Rio Grande county, Rio Grande do Sul state, Brazil, 30° 24′ S, 50°17′ W; MCP 4727, juvenile male (397 mm TL), same data as MCP 4712; MCP 4733, juvenile male (405 mm TL), same data as MCP 4712; MCP 4734, juvenile female (430 mm TL), same data as MCP 4712; MCP 4735, subadult female (675 mm TL), same data as MCP 4712; MCP 4751, adult female (788 mm TL), same data as MCP 4726; MCP 4752, juvenile female (353 mm TL), same data as MCP 4712; MCP 4815, juvenile male (372 mm TL), same data as MCP 4712; MCP 5482, juvenile male (455 mm TL), same data as MCP 4726; MCP 6123, juvenile male (401 mm TL), same data as MCP 4726; MCP 6124, juvenile male (362 mm TL), same data as MCP 4726; MCP 6125, juvenile female (376 mm TL), same data as MCP 4726; MCP 6189, juvenile male (398 mm TL), same data as MCP 4712; MCP 6126, juvenile male (372 mm TL), same data as MCP 4726; MCP 7125, juvenile male (460 mm TL), near to Torres county, Rio Grande do Sul state, Brazil, 29°20′ S, 49°34′ W; MCP 7446, juvenile male (358 mm TL), between Farol da Solidão and Farol da Conceição, Mostardas County, Rio Grande do Sul state, Brazil, 31°05′ S, 50°40′ W; MCP 14041, juvenile male (500 mm TL), Rio Grande county, continental shelf of Rio Grande do Sul state, Brazil; MCP 17432, juvenil female (467 mm TL), baia de Sepetiba, Rio de Janeiro state, Brazil, 23°S, 43°45′ W; MNHN 1984.32, juvenile female (519 mm TL), continental shelf of Uruguay, 34°43′1″ S, 54°3′ W; MNHN 1989.134, juvenile female (558 mm TL), continental shelf of Uruguay, 34°57′ S, 55°16′59″ W; MNHN 2005.1747, juvenile male (257 mm TL), continental shelf of Rio de Janeiro state, Brazil, 23°S, 43°16′59″ W; MNRJ 14, adult female (920 mm TL), no locality data; MNRJ 8960, subadult female (690 mm TL), Barra de Guaratiba, Rio de Janeiro state, Brazil, 23°07′ S, 43°33′ W; MNRJ 32534, juvenile male (468 mm TL), continental shelf of Rio de Janeiro state, Brazil; MNRJ 32535, juvenile female (523 mm TL), continental shelf of Rio de Janeiro state, Brazil; MOFURG 19, adult male (773 mm TL), continental shelf of Rio Grande county, Rio Grande do Sul state, Brazil; MOFURG 181, six embryos (225 mm TL), same data as MOFURG 19; MOFURG 182, nine embryos (220 mm TL), same data as MOFURG 19; MOFURG 183, seven embryos (220 mm TL), same data as MOFURG 19; MOFURG 184, five embryos (183 mm TL), same data as MOFURG 19; MOFURG 185, 19 embryos (130 mm TL), same data as MOFURG 19; MOFURG 270, juvenile female (418 mm TL), same data as MOFURG 19; MOFURG 419, juvenile male (298 mm TL), same data as MOFURG 19; MOFURG 464, juvenile female (375 mm TL), same data as MOFURG 19; MOFURG 473, embryo female (238 mm TL), same data as MOFURG 19; MOFURG 501, juvenile female (393 mm TL), same data as MOFURG 19; MOFURG 528, juvenile male (666 mm TL), same data as MOFURG 19; MOFURG 576, juvenile female (330 mm TL), same data as MOFURG 19; MOFURG 581, juvenile female (351 mm TL), same data as MOFURG 19; MOFURG 592, juvenile female (623 mm TL), same data as MOFURG 19; MOFURG 78.0014, juvenile female (398 mm TL), same data as MOFURG 19; MOFURG 83.0722, juvenile female (600 mm TL), same data as MOFURG 19; MOFURG 83.0740, juvenile female (385 mm TL), same data as MOFURG 19; MOFURG 83.0742, juvenile female (376 mm TL), same data as MOFURG 19; MZUSP 9981, juvenile male (471 mm TL), continental shelf of Rio Grande do Sul state, Brazil; MZUSP 10602.2, juvenile male (664 mm TL), continental shelf of Rio Grande do Sul state, Brazil, 31°13′ S, 50°35′ W; MZUSP 43101, juvenile female (631 mm TL), continental shelf of São Paulo state, Brazil, 23°56′ S, 45°08′ W; MZUSP 43102, adult female (846 mm TL), same data as MZUSP 43101; MZUSP 47680, four specimens, juveniles (350 mm TL), continental shelf of Rio Grande do Sul state, Brazil, 31°09′ S, 50°43′ W; MZUSP 73173, neonate female (216 mm TL), continental shelf of São Paulo state, Brazil, 24°1′ S, 45°55′ W; MZUSP 73176.A, juvenile male (536 mm TL), continental shelf of Rio Grande do Sul state, Brazil, 31°13′ S, 50°35′ W; MZUSP 73176.B, juvenile male (528 mm TL), same data as MZUSP 73176.A; MZUSP 73176.C, juvenile female (346 mm TL), same data as MZUSP 73176.A; MZUSP 73182.A, juvenile female (491 mm TL), continental shelf of Rio grande do Sul state, Brazil, 33°58′ S, 52°50′ W; MZUSP 73182.B, juvenile male (501 mm TL), same data as MZUSP 73182.A; MZUSP 73182.C, juvenile male (318 mm TL), same data as MZUSP 73182.A; MZUSP 73184, juvenile male (331 mm TL), continental shelf of Rio Grande do Sul state, Brazil, 31°27′ S, 51°26′ W; MZUSP 73185, juvenile female (346 mm TL), continental shelf of Rio Grande do Sul state, Brazil, 29°43′ S, 49°55′ W; MZUSP 73186.A, juvenile male (444 mm LT), continental shelf of Rio de Janeiro state, Brazil, 23°16′ S, 44°25′ W; MZUSP 73186.B, juvenile male (334 mm TL), same data as MZUSP 73186.A; MZUSP 73186.C, juvenile female (310 mm TL), same data as MZUSP 73186.A; MZUSP 73186.D, juvenile male (288 mm TL), same data as MZUSP 73186.A; MZUSP 73186.E, juvenile male (272 mm TL), same data as MZUSP 73186.A; MZUSP 110867, juvenile female (421 mm TL), continental shelf of southeastern Brazil; MZUSP 110868, juvenile female (496 mm TL), same data as MZUSP 110867; MZUSP 110869, juvenile male (597 mm TL), 3 km in South direction from Ilha dos Gatos, São Sebastião county, São Paulo state, Brazil, 23°48′60“S, 45°41′00“W; MZUSP 110870, juvenile female (449 mm TL), same data as MZUSP 110867; MZUSP 110871, juvenile female (458 mm TL), same data as MZUSP 110867, MZUSP 110872, juvenile male (400 mm TL), same data as MZUSP 110867; MZUSP 111961, adult, (head only), no locality data; NUPEC 0127, juvenile male (384 mm TL), continental shelf of Santa Catarina state, Brazil, 26°55′ S, 48°25′ W; NUPEC 0128, juvenile female (423 mm TL), Ilha do Bom Abrigo, São Paulo state, Brazil, 25°08′ S, 47°49′ W; NUPEC 0135, juvenile female (364 mm TL), Paranaguá county, Paraná state, Brazil, 25°32′ S, 48°13′ W; NUPEC 0139, juvenile female (456 mm TL), Rio Grande county, Rio Grande do Sul state, Brazil, 32°06′ S, 51°58′ W; NUPEC 0145, juvenile male (351 mm TL), Western South Atlantic, between Santos county and Ilha do Bom Abrigo, São Paulo state, Brazil; NUPEC 0160, juvenile male (539 mm TL), same data as NUPEC 0128; NUPEC 1008, juvenile male (285mm TL), Ilhabela county, São Paulo state, 24°S, 45°15′ W; NUPEC 1012, juvenile female (362 mm TL), Ilha da Vitória, São Paulo state, Brazil, 23°47′ S, 44°58′ W; NUPEC 1074, juvenile female (527 mm TL), Guaratiba county, Rio de Janeiro state, Brazil, 23°07′ S, 43°33′ W; NUPEC 1102, juvenile male (294 mm TL), same data as NUPEC 1012; NUPEC 1115, preadult male (781 mm TL), Laje de Santos, São Paulo state, Brazil, 24°20′60“S, 46°10′60“W; NUPEC 1118, juvenile male (255 mm TL), Santos county, São Paulo state, Brazil, 24°05′ S, 46°20′ W; NUPEC 1136, juvenile female (290 mm TL), no locality data; NUPEC 1143, juvenile female (321 mm TL), same data as NUPEC 1143; NUPEC 1158, juvenile female (344 mm TL), Ilha da Queimada Grande, São Paulo state, Brazil, 24°30′ S, 46°38′ W; NUPEC 1174, juvenile female (593 mm TL), same data as NUPEC 1115; NUPEC 1181, juvenile female (363 mm TL), same data as NUPEC 1118; NUPEC 1212, juvenile male (369 mm TL), same data as NUPEC 1118; NUPEC 1214, juvenile male (425 mm TL), same data as NUPEC 1118; NUPEC 1224, juvenile male (337 mm TL), same data as NUPEC 1012; NUPEC 1225, juvenile male (682 mm TL), same data as NUPEC 1012; NUPEC1251, juvenile female (373 mm TL), no locality data; NUPEC 1263, juvenile male (384 mm TL), same data as NUPEC 1012; NUPEC 1267, juvenile female (362 mm TL), same data as NUPEC 1012; NUPEC 1269, juvenile female (372 mm TL), same data as NUPEC 1118; NUPEC 1271, juvenile male (384 mm TL), same data as NUPEC 1118; NUPEC 1295, juvenile male (370 mm TL), same data as NUPEC 1118; NUPEC 1298, juvenile male (456 mm TL), same data as NUPEC 1012; NUPEC 1329, juvenile female (330 mm TL), Ilha do Arvoredo, Santa Catarina state, Brazil, 27°19′ S, 48°20′ W; NUPEC 1338, juvenile female (428 mm TL), same data as NUPEC 1118; NUPEC 1344, juvenile male (337 mm TL), no locality data; NUPEC 1347, juvenile male (423 mm TL), same data as NUPEC 1012; NUPEC 1463, juvenile male (482 mm TL), same data as NUPEC 1118; NUPEC 1464, juvenile male (350 mm TL), no locality data; NUPEC 1481, neonate male (233 mm TL), no locality data; NUPEC 1498, juvenile female (366 mm TL), same data as NUPEC 1118; NUPEC 1583.2, juvenile female (258 mm TL), same data as NUPEC 1329; NUPEC 1583.3, juvenile female (261 mm TL), same data as NUPEC 1329; NUPEC 1583.4, juvenile male (260 mm TL), same data as NUPEC 1329; NUPEC 1598, juvenile female (632 mm TL), no locality data; NUPEC 1602, juvenile female (584 mm TL), Itanhaém county, São Paulo state, Brazil 23°13′ S, 46°45′ W; NUPEC 1603, juvenile male (285 mm TL), same data as NUPEC 1602; NUPEC 1604, juvenile male (324 mm TL), same data as NUPEC 1602; NUPEC 1606, juvenile male (561 mm TL), same data as NUPEC 1602; NUPEC 1609, preadult female (673 mm TL), same data as NUPEC 1602; NUPEC 1649, adult male (872 mm TL), continental shelf of southeastern Brazil; NUPEC 1859, juvenile female (430 mm TL), same data as NUPEC 1329; NUPEC 1525, juvenile female (317 mm TL), Juréia, Peruíbe county, São Paulo state, Brazil, 24°27′ S, 46°59′ W; NUPEC P 20, adult female (954 mm TL), no locality data; UERJ 341, adult female (730 mm TL), Barra de Guaratiba, Rio de Janeiro state, Brazil, 23°07′ S 43°33′ W, skeleton; UERJ 363, adult male (800 mm TL), same data as UERJ 341, skeleton; UERJ 367, juvenile female (620 mm TL), same data as UERJ 341, skeleton; UERJ 382, juvenile male (623 mm TL), Itajaí county, Santa Catarina state, Brazil, 26°55′ S, 48°25′ W; UERJ 383, juvenile male (549 mm TL), same data as UERJ 382; UERJ 384, juvenile female (560 mm TL), same data as UERJ 382; UERJ 385, juvenile male (526 mm TL), same data as UERJ 382; UERJ 386, juvenile male (534 mm TL), same data as UERJ 382; UERJ 388, juvenile female (523 mm TL), same data as UERJ 382; UERJ 481, adult female (932 mm TL), same data as UERJ 341, skeleton; UERJ 654.2, neonate female (215 mm TL), Ilhas Tijucas, Rio de Janeiro state, Brazil, 23°02′ S, 43°18′ W; UERJ 654.3, juvenile female (262 mm TL), same data as UERJ 654.2; UERJ 654.4, juvenile female (478 mm TL), same data as UERJ 654.2; UERJ 654.5, juvenile male (330 mm TL), same data as UERJ 654.2; UERJ 831.1, juvenile male (263 mm TL), Santos county, São Paulo state, Brazil, 24°05′ S 46°20′ W; UERJ 831.2, juvenile male (266 mm TL), same data as UERJ 831.1; UERJ 831.3, juvenile male (358 mm TL), same data as UERJ 831.1; UERJ 831.4, neonate female (225 mm TL), same data as UERJ 831.1; UERJ 882.1, juvenile female (257 mm TL), no locality data; UERJ 882.2, juvenile female (275 mm TL), no locality data; UERJ 882.3, juvenile male (267 mm TL), no locality data; UERJ 882.4, juvenile female (252 mm TL), no locality data; UERJ 882.6, juvenile male (346 mm TL), no locality data; UERJ 882.7, juvenile male (345 mm TL), no locality data; UERJ 882.8, embryo female (200 mm TL), no locality data; UERJ 882.9, juvenile female (274 mm TL), no locality data; UERJ 791, adult female (735 mm TL), same data as UERJ 341, skeleton; UERJ 1572.1, juvenile female (253 mm TL), same data as UERJ 341; UERJ 1572.2, juvenile male (255 mm TL), same data as UERJ 1572.1 UERJ 1599, juvenile female (532 mm TL), same data as UERJ 1572; UERJ 2013, adult female (798 mm TL), Pontal da Barra, Recreio dos Bandeirantes, Rio de Janeiro county, Rio de Janeiro state, Brazil, 23°02′ S, 43°27′ W; UERJ 2183, juvenile male (651 mm TL), Itanhaém county, São Paulo state, Brazil, 23°13′ S, 46°45′ W; UERJ AC 537, neonate male (269 mm TL), Marambaia, Ilha Grande, Rio de Janeiro state, Brazil, 23°13′ S, 44°14′ W; UERJ AC 866, two embryos (220 mm TL), continental shelf of Rio Grande do Sul state.

*Squatina occulta* (80 specimens): **Paratypes**—(2 specimens) MCP 13999, preadult female (903 mm TL), continental shelf of Rio Grande do Sul state, 34°02′ S, 52°19′ W; MZUSP 41518, preadult male (975 mm TL), continental shelf of Rio Grande do Sul state, Brazil, 33°28′ S, 52°01′ W. **Non-type material**—(78 specimens) MNHN 1989.133, juvenile male (630 mm TL), continental shelf of Uruguay, 34°43′1″ S, 54°3′ W; MNRJ 25, adult female (1223 mm TL), no locality data; MNRJ 544, adult male (321 mm TL), continental shelf of Rio de Janeiro state, Brazil, 23°10′ S, 41°50′ W; MNRJ 572.A, embryo male (303 mm TL), Ilha Rasa, Rio de Janeiro state, Brazil, 23°05′ S, 43°33′ W; MNRJ 15458.A, juvenile female (349 mm TL), no locality data; MNRJ 15458.B, juvenile female (318 mm TL), no locality data; MNRJ 15458.C, juvenile male (321 mm), no locality data; MNRJ 30191, juvenile male (808 mm TL), continental shelf of Espírito Santo state, Brazil 19°41′97″ S, 39°23′66″ W; MNRJ 33234, juvenile male (353,5 mm TL), continental shelf of Rio de Janeiro state, Brazil, 23°10′ S, 41°50′ W; MNRJ 32235, juvenile female (357 mm TL), continental shelf of Rio de Janeiro state, Brazil, 23°10′ S, 41°50′ W; MOFURG 178, seven embryos (300 mm TL), continental shelf of Rio Grande county, Rio Grande do Sul state, Brazil; MOFURG 179, three embryos (275 mm TL), same data as MOFURG 178; MOFURG 180, six embryos (190 mm TL), same data as MOFURG 178; MOFURG 186, eight embryos (164 mm TL), same data as MOFURG 178; MOFURG 187, five embryos (275 mm TL), same data as MOFURG 178; MOFURG 424, embryo (238 mm TL), same data as MOFURG 178; MOFURG 485, embryo (230 mm TL), same data as MOFURG 178; MOFURG 494, juvenile male (352 mm TL), same data as MOFURG 178; MOFURG 545, juvenile female (419 mm TL), same data as MOFURG 178; MZUSP 10602.1, juvenile male (613 mm TL), Rio Grande do Sul state, Brazil, 31°13′ S, 50°35′ W; MZUSP 42851, juvenile male (516 mm TL), continental shelf of Rio Grande do Sul state, Brazil, 28°43′ S, 048°20′ W; MZUSP 73171, seven embryos, continental shelf of São Paulo state, Brazil, 24°1′ S, 45°55′ W; MZUSP 73192, adult (head only), continental shelf of Rio Grande do Sul state, Brazil, 32°07′ S, 51°04′ W; MZUSP 110866, juvenile male (426 mm TL), continental shelf of southeastern Brazil; MZUSP 111960, juvenile male (388 mm TL), same data as MZUSP 110866; NUPEC 0147, juvenile male (515 mm TL), Ilha do Bom Abrigo, São Paulo state, Brazil, 25°08′ S, 47°49′ W; NUPEC 1106, juvenile male (315 mm TL), Guaratiba county, Rio de Janeiro state, Brazil, 23°07′ S, 43°33′ W; NUPEC 1128, juvenile male (388 mm TL), Ilha da Vitória, São Paulo state, Brazil, 23°47′ S, 44°58′ W; NUPEC 1213, juvenile female (496 mm TL), Santos county, São Paulo, 24°05′ S, 46°20′ W; NUPEC 1227, juvenile male (569 mm TL), same data as NUPEC 1128; NUPEC 1255, juvenile male (366 mm TL), same data as NUPEC 1128; NUPEC 1259, juvenile male (366 mm TL), same data as NUPEC 1128; NUPEC 1275, juvenile female (362 mm TL), same data as NUPEC 1128; NUPEC 1461.1, juvenile male (286 mm TL), Santos county, São Paulo state, Brazil, 24°05′ S, 46°20′ W; NUPEC 1461.5, juvenile female (275 mm TL), same data as NUPEC 1461.1; NUPEC 1651, juvenile male (743 mm TL), continental shelf of southeastern Brazil; NUPEC 1857, juvenile male (431 mm TL), no locality data; NUPEC 2192, (448 mm TL), juvenile male, Western South Atlantic, continental shelf of southeastern Brazil; NPM 1547, juvenile male (343 mm TL), continental shelf of Rio de Janeiro state, Brazil, 23° 14′ 45“S, 41°22′09“W; UERJ 0387, juvenile female (447 mm TL), Itajaí county, Santa Catarina state, Brazil, 26°55′ S, 48°25′ W; UERJ 0389, juvenile female (393 mm TL), same data as UERJ 387; UERJ 886, juvenile female (292 mm TL), no locality data; UERJ 1099, juvenile male (537 mm TL), Rio Grande do Sul state, Brazil, 31°24′ S, 50°41′ W; UERJ 1600, juvenile male (428 mm TL), continental shelf of southeastern Brazil; UERJ 1658, juvenile male (370 mm TL), same data as UERJ 387; UERJ 1673, juvenile female (416 mm TL), Ilhas Tijuca, Rio de Janeiro state, Brazil, 23°02′ S, 43°18′ W; UERJ AC 514, embryo male (186 mm TL), same data as UERJ 387.

*Squatina oculata* (6 specimens): BMNH 1914.11.2.1, female (790 mm TL), off Lagos, Nigeria, presented by J. Cadman, Western Fisheries Ltd. (photographs); SW 01-2012, stuffed adult male, 1400 mm TL; USNM 220031, juvenile female (370 mm TL), off Liberia, 5°18′ N, 9°54′30″ W; USNM 220032, juvenile female (390 mm TL), off Liberia, 5°24′30″ N, 9°49′ W; ZMH 105062 (ex ISH 2-1967), one juvenile female and one juvenile male, R.V. ‘Walther Herwig’, station 6/67, off South Africa, 09°40′ S, 12°58′ E, 100 m depth, 140′ bottom trawl, 28 April 1967.

*Squatina* sp. (2 specimens): SW uncatalogued, one juvenile stuffed specimen, off Philippines; SW 01-2011, one adult stuffed specimen, 1300 mm TL, off Philippines.

*Squatina* sp. NW1 (3 specimens): MCZ 40791, adult male, 1156 mm TL, Atlantic Ocean, 39°55′ N, 71°45′ W, 133 m; MNHN A-9691, adult male, 1090 mm TL, USA, New York, New York state, 40°40′1″ S, 73°49′59″ W; USNM 118461, juvenile female, 390 mm TL, 39°42′ N, 71°17′ W, 1289 m.

*Squatina squatina* (3 specimens). MNRJ 30997, juvenile female (284 mm TL), Prov. West-Vlaanderen, Blankenberge, De Wenduyne a Zeebrugge, Belgium; UNSM 48351, neonate female (230 mm TL), off Italy; SW 01-1974, North Sea, Zeebrugge, Belgium, 1974 (jaws, 154 × 95 mm).

*Squatina varii* (15 specimens): **Holotype**—MNRJ 43106, adult male, 1110 mm TL, continental slope of Espírito Santo state, Brazil, Southwestern Atlantic Ocean, 19°42′54″ S, 39°25′57″ W, 195 m depth, bottom trawl, vessel N/O Thalassa, 30 June 2000. **Paratypes**—(13 specimens) MNRJ 30190, juvenile female, 690 mm TL, continental slope of Bahia state, Brazil, Southwestern Atlantic, 15°42′40.5″ S, 38°37′58.8″ W, 251 m depth, bottom trawl, vessel N/O Thalassa; MNRJ 30191, juvenile male, 808 mm TL, continental shelf of Espírito Santo state, Brazil, Southwestern Atlantic, 19°41′58.1″ S, 39°23′39.8″ W, 354 m depth, bottom trawl, vessel N/O Thalassa; MNRJ 43100, adult male, 1242 mm TL, continental slope of Espírito Santo state, Brazil, Southwestern Atlantic Ocean, 21°23′57″ S, 40°11′18″ W, 666 m depth, bottom trawl, N/O Thalassa, 5 July 2000 (total length taken only); MNRJ 43101, adult male, 1215 mm TL, data as for MNRJ 43100; MNRJ 43102, adult male, 1191 mm TL, data as for MNRJ 43100; MNRJ 43103, adult female, 1255 mm TL, data as for MNRJ 43100 (total length taken only); MNRJ 43105, adult female, 1320 mm TL, data as for MNRJ 43100; MNRJ 43107, adult male, 980 mm TL, continental slope of Bahia state, Brazil, Southwestern Atlantic, 14°22′3″ S, 38°40′12″ W, 394 m depth, bottom trawl, N/O Thalassa, 8 June 2000; MRNJ 43108, adult female, 1084 mm TL, data as for MNRJ 43107; MNRJ 43109, adult female, 988 mm TL, continental slope of Bahia state, Brazil, Southwestern Atlantic, 14°28′58″ S, 38°54′ W, 278 m depth, 10 June 2000, N/O Thalassa; MNRJ 43110, juvenile female, 949 mm TL, continental slope of Bahia state, Brazil, Southwestern Atlantic, 15°42′41″ S, 38°37′18″ W, 251 m depth, bottom trawl, N/O Thalassa, 12 June 2000; MNRJ 43111 (total length taken only), adult female, 985 mm TL, data as for MNRJ 43110; MNRJ 43112, subadult male, 981 mm TL, continental slope of Sergipe state, Brazil, Southwestern Atlantic, 11°14′23″ S, 37°13′58″ W, 487 m depth, bottom trawl, N/O Thalassa, 17 June 2000. **Non-type material**—MNRJ 43104, adult male, 1080 mm TL, continental slope of Espírito Santo state, Brazil, Southwestern Atlantic Ocean, 21°23′57″ S, 40°11′18″ W, 666 m depth, 5 July 2000, N/O Thalassa (total length taken only).

## Figures and Tables

**Table 1:** *Squatina leae* sp. nov., morphometrics. Individual values for the adult male holotype (CMFRI GA. 15.2.5.4), ranges for the male and female paratypes at PMBC, individual values for the three juvenile paratypes at ZMH, as well as ranges for all paratype specimens and means and standard deviations (SD) for all type specimens. Proportional values are expressed as percentages of total length (TL) except for ranges and mean of TL in mm.

	CMFRI GA. 15.2.5.4, Adult Male Holotype		Minimum Male Paratypes at PMBC (*n* = 6)	Maximum Male Paratypes at PMBC (*n* = 6)	Minimum Female Paratypes at PMBC (*n* = 8)	Maximum Female Paratypes at PMBC (*n* = 8)	ZMH 26097, Juvenile Male Paratype	ZMH 26098, Juvenile Male Paratype	ZMH 26099, Juvenile Female Paratype	Minimum Paratypes (*n* = 17)	Maximum Paratypes (*n* = 17)	Mean Holotype and Paratypes (*n* = 18)	SD Holotype and Paratypes (*n* = 18)
	mm	% TL	% TL	% TL	% TL	% TL	% TL	% TL	% TL	% TL	% TL	% TL	% TL
TL, total length	671.0	100.0	645.0	691.0	537.0	800.0	282.6	249.6	250.2	249.6	800.0	615.4	-
PCLV_1, pre-caudal length ventrally to origin of caudal-fin ridge	nr	nr	nr	nr	nr	nr	76.4	76.7	77.3	76.4	77.3	76.8	0.4
PCLV_2, pre-caudal length ventrally to origin of caudal-fin proper	543.2	81.0	82.0	86.0	80.9	86.1	81.8	80.8	81.7	80.8	86.1	82.9	1.7
PCLD, pre-caudal length dorsally	566.7	84.5	83.4	86.9	83.1	85.8	83.6	82.8	83.4	82.8	86.9	84.2	1.0
PD2, pre-second-dorsal-fin length	499.0	74.4	73.0	75.6	73.3	75.0	73.7	73.4	73.9	73.0	75.6	74.0	0.7
PD1, pre-first-dorsal-fin length	417.5	62.2	61.9	63.4	62.7	64.4	63.7	63.8	63.9	61.9	64.4	63.2	0.7
PP2, pre-pelvic length	295.6	44.0	37.1	39.0	38.0	43.0	39.3	42.0	43.2	37.1	43.2	39.8	2.1
PP1, pre-pectoral length	140.6	21.0	17.8	19.1	15.1	20.0	21.8	21.7	22.5	15.1	22.5	18.8	2.0
PG1, pre-branchial length	94.0	14.0	13.7	14.5	13.5	15.3	16.7	16.0	17.4	13.5	17.4	14.6	1.1
PSP, pre-spiracular length	57.5	8.6	8.1	8.5	7.5	8.6	10.0	10.3	10.1	7.5	10.3	8.5	0.8
PEY, pre-ocular length	16.7	2.5	4.2	5.8	4.5	5.6	6.2	6.2	6.5	4.2	6.5	5.1	0.9
POB, pre-orbital length	32.9	4.9	2.5	3.1	2.1	2.9	5.8	5.8	6.0	2.1	6.0	3.3	1.3
HDW, head width at 1st gill slits	129.6	19.3	16.0	19.0	16.7	19.6	20.1	19.7	20.7	16.0	20.7	18.3	1.3
HWEY, orbital head width	79.9	11.9	12.0	13.4	11.2	14.2	15.8	15.3	15.2	11.2	15.8	13.1	1.3
HWSP, spiracular head width	128.6	19.2	18.3	19.1	17.3	20.5	20.7	21.3	22.3	17.3	22.3	19.3	1.3
MOW_1, mouth width, outer jaws	88.4	13.2	12.7	13.5	12.6	14.2	14.7	15.2	15.3	12.6	15.3	13.5	0.8
MOW_2, mouth width, opening only	78.4	11.7	11.5	12.1	10.8	12.8	13.0	13.3	12.9	10.8	13.3	11.9	0.7
HDH, head height at 1st gill slits	53.2	7.9	4.7	6.0	5.2	7.0	7.3	7.1	7.6	4.7	7.6	6.1	1.0
INOI, interorbital distance, integumental	50.9	7.6	7.7	8.3	7.4	8.8	8.8	8.7	9.1	7.4	9.1	8.1	0.5
INOS, interorbital distance, skeletal	48.0	7.2	nm	nm	nm	nm	6.8	6.8	7.2	6.8	7.2	7.0	0.2
EYL, eye diameter	18.6	2.8	2.1	2.6	1.9	2.6	2.8	2.9	2.9	1.9	2.9	2.4	0.3
EYW, eye width	13.0	1.9	1.5	1.8	1.1	1.7	1.8	1.8	1.9	1.1	1.9	1.6	0.2
ESL, eye-spiracle distance	15.5	2.3	1.5	2.1	1.6	2.1	2.2	2.1	2.1	1.5	2.2	1.9	0.2
INW, internarial distance	44.9	6.7	6.2	6.7	6.1	7.4	7.5	7.3	7.6	6.1	7.6	6.7	0.5
INS, interspiracular distance	49.9	7.4	7.3	7.7	7.3	8.2	8.2	8.7	8.7	7.3	8.7	7.7	0.4
SPL, spiracle length	18.2	2.7	2.3	2.9	2.3	3.4	2.7	2.9	2.9	2.3	3.4	2.7	0.2
ING, intergill length	15.5	2.3	2.2	2.9	2.1	2.9	2.4	2.0	1.9	1.9	2.9	2.6	0.3
INGW, intergill width	50.1	7.5	8.0	9.6	8.1	9.3	8.9	9.0	8.8	8.0	9.6	8.7	0.5
IDS, interdorsal distance	54.1	8.1	6.5	7.9	6.8	7.5	6.4	6.3	6.5	6.3	7.9	7.1	0.5
DCS, dorsal-caudal distance (= caudal peduncle length)	43.1	6.4	7.0	7.4	6.3	7.3	6.8	6.7	6.8	6.3	7.4	6.9	0.3
PPS, pectoral-pelvic distance	72.9	10.9	10.0	14.9	11.0	14.2	10.7	10.2	10.3	10.0	14.9	11.7	1.5
POCS_1, pelvic origin-caudal distance to origin of caudal-fin ridge	nr	nr	nr	nr	nr	nr	39.2	39.9	39.0	39.0	39.9	39.3	0.4
POCS_2, pelvic origin-caudal distance to origin of caudal-fin proper	301.6	44.9	44.6	48.3	42.1	47.4	43.7	42.9	42.7	42.1	48.3	45.5	1.7
PCA_1, pelvic insertion-caudal distance to origin of caudal-fin ridge	nr	nr	nr	nr	nr	nr	25.2	24.7	25.4	24.7	25.4	25.1	0.4
PCA_2, pelvic insertion-caudal distance to origin of caudal-fin proper	219.0	32.6	34.9	38.0	31.7	34.5	29.5	28.7	28.9	28.7	38.0	33.6	2.7
WP1, width at pectoral origins	94.3	14.0	13.5	16.0	15.7	18.1	18.0	16.3	17.3	13.5	18.1	16.2	1.3
TRW, trunk width at pectoral insertions	122.7	18.3	18.9	20.4	19.4	25.1	19.8	21.9	20.6	18.9	25.1	20.6	1.6
TAW, tail width at pelvic insertions	74.5	11.1	10.3	14.1	12.4	14.8	10.7	10.2	10.7	10.2	14.8	12.4	1.5
TAH, tail height at pelvic insertions	40.0	6.0	4.5	6.5	3.9	6.8	5.4	5.0	4.8	3.9	6.8	5.5	0.8
P1L, pectoral-fin length	223.5	33.3	31.1	32.4	33.8	35.2	33.7	32.5	33.5	31.1	35.2	33.4	1.4
P1A, pectoral-fin anterior margin	191.6	28.6	27.8	28.8	28.8	31.1	27.6	26.0	26.9	26.0	31.1	29.0	1.5
P1B, pectoral-fin base length	66.3	9.9	10.5	11.2	11.1	12.6	10.9	10.4	10.3	10.3	12.6	11.3	0.8
P1W, pectoral-fin width	137.9	20.5	19.8	21.4	19.7	21.7	16.9	15.7	16.4	15.7	21.7	20.0	1.8
P1I, pectoral-fin inner margin	118.9	17.7	17.1	18.1	17.2	18.7	17.3	16.7	17.0	16.7	18.7	17.7	0.6
P2L, pelvic-fin length	163.9	24.4	23.1	25.3	24.0	25.7	22.6	21.7	22.6	21.7	25.7	24.0	1.1
P2W, pelvic-fin width	119.6	17.8	11.1	14.6	10.8	15.4	13.1	13.0	13.9	10.8	15.4	13.5	1.8
P2I, pelvic-fin inner margin	81.8	12.2	11.4	13.5	10.1	13.0	9.6	9.0	9.9	9.0	13.5	11.6	1.3
D1B, first dorsal-fin base length	26.7	4.0	3.6	4.3	3.0	4.2	3.3	3.3	3.2	3.0	4.3	3.8	0.4
D1A, first dorsal-fin anterior margin	61.8	9.2	8.6	9.7	8.1	9.4	8.2	8.1	7.5	7.5	9.7	8.7	0.5
D1H, first dorsal-fin height	40.0	6.0	5.2	7.6	4.8	6.8	3.9	4.4	4.7	3.9	7.6	5.8	0.9
D1I, first dorsal-fin inner margin	16.0	2.4	2.3	3.2	2.4	3.1	2.6	2.9	3.0	2.3	3.2	2.9	0.3
D2B, second dorsal-fin base length	24.0	3.6	3.1	3.8	2.8	3.7	3.4	3.1	3.1	2.8	3.8	3.4	0.3
D2A, second dorsal-fin anterior margin	56.6	8.4	8.0	9.0	7.5	8.1	7.6	7.6	7.1	7.1	9.0	8.0	0.4
D2H, second dorsal-fin height	35.0	5.2	4.2	6.9	4.4	5.8	4.1	3.9	4.0	3.9	6.9	5.1	0.8
D2I, second dorsal-fin inner margin	17.3	2.6	2.6	3.2	2.5	3.2	3.1	3.7	3.1	2.5	3.7	2.9	0.3
CDM, dorsal caudal-fin margin	90.3	13.5	12.8	14.0	12.0	13.8	13.0	12.7	12.8	12.0	14.0	13.0	0.6
CVM_1, preventral caudal-fin margin from origin of caudal-fin ridge	nr	nr	nr	nr	nr	nr	21.7	21.6	21.5	21.5	21.7	21.6	0.1
CVM_2, preventral caudal-fin margin from origin of caudal-fin proper	129.0	19.2	15.9	18.5	15.1	18.2	17.4	17.9	17.6	15.1	18.5	17.5	1.0
CAH, caudal-fin height	103.0	15.3	11.2	17.4	11.9	16.9	14.1	13.8	13.7	11.2	17.4	14.2	1.8
CLI, clasper inner length	131.0	19.5	18.4	20.4	na	na	10.6	9.9	na	18.4	20.4	19.7	0.8
CLO, clasper outer length	41.2	6.1	nm	nm	na	na	5.4	4.5	na	nm	nm	6.1	-
CLB, clasper base width	22.6	3.4	2.7	3.0	na	na	1.6	1.3	na	2.7	3.0	2.9	0.3
SVL, snout-cloacal length	311.8	46.5	43.5	47.2	44.3	48.8	49.1	49.5	50.0	43.5	50.0	46.2	2.0
VCL, cloacal-caudal length	359.3	53.5	55.2	58.5	53.6	57.3	51.0	50.3	50.2	50.2	58.5	54.9	2.4

Abbreviations: na = not applicable; nm = not measured; nr = no ridge.

**Table 2:** Meristics of *Squatina leae* sp. nov. (*n* = 7), *S. africana* (*n* = 9), and the four congeners from the eastern Indian Ocean (data of the latter four species kindly provided by Peter R. Last and John Pogonoski).

	*S. leae* sp. nov.	*S. leae* sp. nov. Range	*S. leae* sp. nov. Mean	*S. africana*	*S. africana* Range	*S. africana* Mean	*S. australis* Range	*S. legnota* Range	*S. pseudocellata* Range	*S. tergocellata* Range
	Holotype	Paratypes (*n* = 6)	Holotype/ Paratypes (*n* = 7)	Holotype	Non-Types (*n* = 8)	Holotype/ Non-Types (*n* = 9)	*n* = 2	*n* = 2	*n* = 6	*n* = 5
Propterygial radials	4	4	4.0	3	3	3.0	-	-	-	-
Mesopterygial radials	12	12	12.0	12	12–13	12.2	-	-	-	-
Metapterygial radials (approximately)	21	22–24	22.3	26	22–26	24.2	-	-	-	-
Total pectoral radials	37	38–40	38.3	41	37–42	39.4	-	-	-	-
Pelvic radials	28	24–26	25.0	28	25–28	26.3	-	-	-	-
Secondary radials from 1st pelvic radial	4	5	4.8	4	4–6	5.0	-	-	-	-
Monospondylous	45	43–46	45.1	47	46–49	47.9	43	50–51	46–47	48–50
Diplospondylous precaudal	56	55–58	57.0	61	58–62	59.4	49–50	61–63	54–59	53–56
Total precaudal	101	100–104	102.1	108	104–111	107.3	92–93	112–113	101–105	102–105
Diplospondylous caudal	29	31–33	31.4	30	27–32	30.3	30–31	31–32	27–33	25–31
Total vertebrae	130	132–136	133.6	138	134–143	137.6	123	144	130–135	129–135
Upper jaw—right	9	9	9.0	9	9–10	9.3	9–10	9	8	8–10
Upper raw—left	9	9	9.0	9	9–10	9.3	9–10	9	8	9–10
Lower jaw—right	9	9	9.0	9	9–10	9.3	9	9	7–8	8–9
Lower jaw—left	9	9	9.0	10	9–10	9.3	9	9	7–8	8–9

**Table 3:** *Squatina africana*, morphometrics. Individual values for the late subadult male holotype (BMNH 1906.11.19.21) and three juvenile non-type specimens at ZMH, as well as ranges, means, and standard deviations (SD) for all four examined specimens. Proportional values are expressed as percentages of total length (TL) except for ranges and mean of TL in mm.

	BMNH 1906.11.19.21, Late Subadult Male Holotype		ZMH 26100, Juvenile Male		ZMH 25561, Juvenile Male		ZMH 123064, Juvenile Female		Minimum (*n* = 4)	Maximum (*n* = 4)	Mean (*n* = 4)	SD (*n* = 4)
	mm	% TL	mm	% TL	mm	% TL	mm	% TL	% TL	% TL	% TL	% TL
TL, total length	837.0	100.0	446.0	100.0	394.0	100.0	309.0	100.0	309.0	837.0	496.5	-
PCLV_1, pre-caudal length ventrally to origin of caudal-fin ridge	nr	nr	350.0	78.5	305.0	77.4	241.0	78.0	77.4	78.5	78.0	0.5
PCLV_2, pre-caudal length ventrally to origin of caudal-fin proper	710.0	84.8	370.0	83.0	325.0	82.5	257.0	83.2	82.5	84.8	83.4	1.0
PCLD, pre-caudal length dorsally	nm	nm	375.0	84.1	335.0	85.0	260.0	84.1	84.1	85.0	84.4	0.5
PD2, pre-second-dorsal-fin length	610.0	72.9	325.0	72.9	284.2	72.1	228.0	73.8	72.1	73.8	72.9	0.7
PD1, pre-first-dorsal-fin length	525.0	62.7	278.2	62.4	241.1	61.2	195.0	63.1	61.2	63.1	62.3	0.8
PP2, pre-pelvic length	292.0	34.9	176.6	39.6	155.1	39.4	121.3	39.2	34.9	39.6	38.3	2.3
PP1, pre-pectoral length	160.0	19.1	88.1	19.8	74.1	18.8	64.7	20.9	18.8	20.9	19.7	0.9
PG1, pre-branchial length	110.0	13.1	66.1	14.8	59.2	15.0	50.2	16.2	13.1	16.2	14.8	1.3
PSP, pre-spiracular length	67.0	8.0	39.8	8.9	35.8	9.1	29.2	9.5	8.0	9.5	8.9	0.6
PEY, pre-ocular length	45.0	5.4	26.8	6.0	22.8	5.8	19.3	6.3	5.4	6.3	5.9	0.4
POB, pre-orbital length	33.0	3.9	23.1	5.2	21.0	5.3	18.1	5.9	3.9	5.9	5.1	0.8
HDW, head width at 1st gill slits	174.0	20.8	90.2	20.2	75.0	19.0	66.8	21.6	19.0	21.6	20.4	1.1
HWEY, orbital head width	105.0	12.5	65.9	14.8	54.0	13.7	49.4	16.0	12.5	16.0	14.3	1.5
HWSP, spiracular head width	157.0	18.8	91.5	20.5	75.1	19.1	69.1	22.4	18.8	22.4	20.2	1.7
MOW_1, mouth width, outer jaws	nm	nm	62.2	14.0	52.4	13.3	50.8	16.5	13.3	16.5	14.6	1.7
MOW_2, mouth width, opening only	89.0	10.6	52.5	11.8	43.9	11.1	40.7	13.2	10.6	13.2	11.7	1.1
HDH, head height at 1st gill slits	50.0	6.0	30.6	6.9	31.1	7.9	22.4	7.2	6.0	7.9	7.0	0.8
INOI, interorbital distance, integumental	67.0	8.0	37.5	8.4	31.8	8.1	30.6	9.9	8.0	9.9	8.6	0.9
INOS, interorbital distance, skeletal	nm	nm	30.7	6.9	25.3	6.4	23.1	7.5	6.4	7.5	6.9	0.5
EYL, eye diameter	16.0	1.9	11.6	2.6	10.0	2.5	9.4	3.1	1.9	3.1	2.5	0.5
EYW, eye width	10.0	1.2	7.1	1.6	5.8	1.5	5.5	1.8	1.2	1.8	1.5	0.2
ESL, eye-spiracle distance	13.0	1.6	8.4	1.9	7.7	1.9	5.6	1.8	1.6	1.9	1.8	0.2
INW, internarial distance	51.0	6.1	30.9	6.9	25.5	6.5	22.9	7.4	6.1	7.4	6.7	0.6
INS, interspiracular distance	63.0	7.5	33.4	7.5	29.0	7.3	26.4	8.5	7.3	8.5	7.7	0.5
SPL, spiracle length	23.0	2.7	13.1	2.9	9.9	2.5	11.4	3.7	2.5	3.7	3.0	0.5
ING, intergill length	20.0	2.4	8.4	1.9	8.6	2.2	8.4	2.7	1.9	2.7	2.3	0.4
INGW, intergill width	59.0	7.0	36.8	8.3	30.1	7.6	27.0	8.7	7.0	8.7	7.9	0.7
IDS, interdorsal distance	56.0	6.7	30.0	6.7	32.9	8.4	18.0	5.8	5.8	8.4	6.9	1.1
DCS, dorsal-caudal distance (= caudal peduncle length)	65.0	7.8	32.1	7.2	33.9	8.6	22.3	7.2	7.2	8.6	7.7	0.7
PPS, pectoral-pelvic distance	50.0	6.0	43.4	9.7	40.4	10.2	25.6	8.3	6.0	10.2	8.6	1.9
POCS_1, pelvic origin-caudal distance to origin of caudal-fin ridge	nr	nr	180.0	40.4	156.9	39.8	111.6	36.1	36.1	40.4	38.8	2.3
POCS_2, pelvic origin-caudal distance to origin of caudal-fin proper	400.0	47.8	197.3	44.2	176.4	44.8	125.2	40.5	40.5	47.8	44.3	3.0
PCA_1, pelvic insertion-caudal distance to origin of caudal-fin ridge	nr	nr	119.6	26.8	104.8	26.6	73.9	23.9	23.9	26.8	25.8	1.6
PCA_2, pelvic insertion-caudal distance to origin of caudal-fin proper	290.0	34.6	133.8	30.0	123.9	31.4	85.6	27.7	27.7	34.6	30.9	2.9
WP1, width at pectoral origins	164.0	19.6	70.2	15.7	56.2	14.3	57.7	18.7	14.3	19.6	17.1	2.5
TRW, trunk width at pectoral insertions	135.0	16.1	77.9	17.5	63.7	16.2	55.4	17.9	16.1	17.9	16.9	0.9
TAW, tail width at pelvic insertions	99.0	11.8	48.9	11.0	44.1	11.2	30.9	10.0	10.0	11.8	11.0	0.8
TAH, tail height at pelvic insertions	50.0	6.0	23.8	5.3	27.3	6.9	17.5	5.7	5.3	6.9	6.0	0.7
P1L, pectoral-fin length	270.0	32.3	149.9	33.6	125.4	31.8	109.9	35.6	31.8	35.6	33.3	1.7
P1A, pectoral-fin anterior margin	236.0	28.2	128.8	28.9	103.2	26.2	94.6	30.6	26.2	30.6	28.5	1.8
P1B, pectoral-fin base length	100.0	11.9	51.0	11.4	40.5	10.3	40.6	13.1	10.3	13.1	11.7	1.2
P1W, pectoral-fin width	150.0	17.9	76.2	17.1	65.5	16.6	55.0	17.8	16.6	17.9	17.4	0.6
P1I, pectoral-fin inner margin	135.0	16.1	73.1	16.4	66.0	16.7	54.5	17.6	16.1	17.6	16.7	0.7
P2L, pelvic-fin length	196.0	23.4	105.7	23.7	87.4	22.2	71.6	23.2	22.2	23.7	23.1	0.7
P2W, pelvic-fin width	112.0	13.4	59.7	13.4	45.2	11.5	34.8	11.3	11.3	13.4	12.4	1.2
P2I, pelvic-fin inner margin	89.0	10.6	39.4	8.8	34.0	8.6	23.3	7.5	7.5	10.6	8.9	1.3
D1B, first dorsal-fin base length	32.8	3.9	15.9	3.6	14.5	3.7	11.6	3.8	3.6	3.9	3.7	0.2
D1A, first dorsal-fin anterior margin	68.0	8.1	39.1	8.8	32.0	8.1	26.4	8.5	8.1	8.8	8.4	0.3
D1H, first dorsal-fin height	38.0	4.5	25.2	5.7	23.7	6.0	19.9	6.4	4.5	6.4	5.7	0.8
D1I, first dorsal-fin inner margin	22.0	2.6	13.0	2.9	12.2	3.1	10.4	3.4	2.6	3.4	3.0	0.3
D2B, second dorsal-fin base length	29.0	3.5	14.9	3.3	13.7	3.5	11.7	3.8	3.3	3.8	3.5	0.2
D2A, second dorsal-fin anterior margin	65.0	7.8	35.3	7.9	28.3	7.2	24.4	7.9	7.2	7.9	7.7	0.3
D2H, second dorsal-fin height	33.0	3.9	23.3	5.2	20.5	5.2	14.5	4.7	3.9	5.2	4.8	0.6
D2I, second dorsal-fin inner margin	21.0	2.5	14.0	3.1	12.0	3.0	10.2	3.3	2.5	3.3	3.0	0.3
CDM, dorsal caudal-fin margin	101.0	12.1	56.5	12.7	48.5	12.3	37.0	12.0	12.0	12.7	12.2	0.3
CVM_1, preventral caudal-fin margin from origin of caudal-fin ridge	nr	nr	99.0	22.2	88.8	22.5	69.9	22.6	22.2	22.6	22.5	0.2
CVM_2, preventral caudal-fin margin from origin of caudal-fin proper	137.0	16.4	77.7	17.4	69.0	17.5	60.3	19.5	16.4	19.5	17.7	1.3
CAH, caudal-fin height	100.0	11.9	61.9	13.9	53.8	13.6	47.3	15.3	11.9	15.3	13.7	1.4
CLI, clasper inner length	122.0	14.6	43.8	9.8	43.7	11.1	na	na	14.6	14.6	14.6	-
CLO, clasper outer length	34.0	4.1	19.5	4.4	19.2	4.9	na	na	4.1	4.1	4.1	-
CLB, clasper base width	13.0	1.6	7.7	1.7	6.7	1.7	na	na	1.6	1.6	1.6	-
SVL, snout-cloacal length	390.0	46.6	209.9	47.1	183.9	46.7	150.3	48.7	46.6	48.7	47.2	1.0
VCL, cloacal-caudal length	445.0	53.2	235.6	52.8	209.3	53.1	158.1	51.2	51.2	53.2	52.6	0.9

Abbreviations: na = not applicable; nm = not measured; nr = no ridge.

## Data Availability

The data presented in this study are available in this published article.

## References

[1] Ebert D.A., Fowler S., Dando M. (2021). Sharks of the World: A Complete Guide.

[2] Claeson K.M., Hilger A. (2011). Morphology of the anterior vertebral region in elasmobranchs: Special focus, Squatiniformes. Foss. Rec..

[3] Naylor G.J.P., Ryburn J.A., Fedrigo O., López J., Hamlett W.C. (2005). Phylogenetic Relationships among the Major Lineages of Modern Elasmobranchs. Reprodutive Biology and Phylogeny of Chondrichthyes-Sharks, Batoids and Chimaeras.

[4] Naylor G.J.P., Caira J.N., Jensen K., Rosana K.A.M., White W.T., Last P.R. (2012). A DNA sequence–based approach to the identification of shark and ray species and its implications for global elasmobranch diversity and parasitology. Bull. Am. Mus. Nat. Hist..

[5] Naylor G., Caira J., Jensen K., Rosana K., Straube N., Lakner C., Carrier J.C., Musick J.A., Heithaus M.R. (2012). Elasmobranch Phylogeny: A Mitochondrial Estimate Based on 595 Species. Biology of Sharks and Their Relatives.

[6] Shirai S. (1992). Squalean Phylogeny—A New Framework of “Squaloid” Sharks and Related Taxa.

[7] Shirai S., Stiassny M.L.J., Parenti L.R., Johnson G.D. (1996). Phylogenetic Interrelationships of Neoselachians (Chondrichthyes: Euselachii). Interrelationships of Fishes.

[8] de Carvalho M.R., Stiassny M.L.J., Parenti L.R., Johnson G.D. (1996). Higher-Level Elasmobranch Phylogeny, Basal Squaleans, and Paraphyly. Interrelationships of Fishes.

[9] Shirai S. (1992). Phylogenetic Relationships of the Angel Sharks, with Comments on Elasmobranch Phylogeny (Chondrichthyes, Squatinidae). Copeia.

[10] Weigmann S., Stehmann M.F.W., Thiel R. (2014). Contribution to the taxonomy and distribution of *Pristiophorus nancyae* (Elasmobranchii: Pristiophoriformes) from the deep western Indian Ocean. Mar. Biodivers..

[11] Moreira R.A., de Carvalho M.R. (2019). Clasper Morphology of the Japanese Sawshark, *Pristiophorus japonicus* Günther, 1870 (Chondrichthyes: Elasmobranchii). Anat. Rec..

[12] Weigmann S. (2016). Annotated checklist of the living sharks, batoids and chimaeras (Chondrichthyes) of the world, with a focus on biogeographical diversity. J. Fish Biol..

[13] Weigmann S. (2017). Reply to Borsa (2017): Comment on ‘Annotated checklist of the living sharks, batoids and chimaeras (Chondrichthyes) of the world, with a focus on biogeographical diversity by Weigmann (2016)’: Reply to borsa’s comment on weigmann (2016). J. Fish Biol..

[14] Vaz D.F.B., de Carvalho M.R. (2018). New Species of *Squatina* (Squatiniformes: Squatinidae) from Brazil, with Comments on the Taxonomy of Angel Sharks from the Central and Northwestern Atlantic. Copeia.

[15] Bigelow H.B., Schroeder W.C. (1948). Fishes of the Western North Atlantic part one: Sharks.

[16] Ellis J.R., Barker J., McCully Phillips S.R., Meyers E.K.M., Heupel M. (2021). Angel sharks (Squatinidae): A review of biological knowledge and exploitation. J. Fish. Biol..

[17] Compagno L.J.V. (1984). FAO Species Catalogue. Sharks of the World: An Annotated and Illustrated Catalogue of Shark Species Known to Date. Part 1: Hexanchiformes to Lamniformes.

[18] Vooren C.M., da Silva K.G. (1991). On the taxonomy of the angel sharks from southern Brazil, with the description of *Squatina occulta* sp. n. Rev. Brasil Biol..

[19] Milessi A., Vögler R., Bazzino G. (2001). Identificación de tres especies del genero *Squatina* (Chondrichthyes, Squatinidae) en la Zona Común de Pesca Argentino-Uruguaya (ZCPAU). Gayana.

[20] Castro Aguirre J.L., Espinosa Pérez H., Huidobro Campos L. (2006). Dos nuevas especies del género *Squatina* (Chondrichthyes: Squatinidae) del Golfo de México. Rev. Biol. Trop..

[21] Walsh J.H., Ebert D.A. (2007). A review of the systematics of western North Pacific angel sharks, genus *Squatina*, with redescriptions of *Squatina formosa*, *S. japonica*, and *S. nebulosa* (Chondrichthyes: Squatiniformes, Squatinidae). Zootaxa.

[22] Last P.R., White W.T. (2008). Three new angel sharks (Chondrichthyes: Squatinidae) from the Indo-Australian region. Zootaxa.

[23] Walsh J.H., Ebert D.A., Compagno L.J.V. (2011). *Squatina caillieti* sp. nov., a new species of angel shark (Chondrichthyes: Squatiniformes: Squatinidae) from the Philippine Islands. Zootaxa.

[24] Vaz D.F.B., de Carvalho M.R. (2013). Morphological and taxonomic revision of species of *Squatina* from the Southwestern Atlantic Ocean (Chondrichthyes: Squatiniformes: Squatinidae). Zootaxa.

[25] Compagno L.J.V., Stehmann M., Ebert D.A. (1990). *Rhinochimaera africana*, a new longnose chimaera from southern Africa, with comments on the systematics and distribution of the genus *Rhinochimaera* Garman, 1901 (Chondrichthyes, Chimaeriformes, Rhinochimaeridae). S. Afr. J. Mar. Sci..

[26] Sabaj M.H. (2022). Codes for Natural History Collections in Ichthyology and Herpetology (Online Supplement).

[27] Weigmann S., Gon O., Leeney R.H., Barrowclift E., Berggren P., Jiddawi N., Temple A.J. (2020). Revision of the sixgill sawsharks, genus *Pliotrema* (Chondrichthyes, Pristiophoriformes), with descriptions of two new species and a redescription of *P. warreni* Regan. PLoS ONE.

[28] Weigmann S., Ebert D.A., Séret B. (2021). Resolution of the *Acroteriobatus leucospilus* species complex, with a redescription of *A. leucospilus* (Norman, 1926) and descriptions of two new western Indian Ocean species of *Acroteriobatus* (Rhinopristiformes, Rhinobatidae). Mar. Biodivers..

[29] Kaschner C.J., Weigmann S., Thiel R. (2015). *Bythaelurus tenuicephalus* n. sp., a new deep-water catshark (Carcharhiniformes, Scyliorhinidae) from the western Indian Ocean. Zootaxa.

[30] Weigmann S., Ebert D.A., Clerkin P.J., Stehmann M.F.W., Naylor G.J.P. (2016). *Bythaelurus bachi* n. sp., a new deep-water catshark (Carcharhiniformes, Scyliorhinidae) from the southwestern Indian Ocean, with a review of *Bythaelurus* species and a key to their identification. Zootaxa.

[31] Weigmann S., Kaschner C.J., Thiel R. (2018). A new microendemic species of the deep-water catshark genus *Bythaelurus* (Carcharhiniformes, Pentanchidae) from the northwestern Indian Ocean, with investigations of its feeding ecology, generic review and identification key. PLoS ONE.

[32] Weigmann S., Kaschner C.J. (2017). *Bythaelurus vivaldii*, a new deep-water catshark (Carcharhiniformes, Scyliorhinidae) from the northwestern Indian Ocean off Somalia. Zootaxa.

[33] Marramà G., Kriwet J. (2017). Principal component and discriminant analyses as powerful tools to support taxonomic identification and their use for functional and phylogenetic signal detection of isolated fossil shark teeth. PLoS ONE.

[34] Vaz D.F.B. (2021). *Scymnodon plunketi* (Waite, 1910): A junior synonym of *Scymnodon macracanthus* (Regan, 1906) (Somniosidae: Elasmobranchii). J. Fish. Biol..

[35] Da Silva J.P.C.B., de Carvalho M.R. (2015). Morphology and phylogenetic significance of the pectoral articular region in elasmobranchs (Chondrichthyes). Zool. J. Linn. Soc..

[36] Da Silva J.P.C.B., Vaz D.F.B. (2023). Morphology and phylogenetic significance of the pelvic articular region in elasmobranchs (Chondrichthyes). Cladistics.

[37] Compagno L.J.V. (1988). Sharks of the Order Carcharhiniformes.

[38] Amante C., Eakins B.W. (2009). ETOPO1 1 Arc-Minute Global Relief Model: Procedures, Data Sources and Analysis. NOAA. Tech. Mem. NEDIS NGDC-24.

[39] Weigmann S., Stehmann M.F.W., Thiel R. (2013). *Planonasus parini* n. g. and n. sp., a new genus and species of false cat sharks (Carchariniformes, Pseudotriakidae) from the deep northwestern Indian Ocean off Socotra Islands. Zootaxa.

[40] Weigmann S., Stehmann M.F.W., Thiel R. (2015). *Okamejei ornata* n. sp., a new deep-water skate (Elasmobranchii, Rajidae) from the northwestern Indian Ocean off Socotra Islands. Deep-Sea Res. II Top. Stud. Oceanogr..

[41] Krajangdara T., Khudamrongsawat J., Chaorattana C., Promnun P., Weigmann S. (2021). Morphological and molecular examinations of a northwestern Indian Ocean population of the African angelshark, *Squatina* cf. *africana* Regan, 1908 (Chondricthyes: Squatiniformes: Squatinidae), with remarks on intraspecific variations. Phuket Mar. Biol. Cent. Res. Bull..

[42] Gubanov E.P., Kondyurin V.V., Myagkov N.A. (1986). Akuly Mirovogo Okeana: Spravochnik-Opredelitel (Sharks of the World Ocean: Identification Handbook).

[43] de Baissac J.B. (1990). SWIOP/WP/54—Checklist of the Marine Fishes of Mauritius.

[44] Gubanov E.P. (1993). Akuly Indiĭskogo Okeana: Atlas-Opredelitel.

[45] de Baissac J.B. (1976). Poissons de mer des eaux de l’Ile Maurice. Proc. Roy. Soc. Arts Maurit..

[46] Fricke R. (1999). Fishes of the Mascarene Islands (Réunion, Mauritius, Rodriguez): An Annotated Checklist with Descriptions of New Species.

[47] Manilo L.G., Bogorodsky S.V. (2003). Taxonomic composition, diversity and distribution of coastal fishes of the Arabian Sea. J. Ichthyol..

[48] Reeve A.J., Kayoueche-Reeve M., Al-Mamari T., Al-Shuaily S., Henderson A.C. (2011). A Field Guide to the Elasmobranchs of South-East Arabia. Part One: Sharks.

[49] Akhilesh K.V., Bineesh K.K., Gopalakrishnan A., Jena J.K. (2014). Checklist of Chondrichthyans in Indian waters. J. Mar. Biol. Ass. India.

[50] Jawad L.A. (2018). Dangerous Fishes of the Eastern and Southern Arabian Peninsula.

[51] Ambily M.N., Zacharia P.U., Najmudeen T.M., Ambily L., Sunil K.T.S., Radhakrishnan M., Kishor T.G. (2018). First Record of African Angel Shark, *Squatina africana* (Chondricthyes: Squatinidae) in Indian Waters, Confirmed by DNA Barcoding. J. Ichthyol..

[52] Joshi K.K., Balachandran K., Raje S.G. (2008). Changes in the shark fishery at Cochin. J. Mar. Biol. Ass. India.

[53] Strasburg D.W. (1963). The Diet and Dentition of *Isistius brasiliensis*, with Remarks on Tooth Replacement in Other Sharks. Copeia.

[54] Shelmerdine R.L., Cliff G. (2006). Sharks caught in the protective gill nets off KwaZulu-Natal, South Africa. 12. The African angel shark *Squatina africana* (Regan). Afr. J. Mar. Sci..

[55] Fennessy S.T. (1994). Incidental capture of elasmobranchs by commercial prawn trawlers on the Tugela Bank, Natal, South Africa. S. Afr. J. Mar. Sci..

[56] Bass A.J., D’Aubrey J.D.D., Kistnasamy N. (1975). Sharks of the east coast of southern Africa. V. The families Hexanchidae, Chlamydoselachidae, Heterodontidae, Pristiophoridae and Squatinidae. Invest. Rep. Oceanogr. Res. Inst. Durban.

[57] Aura C.M., Munga C.N., Kimani E., Manyala J.O., Musa S. (2011). Length—Weight Relationships for Nine Deep Sea Fish Species off the Kenyan Coast. Pan-Am. J. Aquat..

[58] Kiilu K.B., Ndegwa S. (2013). Shark Bycatch—Small Scale Tuna Fishery Interactions along the Kenyan Coast.

[59] Kiilu B.K., Kaunda-Arara B., Oddenyo R.M., Thoya P., Njiru J.M. (2019). Spatial distribution, seasonal abundance and exploitation status of shark species in Kenyan coastal waters. Afr. J. Mar. Sci..

[60] Shehe M.A., Jiddawi N.S., Fowler S.L., Reed T.M., Dipper F.A. The Status of Shark Fisheries in Zanzibar. Elasmobranch Biodiversity, Conservation and Management (Proceedings of the International Seminar and Workshop, Sabah, Malaysia, July 1997).

[61] Smith J.L.B. (1949). The Sea Fishes of Southern Africa.

[62] Iselstöger H. (1937). Das Neurocranium von *Rhina squatina* und einige Bemerkungen über ihre systematische Stellung. Zool. Jahrbücher Abt. Fur Anat. Und Ontog. Der Tiere.

[63] Compagno L.J.V. (1977). Phyletic Relationships of Living Sharks and Rays. Am. Zool..

[64] Capapé C., Roux C. (1980). Étude anatomique du neurocrane, de la ceinture pelvienne et des ptérigiopodes des Squatinidae (Pisces, Pleurotremata) des côtes tunisiennes. Bull. Mus. Natl. Hist. Nat..

[65] de Carvalho M.R., Kriwet J., Thies D., Arratia G., Schultze H.P., Wilson M.H.V. (2008). A Systematic and Anatomical Revision of Late Jurassic Angelsharks (Chondrichthyes: Squatinidae). Mesozoic Fishes 4, Homology and Phylogeny.

[66] de Carvalho M.R., Faro C., Gomes U.L. (2012). Comparative neurocranial morphology of angelsharks from the south-western Atlantic Ocean (Chondrichthyes, Elasmobranchii, Squatinidae): Implications for taxonomy and phylogeny: Systematic implications of *Squatina* neurocrania. Acta Zool..

[67] Da Silva J.P.C.B., Vaz D.F.B., de Carvalho M.R. (2015). Systematic Implications of the Anterior Pectoral Basals in Squaliform Sharks (Chondrichthyes: Elasmobranchii). Copeia.

[68] Da Silva J.P.C.B., Vaz D.F.B., de Carvalho M.R. (2018). Phylogenetic inferences on the systematics of squaliform sharks based on elasmobranch scapular morphology (Chondrichthyes: Elasmobranchii). Zool. J. Linn. Soc..

[69] López-Romero F.A., Stumpf S., Pfaff C., Marramà G., Johanson Z., Kriwet J. (2020). Evolutionary trends of the conserved neurocranium shape in angel sharks (Squatiniformes, Elasmobranchii). Sci. Rep..

[70] Mollen F.H., Van Bakel B.W.M., Jagt J.W.M. (2016). A partial braincase and other skeletal remains of Oligocene angel sharks (Chondrichthyes, Squatiniformes) from northwest Belgium, with comments on squatinoid taxonomy. Contrib. Zool..

[71] Marples B.J. (1936). The blood vascular system of the Elasmobranch fish *Squatina squatina* (Linné). Trans. R. Soc. Edinb..

[72] Miyake T., Mceachran J.D. (1991). The morphology and evolution of the ventral gill arch skeleton in batoid fishes (Chondrichthyes: Batoidea). Zool. J. Linn. Soc..

[73] Da Silva J.P.C.B., Datovo A. (2020). The coracoid bar and its phylogenetic importance for elasmobranchs (Chondrichthyes). Zool. Anz..

[74] Stelbrink B., Von Rintelen T., Cliff G., Kriwet J. (2010). Molecular systematics and global phylogeography of angel sharks (genus *Squatina*). Mol. Phylogenet. Evol..

[75] Dulvy N.K., Fowler S.L., Musick J.A., Cavanagh R.D., Kyne P.M., Harrison L.R., Carlson J.K., Davidson L.N., Fordham S.V., Francis M.P. (2014). Extinction risk and conservation of the world’s sharks and rays. eLife.

[76] Dulvy N.K., Pacoureau N., Rigby C.L., Pollom R.A., Jabado R.W., Ebert D.A., Finucci B., Pollock C.M., Cheok J., Derrick D.H. (2021). Overfishing drives over one-third of all sharks and rays toward a global extinction crisis. Curr. Biol..

[77] Lawson J.M., Pollom R.A., Gordon C.A., Barker J., Meyers E.K.M., Zidowitz H., Ellis J.R., Bartolí Á., Morey G., Fowler S.L. (2020). Extinction risk and conservation of critically endangered angel sharks in the Eastern Atlantic and Mediterranean Sea. ICES J. Mar. Sci..

[78] Giovos I., Stoilas V., Al-Mabruk S.A., Doumpas N., Marakis P., Maximiadi M., Moutopoulos D., Kleitou P., Keramidas I., Tiralongo F. (2019). Integrating local ecological knowledge, citizen science and long-term historical data for endangered species conservation: Additional records of angel sharks (Chondrichthyes: Squatinidae) in the Mediterranean Sea. Aquatic Conserv. Mar. Freshw. Ecosyst..

[79] Hiddink J.G., Shepperson J., Bater R., Goonesekera D., Dulvy N.K. (2019). Near disappearance of the Angelshark *Squatina squatina* over half a century of observations. Conserv. Sci. Pract..

[80] Shephard S., Wögerbauer C., Green P., Ellis J.R., Roche W. (2019). Angling records track the near extirpation of angel shark *Squatina squatina* from two Irish hotspots. Endanger. Species Res..

[81] IUCN (2022). The IUCN Red List of Threatened Species.

[82] Cliff G., Bennett R., Da Silva C., Ebert D.A., Fennessy S., Gledhill K., Jabado R.W., Kuguru B., Leslie R., McCord M.E. (2019). Squatina africana. The IUCN Red List of Threatened Species: e.T44996A113073072.

[83] Schaeffer D. (2004). Assessment of the Artisanal Shark Fishery and Local Shark Fin Trade on Unguja Island, Zanzibar.

[84] Barrowclift E., Temple A.J., Stead S., Jiddawi N.S., Berggren P. (2017). Social, economic and trade characteristics of the elasmobranch fishery on Unguja Island, Zanzibar, East Africa. Mar. Policy.

[85] Temple A.J., Wambiji N., Poonian C.N.S., Jiddawi N., Stead S.M., Kiszka J.J., Berggren P. (2019). Marine megafauna catch in southwestern Indian Ocean small-scale fisheries from landings data. Biol. Conserv..

[86] Humber F., Andriamahaino E.T., Beriziny T., Botosoamananto R., Godley B.J., Gough C., Pedron S., Ramahery V., Broderick A.C. (2017). Assessing the small-scale shark fishery of Madagascar through community-based monitoring and knowledge. Fish. Res..

[87] Temple A.J., Kiszka J.J., Stead S.M., Wambiji N., Brito A., Poonian C.N.S., Amir O.A., Jiddawi N., Fennessy S.T., Pérez-Jorge S. (2018). Marine megafauna interactions with small-scale fisheries in the southwestern Indian Ocean: A review of status and challenges for research and management. Rev. Fish. Biol. Fish..

[88] Pollom R., Cheok J., Pacoureau N., Gledhill K.S., Kyne P.M., Ebert D.A., Jabado R.W., Herman K.B., Bennett R.H., Silva C. (2021). Overfishing and Climate Change Elevate Extinction Risk of Endemic Sharks and Rays in the Southwest Indian Ocean Hotspot. Res. Sq. Platf..

[89] Jabado R.W., Kyne P.M., Pollom R.A., Ebert D.A., Simpfendorfer C.A., Ralph G.M., Al Dhaheri S.S., Akhilesh K.V., Ali K., Ali M.H. (2018). Troubled waters: Threats and extinction risk of the sharks, rays and chimaeras of the Arabian Sea and adjacent waters. Fish Fish..

[90] John M.E., Varghese B.C. (2009). Decline in CPUE of Oceanic Sharks in the Indian EEZ: Urgent Need for Precautionary Approach.

